# *In silico* analysis of the HSP90 chaperone system from the African trypanosome, *Trypanosoma brucei*

**DOI:** 10.3389/fmolb.2022.947078

**Published:** 2022-09-23

**Authors:** Miebaka Jamabo, Stephen John Bentley, Paula Macucule-Tinga, Praise Tembo, Adrienne Lesley Edkins, Aileen Boshoff

**Affiliations:** ^1^ Biotechnology Innovation Centre, Rhodes University, Grahamstown, South Africa; ^2^ Department of Biochemistry and Microbiology, Biomedical Biotechnology Research Unit (BioBRU), Rhodes University, Grahamstown, South Africa

**Keywords:** African trypanosomiasis, *Trypanosoma brucei*, HSP90, molecular chaperones, co-chaperone, heat shock proteins, HSP83

## Abstract

African trypanosomiasis is a neglected tropical disease caused by *Trypanosoma brucei* (*T. brucei*) and spread by the tsetse fly in sub-Saharan Africa. The trypanosome relies on heat shock proteins for survival in the insect vector and mammalian host. Heat shock protein 90 (HSP90) plays a crucial role in the stress response at the cellular level. Inhibition of its interactions with chaperones and co-chaperones is being explored as a potential therapeutic target for numerous diseases. This study provides an *in silico* overview of HSP90 and its co-chaperones in both *T. brucei brucei* and *T. brucei gambiense* in relation to human and other trypanosomal species, including non-parasitic *Bodo saltans* and the insect infecting *Crithidia fasciculata*. A structural analysis of *T. brucei* HSP90 revealed differences in the orientation of the linker and C-terminal domain in comparison to human HSP90. Phylogenetic analysis displayed the *T. brucei* HSP90 proteins clustering into three distinct groups based on subcellular localizations, namely, cytosol, mitochondria, and endoplasmic reticulum. Syntenic analysis of cytosolic *HSP90* genes revealed that *T. b. brucei* encoded for 10 tandem copies, while *T. b. gambiense* encoded for three tandem copies; *Leishmania major (L. major)* had the highest gene copy number with 17 tandem copies. The updated information on HSP90 from recently published proteomics on *T. brucei* was examined for different life cycle stages and subcellular localizations. The results show a difference between *T. b. brucei* and *T. b. gambiense* with *T. b. brucei* encoding a total of twelve putative *HSP90* genes, while *T. b. gambiense* encodes five *HSP90* genes. Eighteen putative co-chaperones were identified with one notable absence being cell division cycle 37 (Cdc37). These results provide an updated framework on approaching HSP90 and its interactions as drug targets in the African trypanosome.

## 1 Introduction

*Trypanosoma brucei* (*T. brucei*), is an extracellular blood- and tissue-borne protozoan parasite transmitted by *Glossina* (tsetse) fly vectors, which causes devastating diseases in humans, wild animals, and domesticated livestock (Brun et al., 2010). Human African trypanosomiasis (HAT, also known as African sleeping sickness) is a potentially fatal tropical disease found in remote rural regions of sub-Saharan Africa and often coincides with insubstantial health care systems (Fèvre et al., 2008). HAT is caused by two subspecies of *T. brucei*; the chronic form of the disease, which is endemic to Central and Western Africa, is caused by *Trypanosoma brucei* (*T. b.*) *gambiense*, and the acute zoonotic form, which is endemic to Eastern and Southern Africa, is caused by *T. b. rhodesiense* (Simarro et al., 2010; Büscher et al., 2017). The livestock disease, nagana, is caused by *T. b. brucei* and has been shown, along with *T. congolense* and *T. vivax*, to have a crippling effect on socioeconomic development within sub-Saharan Africa (Alsan, 2015; Morrison et al., 2016). Recently, atypical human trypanosomiasis was reported to have emerged, with animal *Trypanosoma* species increasingly being detected in humans (Kumar et al., 2022). Despite the decreasing number of HAT cases and the first recently approved oral treatment called fexinidazole, which has now been added to the updated WHO guidelines as the recommended treatment for first and second stages *T* *b*. *gambiense* HAT (Deeks, 2019; WHO, 2019; Lindner et al., 2020). There is still a need for the development of new and more effective drugs due to lack of a vaccine and increasing parasite resistance (Barrett and Croft, 2012). Molecular chaperones have been identified as an attractive target for drug development against protozoan parasites as this protein family plays essential roles in stress-induced stage differentiation and are vital for disease progression and transmission (Requena et al., 2015; Bentley et al., 2019; Zininga and Shonhai, 2019).

The 90-kDa heat shock protein (HSP90) family contains essential, highly conserved, and abundant molecular chaperones (Csermely et al., 1998; Chen et al., 2006; Johnson, 2012) that facilitate the proper folding and maturation of a large but specific group of substrates called client proteins (Jakob et al., 1995; Hartl et al., 2011; Hoter et al., 2018). More than 400 client proteins of human HSP90 have been identified to date (listed at http://www.picard.ch/), with many of them being implicated in protein folding and degradation, signaling pathways, cellular trafficking, cell cycle regulation, and differentiation (Echeverría et al., 2011; Samant et al., 2012; Taipale et al., 2012). In humans, the HSP90 family normally comprises four isoforms that are localized in various cellular compartments. Two HSP90 isoforms (the stress-inducible form HSP90α/HSPC2 and the constitutive form HSP90β/HSPC3) are localized in the cytosol and in the nucleus (Subbarao Sreedhar et al., 2004; Kampinga et al., 2009; Li et al., 2012); GRP94/HSPC4 is present in the endoplasmic reticulum (ER) (Subbarao Sreedhar et al., 2004; Kampinga et al., 2009; Marzec et al., 2012) and TRAP-1/HSPC5 is found in the mitochondrial matrix (Altieri et al., 2012). Some intracellular HSP90 isoforms are exported and functioned in the extracellular environment to regulate the immune response, cell migration, and invasion (Binder, 2014; Hance et al., 2014; Hunter et al., 2014; Baker-Williams et al., 2019).

HSP90 is a flexible dimeric protein with each monomer containing three domains: an N-terminal nucleotide-binding domain (NBD), a middle client protein-binding domain (MD), and a C-terminal domain (CTD) (Whitesell and Lindquist, 2005; Buchner and Li, 2013; Jackson, 2013). HSP90 is dependent on ATP hydrolysis, and a set of accessory proteins termed co-chaperones, which assist in the recruitment of client proteins and the regulation of the HSP90 reaction cycle (Prodromou, 2012; Röhl et al., 2013). The cytosolic HSP90 isoforms contain a conserved C-terminal MEEVD motif, which acts as a docking site for interaction with co-chaperones that possess the tetratricopeptide repeat (TPR-) domain (Blatch and Lässle, 1999; Prodromou, 1999). Other HSP90 co-chaperones interact with the molecular chaperone through its NBD or M domain (Röhl et al., 2013). Fifty co-chaperones have been identified in the mammalian HSP90 chaperone system to date (Dean and Johnson, 2021): 23 TPR co-chaperones have been characterized, 18 cysteine and histidine-rich domain (CHORD) or SGT1 (CS) domain co-chaperones, and eight co-chaperones without these two domains (Garcia-Ranea et al., 2002; Schopf et al., 2017; Dean and Johnson, 2021). However, the composition of the HSP90 chaperone system appears to vary across organisms, indicating that the role of some co-chaperones may be necessary for activating client proteins in a species-dependent manner, be replaceable with other co-chaperones, or be limited to a distinct subgroup of client proteins (Zuehlke and Johnson, 2010). HSP90 is also subject to post-translational modifications, including s-nitrosylation, phosphorylation, and acetylation, which may influence its activity, cellular localization, or its interaction with co-chaperones, nucleotides, or client proteins (Aoyagi and Archer, 2005; Duval et al., 2007; Rao et al., 2008; Yang et al., 2008). Some HSP90 isoforms are essential for viability, and maintenance of client proteins that are dependent on the chaperone (Citri et al., 2004), making it an attractive drug target for diseases including infectious diseases. Several HSP90 inhibitors, which have been well-studied in the laboratory and clinics for antitumor indications (Porter et al., 2010; Trepel et al., 2010), were also shown to arrest the growth of several kinetoplastids *in vitro* and have activity against *Trypanosoma evansi* and *T. brucei* in mice (Graefe et al., 2002; Pallavi et al., 2010; Meyer and Shapiro, 2013; Meyer et al., 2018). Thus, the repurposing of HSP90 inhibitors designed for cancer treatment is one strategy to evaluate new and effective antitrypanosomal agents (Kaiser et al., 2015).

In *Trypanosoma* and *Leishmania*, the HSP90 machinery plays a pivotal role in environmental sensing and life cycle control (Ploeg et al., 1985; Wiesgigl and Clos, 2001; Graefe et al., 2002). *In silico* analyses of the HSP90/HSPC family of intracellular kinetoplastid parasites has been published (Shonhai et al., 2011; Roy et al., 2012; Figueras et al., 2014; Urményi et al., 2014; Requena et al., 2015), and our study provides an updated and comprehensive analysis for the extracellular parasite, *T. brucei. T. brucei* exhibits a digenetic lifestyle, and therefore must adapt to fluctuating environmental conditions, such as change in temperature, pH, nutrients, and the pressure from the immune system, as it transitions from the gut of the tsetse fly to the body fluids of its mammalian host (Jones et al., 2008; Roy et al., 2012). A distinct molecular trait of trypanosomes is their dependence on polycistronic transcription akin to prokaryotes, their mRNAs are mainly generated by trans-splicing and there is a dependence on post-transcriptional mechanisms for gene regulation (Preußer et al., 2012). However, correlation studies comparing the previously reported RNA-seq data of transcript abundance and proteomic data from the procyclic form (PF) and bloodstream form (BSF) of the parasite shows that the differences observed between the PF and BSF are two-fold greater at the proteomic level when compared to the transcriptomic level (Urbaniak et al., 2012; Butter et al., 2013). Given the complexities of transcription, its incomplete representation of the life cycle stages of the parasite as well as its lack of control, trypanosome research has largely shifted to rely on proteomic data (Goos et al., 2017). Numerous proteomic studies have been conducted on the parasite, which have compared protein expression at the different life cycle stages (Gunasekera et al., 2012; Urbaniak et al., 2012; Butter et al., 2013), in the mitochondrion (Panigrahi et al., 2009), mitochondrial importome (Peikert et al., 2017), respiratome (Acestor et al., 2011), mitochondrial membranes (outer, intermembrane space, inner, and matrix) (Acestor et al., 2009), nucleus (Goos et al., 2017), nuclear pore (DeGrasse et al., 2008), glycosomes (Colasante et al., 2006; Güther et al., 2014), flagellum (Broadhead et al., 2006; Subota et al., 2014), and the cell surface (Shimogawa et al., 2015).

*T. brucei* and other trypanosomatids rely on post-translational modifications (PTMs) to increase their proteome diversity and complexity (Backe et al., 2020). Several studies exploring the phosphoproteome and acetylome of trypanosomes (Nett et al., 2009a, 2009b; Urbaniak et al., 2013; Moretti et al., 2018) have found that phosphorylation and acetylation are the most predominant modifications to occur in *T. brucei* proteins. Both PTMs are well known for impacting HSP90 intracellular localization in humans as well as their ability to bind co-chaperones, nucleotides, clients (Nett et al., 2009a; Backe et al., 2020), and even inhibitors (Zhang et al., 2020). However, the PTMs regulatory dynamic in the organellar TRAP-1 and GRP94 in humans are yet to be elucidated for a global understanding of this critical chaperone activity regulator system.

The aim of this study was to provide a comprehensive depiction of the *T. brucei* HSP90 chaperone system based on structural, functional, and evolutionary analyses. *In silico* tools were used to evaluate the domain conservation, predicted subcellular localizations, syntenic, and phylogenetic analysis of the HSP90 chaperone system in *T. brucei* with respect to both *T. b. brucei* and *T. b. gambiense*. The HSP90 chaperone system was also comparatively analyzed in relation to those found in selected trypanosomastid parasites and *Homo sapiens* (*H. sapiens*). The proteomic findings on HSP90 and its co-chaperones from the numerous published proteomic data on *T. brucei* are presented, and we provide updated insights on the adaptability of the parasite from its stage-specific expressed proteins and provide an overall context for identifying new and potential drug targets for HAT.

## 2 Materials and methods

### 2.1 Database mining, sequence analyses, and the determination of the trypanosomastid and human orthologues

A BLASTP search using the amino acid sequences of the HSP90 isoforms from *T b. brucei* obtained from previous *in silico* study (Folgueira and Requena, 2007), and the human HSP90AA1/HSPC1, HSPC2, HSP90AB1/HSPC3, HSP90B1/GRP94/HSPC4, and TRAP-1/HSPC5 isoforms were used as queries on the TriTrypDB (version 46) database (http://tritrypdb.org/tritrypdb/) (Aslett et al., 2010) and were conducted in order to determine the HSP90/HSPC complement encoded on the *T. b. gambiense* genome, as well as identify new *T. b. brucei* HSP90/HSPC protein members. The e-value was set at an intermediately stringent level of e-10 to identify HSP90/HSPC-related sequences for further analysis. In addition, a keyword search was performed to scan the genome of *T b. gambiense* for HSP90/HSPC genes on the TriTrypDB database using the terms: “HSP90,” “HSP83,” “heat shock protein,” and “molecular chaperone.” The retrieved amino acid sequences from the various keyword searches were then screened using SMART 7 (Simple Modular Architecture Research Tool; http://smart.embl-heidelberg.de/) (Letunic et al., 2012) and PROSITE (http://prosite.expasy.org/) (Sigrist et al., 2010) for domains annotated by the online servers as “HSP90.” Incomplete sequences for each protein from TriTrypDB were omitted for construction of Table 1.

**TABLE 1:** HSP90/HSPC proteins from *Trypanosoma brucei* with putative orthologues in *T. cruzi*, *L. major*, *C. fasciculata*, *B. saltans*, and *H. sapiens*.

*H. sapiens*	*H. sapiens*	*T. brucei*	*T. cruzi* ^*c*^	*L. major*	*C. fasciculata*	*B. saltans*
Name^*a*^	Gene ID^*b*^	Gene ID^*b*^	Gene ID^*b*^	Gene ID^*b*^	Gene ID^*b*^	Gene ID^*b*^	Localization^*d*^	Reference
HSP90-alpha/HSPC2	3324	Tb927.10.10890			CFAC1_280011900 CFAC1_280012000	BSAL_87515		Gunasekera et al. (2012); Urbaniak et al. (2012); Subota et al. (2014); Shimogawa et al. (2015); Dean et al. (2017)
		Tb927.10.10900						
		Tb927.10.10910	TcCLB.507713.30	LmjF.33.0312 LmjF.33.0314 LmjF.33.0316 LmjF.33.0318 LmjF.33.0320 LmjF.33.0323 LmjF.33.0326 LmjF.33.0330 LmjF.33.0333 LmjF.33.0336 LmjF.33.0340 LmjF.33.0343				
	3326	Tb927.10.10920	C4B63_113g25				CYT	
HSP90-beta/HSPC3		Tb927.10.10930 Tb927.10.10940	C4B63_113g29				NUC	
		Tb927.10.10950 Tb927.10.10960 Tb927.10.10970 Tb927.10.10980	C4B63_113g30 C4B63_113g33 C4B63_84g87 C4B63_84g88 C4B63_84g89				FLAGELLAR	
		Tbg972.10.13260 Tbg972.10.13270 Tbg972.10.13280	Tc_MARK_3,581	LmjF.33.0346 LmjF.33.0350 LmjF.33.0355 LmjF.33.0360 LmjF.33.0365			CELL SURFACE	
GRP94/HSPC4	7184	Tb927.3.3580 Tbg972.3.3850	C4B63_10g439 Tc_MARK_3058	LmjF.29.0760	CFAC1_1,00018800	BSAL_88715	ER	Gunasekera et al. (2012); Urbaniak et al. (2012); Subota et al. (2014); Shimogawa et al. (2015)
							NUC	
							FLAGELLAR	
							CELL SURFACE	
TRAP-1/HSPC5	10131	Tb927.11.2650 Tbg972.11.2900	TcCLB.504153.310	LmjF33.2390	CFAC1_230028300	BSAL_33145		Panigrahi et al. (2009); Subota et al. (2014); Dean et al. (2017)
			C4B63_2g430				MITO	
			Tc_MARK_6238				FLAGELLAR	

a The nomenclature for the HSP90/HSPC, proteins from T b. brucei, and T b. gambiense were derived according to Folgueira and Requena (2007).

b The Gene IDs for the members of the *T b. brucei* (Tb refers to Tbb), *T b. gambiense*, *T. cruzi*, *C. fasciculata*, *B. saltans*, and *L. major* HSP90/HSPC protein family were retrieved from the TriTrypDB database (http://tritrypdb.org/tritrypdb/; Aslett et al., 2010). The Gene IDs for the members of the *H. sapiens* HSP90/HSPC protein family were retrieved from NCBI (https://www.ncbi.nlm.nih.gov/).

c The Gene IDs, for the orthologues, identified by reciprocal BLASTP, analysis, of three strains of *T. cruzi* are listed. *T. cruzi* CL, Brener Esmeraldo-like (TcCLB), *T. cruzi* Dm28c 2018 (C4B63), and *T. cruzi* marinkelli strain B7 (Tc_MARK).

d Subcellular localizations for the *T. brucei* HSP90/HSPC proteins were either acquired from using the TrypTag database (http://tryptag.org/;Dean, Sunter, and Wheeler 2017) and/or predicted using various proteomic datasets and online prediction software listed in the materials and methods.

CYT-Cytosol and MITO- mitochondrion, CYT-Cytosol; MITO- mitochondrion; NUC- nucleus; ER- endoplasmic reticulum; GYLCO-glycosomes; FLAGELLAR- flagellar; CELL SURFACE- cell surface.

For identification of *T. brucei* orthologues of selected cytosolic HSP90 co-chaperones, the protein sequences of 50 human co-chaperones were used as queries in a BLASTP search on the TriTrypDB database. Reciprocal BLASTP was conducted to determine if the identified putative *T. brucei* co-chaperone had the closest match to the desired human co-chaperone. The putative amino acid sequences of the co-chaperones from both *T. brucei* subspecies were used as queries in a BLASTP search on the National Centre for Biotechnology Information (NCBI) website (www.ncbi.nlm.nih.gov), using the default parameters. If the most similar ortholog in the *T. brucei* subspecies was identical to the HSP90 co-chaperones sequence used as the first query, the sequence of the second query was selected as an ortholog. Reciprocal BLASTP was also conducted for the identification of human and selected trypanosomastid orthologues of the putative HSP90/HSPC and HSP90 co-chaperone proteins from both *T. brucei* subspecies.

### 2.2 Structural analysis of TbHSP83

The retrieved amino acid sequences for hHsp90β (NP_001258899.1), TbbHsp83 (Tb927.10.10890), and TbgHsp83 (Tbg972.10.13260) were analyzed using Jalview (Waterhouse et al., 2009). A multiple sequence alignment was conducted using Clustal with defaults *via* the Jalview web service. 3D prediction structures were retrieved from the AlphaFold database (https://alphafold.ebi.ac.uk/) by querying the database using the UniProt IDs of the respective proteins. The retrieved structures (TbHsp83- AF-Q389P1-F1; hHsp90 - AF-P08238-F1) were visualized and superimposed using PyMOL molecular graphics system, Version 2.0 Schrödinger, LLC. The FATCAT server (https://fatcat.godziklab.org/) was used to analyze the structural differences between the protein homologs (Li et al., 2020). The two structures were determined to be significantly similar with a *p*-value of 0.00 (raw FATCAT score 1931.78), 685 equivalent positions, and RMSD of 1.71 Å and two twists.

### 2.3 Phylogenetic and conserved syntenic analysis

The full-length amino acid sequences for the HSP90/HSPC family in the selected trypanosomastid parasites were obtained from the TriTrypDB database (Aslett et al., 2010), and the human protein sequences were obtained from the NCBI website (www.ncbi.nlm.nih.gov). Accession numbers for the HSP90/HSPC amino acid sequences used in this study are provided in Table 1 and Supplementary Table S1. Multiple sequence alignments were performed using the inbuilt ClustalW program (Larkin et al., 2007) with default parameters in MEGA-X (Kumar et al., 2018) and are listed in Supplementary Figure S1. Maximum-likelihood (ML) was utilized to find the best model of evolution and was selected by the Bayesian information criterion (BIC) implemented in MEGA-X. The amino acid-based HSP90/HSPC ML phylogeny was reconstructed using the JTT (Jones–Taylor–Thornton) model matrix (Jones et al., 1992), with gamma distribution shape parameter (G). The ML phylogenetic tree was constructed using MEGA-X (Kumar et al., 2018). The accuracy of the reconstructed tree was assessed using a bootstrap test using 1,000 replicates with a pairwise gap deletion mode. The phylogenetic tree for HSP90s was unrooted.

The putative HSP90 genes in the three *T. cruzi* strains homologous to HSP83 identified to be partial, and/or truncated genes were included in the syntenic analysis. Syntenic analysis was conducted to evaluate the conservation of the gene arrangement of the cytosolic HSP83 genes in *T. brucei* and selected trypanosomastid parasites. The conserved syntenic regions surrounding the selected HSP83 genes were searched by examining the conserved co-localization of neighboring genes on a scaffold of the *T. brucei* subspecies (*T. b. brucei* and *T. b. gambiense*) and selected trypanosomastid parasites for this study using genome information from the TriTrypDB database. The identities of unknown neighbor genes of the selected HSP83 genes were conducted using a BLASTP search on the NCBI database.

### 2.4 Physiochemical properties, protein expression, and the determination of the organelle distribution for the *T. brucei* HSP90/HSPC complement

The physiochemical properties, molecular weight (Da), and isoelectric point (pI) of each putative protein was determined using the compute pI/Mw tool from ExPASy (https://web.expasy.org/compute_pi/) (Gasteiger et al., 2005). Data on the previously reported phenotypic RNAi knockdown screen (Alsford et al., 2011), for each member of the HSP90/HSPC complement and identified HSP83 co-chaperones, were retrieved from the TrypsNetDB database (http://trypsnetdb.org/QueryPage.aspx) (Gazestani et al., 2017). The organelle distribution for each putative protein was searched using the TrypTag microscopy project’s online server (http://tryptag.org/) (Dean et al., 2017). This project aims at tagging every trypanosome protein with mNeonGreen (mNG) (Shaner et al., 2013) to determine the protein’s localization within the parasite. Proteomic data from the mitochondrion (Panigrahi et al., 2009), mitochondrial importome (Peikert et al., 2017), respiratome (Acestor et al., 2009), mitochondrial membranes (outer, intermembrane space, inner, and matrix) (Acestor et al., 2009), nucleus (Goos et al., 2017), nuclear pore (DeGrasse et al., 2008), glycosomes (Colasante et al., 2006; Güther et al., 2014), flagellum (Broadhead et al., 2006; Subota et al., 2014), and cell surface (Shimogawa et al., 2015) were also used for the prediction of the organelle distribution for the *T. brucei* HSP90 complements and HSP90/HSPC complements and HSP83 co-chaperones.

### 2.5 Identification of potential post-translational modification sites for the *T. brucei* HSP83 proteins

Data mining from a collection of relevant databases on *T. brucei* PTMs (Nett et al., 2009b; Urbaniak et al., 2013; Moretti et al., 2018; Zhang et al., 2020) for the relevant proteins was retrieved using the previously identified accession numbers. Information on the respective PTMs (modification sites, modification types, and modified residue) were obtained, and the modified residues were mapped onto Supplementary Figure S2 for all HSP90 isoforms from *T. brucei* subspecies (*T. b. brucei* and *T. b. gambiense*) with orthologues from other trypanosomatids and from human, then analyzed for determination of conserved and specific PTMs among the *T. brucei* HSP90 complements.

## 3 Results and discussion

### 3.1 Determination of the *T. b. brucei* and *T. b. gambiense* HSP90/HSPC complements

The protozoan parasite *T. brucei* comprises three subspecies, with the genomes of *T. b. gambiense* and *T b. brucei* already sequenced (Jackson et al., 2010; Gibson, 2012). Any information obtained from the genome of the non-human infective *T. brucei* subspecies, *T. b. brucei*, can be inferred for the human infective subspecies, *T. b. rhodesiense*, as the *T. b. brucei* TREU927 strain displays the full range of known *T. brucei* phenotypes and possesses similar biological and genetic characteristics (Gibson, 2012). However, the *T. b. gambiense* genome was sequenced due to the subspecies displaying profoundly different biological and genetic characteristics (Jackson et al., 2010). Genome-wide identification and *in silico* analyses of the HSP90/HSPC complement in both *T. brucei* subspecies was conducted to provide an overview of the *T. brucei* HSP90 family. The ortholog of the cytosolic HSP90 member in *T. brucei* is termed HSP83 (Mottram et al., 1989), while in this study we refer to the ER ortholog as GRP94 and the mitochondrial ortholog as TRAP-1. However, to underscore whether discussing a protein from *T. b. gambiense* or *T. b. brucei*, the abbreviations Tbg and Tbb were used in this study, respectively. The orthologous relationships of the HSP90 family from *T. b. brucei* and *T. b. gambiense* to the selected organisms in this study are presented in Table 1, and a comprehensive domain organization of the predicted *T. brucei* HSP90 proteins is illustrated in Supplementary Figure S3.

Twelve putative *HSP90 genes* were identified to be encoded on the *T. b. brucei* genome (Table 1), which is consistent with previous findings (Mottram et al., 1989; Folgueira and Requena, 2007), while *T. b. gambiense* was identified in this study to only have five putative *HSP90* genes. The reduction in the *HSP90* gene numbers found in *T. b. gambiense* could be a consequence of the reduced genome size observed in the human infective subspecies (Dero et al., 1987). The intraspecific genomic variation is largely associated with tandem or segmental duplications observed in *T b. brucei* (Jackson et al., 2010)*.* This study also identified an unassigned putative *HSP90* gene (Tb11. v5.0543) in the animal infective subspecies, *T b. brucei*, but this sequence could not be assembled into a chromosome and was part of a bin scaffold that was not considered during reannotation efforts. For the putative *HSP90* genes identified in this study for *T b. brucei*, 10 of the 12 putative *HSP90* genes identified were found to be homologous to HSP83, whereas in *T b. gambiense*, three of the five putative *HSP90* genes were homologous to HSP83 (Table 1). The remaining two *HSP90* genes found in both *T b. brucei* (Tb927.3.3580 and Tbg972.3.3850) and *T b. gambiense* (Tb927.11.2650 and Tbg972.11.2900) showed significant identity to the ER and mitochondrial resident paralogues of HSP90, GRP94, and TRAP-1, respectively (Table 1). This indicates that a single-gene copy for GRP94 and TRAP-1 is encoded on the genome in both *T. brucei* subspecies. Phylogenetic analysis shows that the *T. brucei* HSP90/HSPC family also comprises three distinct HSP90 groups (HSP83, GRP94, and TRAP-1), which cluster into clades according to protein sequence and subcellular localization (Supplementary Figure S4).

**FIGURE 1:**
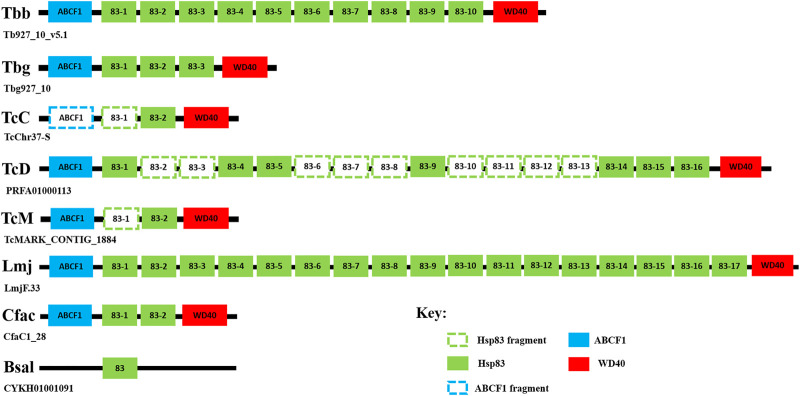
Syntenic analysis of the gene arrangement of the *HSP83* genes in *T. brucei* and selected trypanosomatids. The conserved syntenic regions surrounding the selected *HSP83* genes were searched by examining the conserved co-localization of neighboring genes on chromosome 10 on a scaffold of the *T. brucei* subspecies, *T b. brucei* (Tbb), and *T b. gambiense* (Tbg), and selected trypanosomatids: *T. cruzi* CL Brener Esmeraldo-like (TcC), *T. cruzi* Dm28c 2018 (TcD) strain, *T. cruzi* marinkelli strain B7 (TcM), *L. major* (Lmj), *B. saltans* (Bsal), and *C. fasciculata* (Cfac). The genome information used for this study was acquired from the TriTrypDB database (http://tritrypdb.org/tritrypdb/) (Aslett et al., 2010). The identities of unknown neighbor genes of the selected *HSP83* genes were conducted using a BLASTP search on the NCBI database. Abbreviations: ABCF1: ATP-binding cassette sub-family F member 1; WD40: WD40-repeat protein.

Previous literature reported that 11 *HSP90* genes are encoded on the *Trypanosoma cruzi* (*T. cruzi*) genome (Shonhai et al., 2011). In this study we included three different *T. cruzi* strains: CL Brener Esmeraldo-like (TcCLB), Dm28c 2018 (C4B63), and marinkelli strain B7 (Tc_MARK) to determine the HSP90/HSPC complement in the American trypanosome. It was identified in this study that the *T. cruzi* CL Brener Esmeraldo-like strain has two *HSP90* genes, the Dm28c 2018 strain has nine *HSP90* genes, and the marinkelli strain B7 has three *HSP90* genes (Table 1). However, this study identified that many of the *HSP90* genes homologous to HSP83 in the three *T. cruzi* strains were found to be partial and/or truncated genes. In our syntenic analysis, these partial and/or truncated genes were included as they are probably a result of the methodology utilized to sequence the various *T. cruzi* strains, and it is very likely that the truncated sequences are full-length in the genome (Figure 1). The obvious discrepancy in numbers of genes among the *T. cruzi* strains, and its numerous partial and/or truncated HSP90 sequences has been recently reviewed. This review highlights the difficulties in *T*. *cruzi* genome analyses (Herreros-Cabello et al., 2020); the first genome sequenced that is still widely accepted as the main reference has close to 50% repetitions in its sequence (El-Sayed et al., 2005a; 2005b) and though newer genomes have been sequenced using short-read sequencing methods as in the case of the *T cruzi* marinkelli strain B7, these methods end up causing fragmented chromosomes due to their inability to create a complete chromosome from their short reads technique (Franzén et al., 2012; Herreros-Cabello et al., 2020). Other trypanosomatids included in this study were the non-parasitic *Bodo saltans* (*B. saltans*) (Deschamps et al., 2011) and the insect infecting *Crithidia fasciculata* (*C. fasciculata*) (Wallace, 1966), which were found to have three and four putative *HSP90* genes, respectively (Table 1). Both these trypanosomatids were found to possess genes encoding for all three HSP90 isoforms, though *C. fasciculata* was identified to possess two *HSP83* genes (Table 1).

Early genomic studies suggested that the human genome contained 16 *HSP90* genes (five functional and 11 pseudogenes), which have been categorized, according to the proposed standardized guidelines for HSP nomenclature, into four isoforms under the superfamily name HSPC (Chen et al., 2006; Kampinga et al., 2009). In contrast to the trypanosomatids, humans have two isoforms of HSP90 localized in the cytoplasm: the inducible form HSP90α/HSPC2 and the constitutive form HSP90β/HSPC3 (Subbarao Sreedhar et al., 2004). Phylogenetic analysis has suggested that the two cytosolic isoforms arose from gene duplication, and the organelle HSP90s (GRP94/HSPC4 and TRAP-1/HSPC5) developed from a common ancestor (Gupta, 1995; Emelyanov, 2002; Chen et al., 2005).

### 3.2 HSP83

The ortholog of the cytosolic HSP90 member in trypanosomatids as mentioned previously is commonly referred to as HSP83 and has been found to be an essential and highly abundant protein that is encoded by multiple gene copies organized in a head-to-tail tandem array (Folgueira and Requena, 2007). It has been identified in this study and previous studies (Mottram et al., 1989; Folgueira and Requena, 2007) that *T b. brucei* has been shown to encode for 10 tandem copies of *HSP83* (Figure 1), whereas *T b. gambiense* genome encodes for three tandem copies of *HSP83* (Figure 1). Syntenic analysis revealed that the *TbbHSP83* and *TbgHSP83* genes are both located on chromosome 10 in a head-to-tail orientation, with the same genomic organization being observed in both *T. brucei* subspecies (Figure 1). A discrepancy in *HSP83* gene copy numbers was observed for the three *T. cruzi* strains used in this study (Figure 1). Syntenic analysis revealed that the *T. cruzi* Dm28c 2018 (C4B63) strain has 16 tandem copies of HSP83, though nine were partial sequences (Figure 1), whereas both the CL Brener Esmeraldo-like (TcCLB) and marinkelli strain B7 (Tc_MARK) encode for two *HSP83* genes, with one partial gene each (Figure 1). *Leishmania major* (Lmj) contained the largest HSP90 family with a total of 19 *HSP90* genes (Table 1), 17 tandem copies were found to be homologous to HSP83, and these findings agree with previous studies (Folgueira and Requena, 2007; Shonhai et al., 2011; Requena et al., 2015), also correlating with the high abundance of the protein being observed in *L. major* and several other *Leishmania* spp. (Brandau et al., 1995). Syntenic regions surrounding the *HSP83* genes were found to be virtually conserved across the selected trypanosomatids, with *B. saltans* being the exception (Figure 1). Thus, the discrepancy in gene copy number of *HSP83* in the two *T. brucei* subspecies and among the trypanosomatids may have arisen from the differences in their life cycles.

Data mining of transcriptomic and proteomic datasets revealed that all identified TbbHSP83 (TbbHSP83 1–10) proteins are constitutively expressed at all life cycle stages of the parasite, as well as expressed at all phases of the cell cycle (Gunasekera et al., 2012; Urbaniak et al., 2012). The protein expression of the TbbHSP83 proteins were all reported to be upregulated at the BSF stage (Urbaniak et al., 2012), despite gene regulation being unchanged in both the bloodstream and procyclic life cycle stages (Gunasekera et al., 2012). All TbbHSP83 proteins were also present in the cell surface proteome (Subota et al., 2014), though only TbbHSP83-10 (Tb927.10.10980) was found to be present in the flagellar proteome (Shimogawa et al., 2015).

The amplification of HSP genes in protozoan parasites has been reported previously (Urményi et al., 2014; Requena et al., 2015; Drini et al., 2016; Bentley et al., 2019) and is considered a means by which the parasites increase chaperone levels to maintain proteostasis under normal and stressful conditions (Wiesgigl and Clos, 2001). The heat shock response is a highly conserved transcriptional program that in most organisms involves increased heat shock gene transcription (de Nadal et al., 2011). However, in trypanosomatids, control of gene expression occurs almost exclusively at the post-transcriptional level, and that HSP synthesis during heat shock depends on regulation of mRNA turnover and translational control (Clayton and Shapira, 2007; Requena, 2011). In *T. brucei*, post-transcriptional regulation of chaperone mRNAs is facilitated by a zinc finger protein, ZC3H11 (Droll et al., 2013). The mRNA transcript levels of TbbHSP83 in BSF parasites increases >2-fold after heat shock (Ooi et al., 2020) and is stabilized by ZC3H11 to promote the survival of the parasite (Droll et al., 2013). Treatment of *T b. brucei* BSF parasites with 17-AAG sensitized the parasites to heat shock, as well as caused severe morphological abnormalities and cell cycle disruption (Meyer and Shapiro, 2013). Pharmacological inhibition of HSP83 activity in several *Leishmania* spp. induced morphological and biochemical promastigote-to-amastigote differentiation (Wiesgigl and Clos, 2001; Bente et al., 2003; Hombach et al., 2013), which mimics environmental triggers such as heat shock and acidic milieu, indicating a pivotal role for HSP83 in trypanosomatid protists in environmental sensing and life cycle control. Interestingly, treatment of *T. cruzi* bloodstream trypomastigotes with geldanamycin, induced morphological changes in the parasites but not life cycle progression (Graefe et al., 2002). Therefore, HSP90 cellular homeostasis as a key factor for the control of stage differentiation appears to be dependent on the tropism of the parasite and the different regulatory pathways for life cycle control. It would be interesting to investigate if the pharmacological inhibition of HSP83 affects cellular differentiation among the three *T. brucei* subspecies.

**FIGURE 2:**
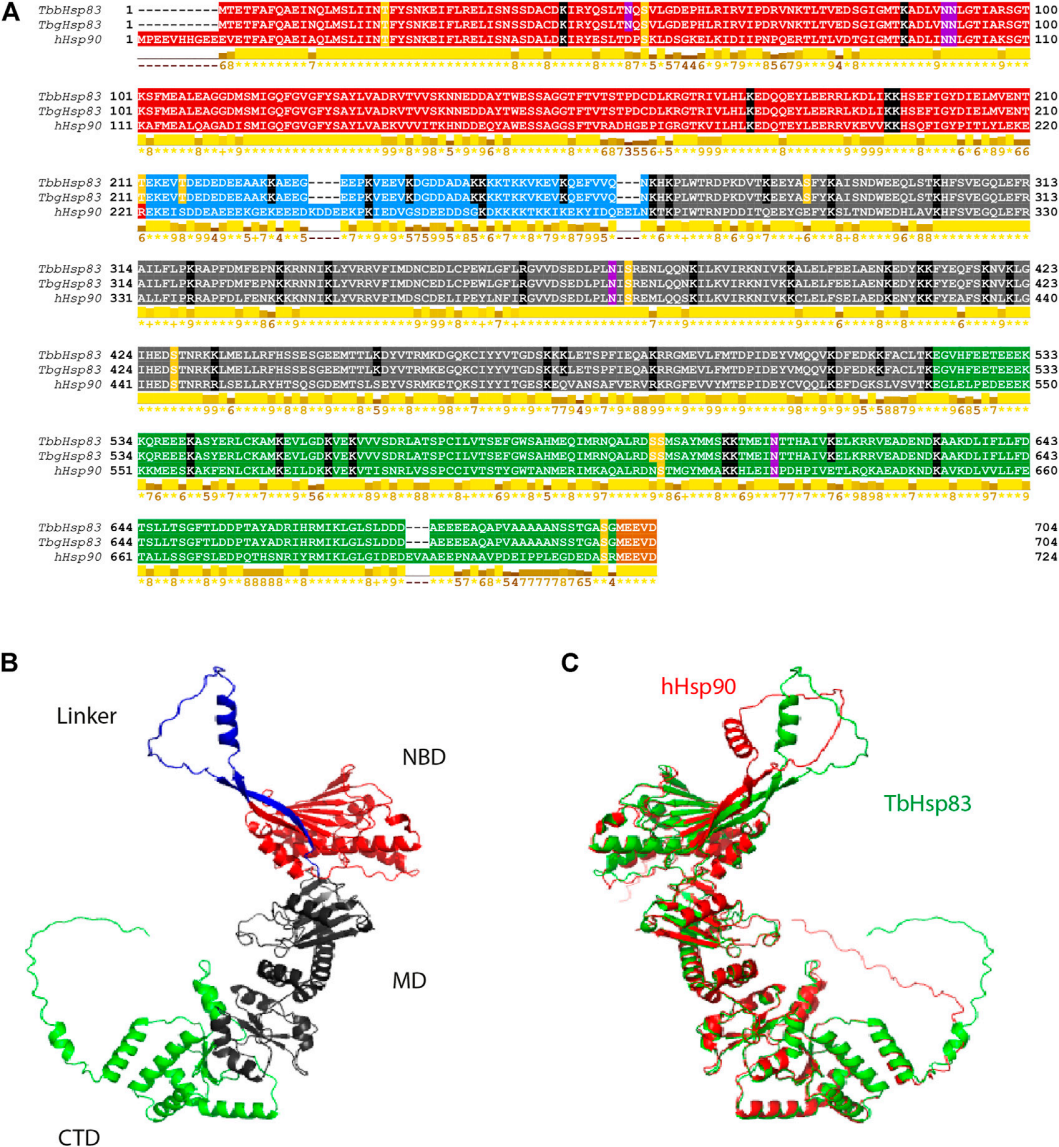
Sequence alignment and 3D structural analysis of TbHSP90. **(A)** Multiple sequence alignment of hHSP90, TbbHSP83, and TbgHSP83. The NTD is highlighted in red, the charged linker domain in blue, the MD in gray, the CTD in green, and the MEEVD motif in orange. The PTMs are highlighted: yellow–phosphorylation, black—acetylation, and pink—N-glycosylation. Conserved residues involved in ATP interaction are highlighted in brown. Conservation based on physico-chemical properties is shown as a numerical index at the bottom of the alignment: “*” denotes score of 11 where amino acid residues are identical; “+” denotes score of 10 and indicates all properties are conserved. **(B)** Predicted 3D structure of the TbHSP83 monomer and **(C)** superimposed 3D structures of TbHSP83 (green) and hHSP90 (red).

The monophyletic cluster of the cytosolic HSP83 proteins suggests a general conservation of function, structure, and sequence in the trypanosomatids HSP83 homologs (Supplementary Figure S4). In the amino acid sequences of TbbHSP83 and TbgHSP83 there was a single substitution at D461 to E in TbgHSP83 (Figure 2A). In comparison, hHSP90 was 63% identical in sequence to TbbHSP83 (Figure 2). The three HSP90 proteins displayed the characteristic domains (Figure 2A): the ATP-binding N-terminal domain (NTD); the middle domain (MD), which plays a role in ATPase activity and is responsible for interacting with client proteins and co-chaperones; and the C-terminal domain (CTD), which is responsible for HSP90 dimerization and interaction with the TPR domain-containing chaperones *via* a C-terminal (MEEVD) motif (Hoter et al., 2018). In addition, the NTD and MD are joined together *via* a charged linker (Jahn et al., 2014). This linker varies in size and is notably shorter in trypanosomatids compared to its human counterpart (Figure 2A) (Silva et al., 2013). Comparison of the hHSP90 to both TbbHSP83 and TbgHSP83 revealed that the amino acid sequence of the NBD was 68% identical, MD 69% identical, and CTD 60% identical (Figure 2). Conversely, the yeast HSP90 proteins (HSP82 and HSC82) were 97% identical in sequence (a difference of 16 amino acid residues) and yet the two proteins exhibit differences in stability, function, and chemical sensitivity (Girstmair et al., 2019). Residues D78 and E32 are conserved in humans and *T. brucei* HSP90 proteins (Figure 2A). Residue D79 (D78 in *T. brucei*) was previously described to be located deep in the inner region of the ATP-binding pocket of yeast HSP90 and determined to form a hydrogen bond with ATP and together with E33 (E32 in *T. brucei*) are important for ATP binding (Panaretou et al., 1998). Mutations of these two residues in yeast HSP90 led to a loss of viability (Panaretou et al., 1998). In comparison to humans, TbHSP83 revealed a 50- to 60-fold higher sensitivity to the HSP90 ATPase inhibitor 17-AAG (Jones et al., 2008). The side chain of residue I171 in TbHSP83 was found to be in contact with L33 and indirectly with I34 (Pizarro et al., 2013), the latter two residues have been implicated in radicicol resistance (Prodromou et al., 2012). Small sequence variations in HSP90 appear to result in large variations in chemical sensitivity between hHSP90 and TbHSP83 (Jones et al., 2008; Prodromou et al., 2012; Pizarro et al., 2013).

The overall 3D structures of the TbHSP83 and human HSP90 are similar (Figure 2B). The RMDS between the human and Tb structures is 1.71 Å for a Cα superposition of the full-length proteins. The regions of sequence variation reveal themselves more evidently in the flexibility of the protein (Pizarro et al., 2013; López et al., 2021). The major differences that can be seen in the 3D structures are in the charged linker domain and the C-terminal domain (Figure 2B). The orientation of both regions differs to that of the human HSP90 protein. Furthermore, the C-terminal domain has an extension that contains a short helical structure present in TbHSP83 that is absent in hHSP90 (residues 678–682, EEEEA). The CTD is responsible for dimerization and interaction with co-chaperones, and this may result in the possibility of unique interactors of the TbHSP83 protein. The spatial differences seen in the linker are a direct result of the differences in the length. The hHSP90 has a linker that is seven residues longer than that of TbHSP83 and has a higher overall negative formal charge (Figure 2A). The variation in length of the linker has been noted across different species, the varying length could affect flexibility, and the varying charge could affect transient domain interactions that exist between the NTD and the linker (Jahn et al., 2014; López et al., 2021).

Post-translational modifications, and particularly phosphorylation of tyrosine, serine, and threonine residues, at multiple sites of cytosolic HSP90 is a well-known chaperone activity modulator mechanism in many organisms (Miyata and Yahara, 1992; Mollapour et al., 2011; Mollapour and Neckers, 2012; Hombach-Barrigah et al., 2019), HSP90 steady-state phosphorylation is species-specific relative to the different cellular environments (Mollapour and Neckers, 2012). Two phosphorylation sites, S53 and S286, were found to be unique to *T. brucei* HSP83 and conserved in all 10 cytosolic HSP83 proteins. The phosphorylation sites T211, T216, and S597 were conserved in all analyzed trypanomastids in this study, while S374 and S698 were found to be conserved in all proteins including humans (Supplementary Figure S2). The same phospho-modified residues were previously described for the cytosolic HSP83 ortholog from *L. donovani* (Hombach-Barrigah et al., 2019). Silencing mutations of *L. donovani* HSP90 T211 and T216 reduced parasite growth, while mutation of S594 reduced growth and infectivity (Hombach-Barrigah et al., 2019). The phosphorylation of *L. donovani* HSP90 at T21 plays a role in the binding of co-chaperones, and mutation of this residue irreversibly inhibited the growth of the promastigote stage (Hombach-Barrigah et al., 2019); however, this residue has not been detected as a *T. brucei* phospho-site (Cunningham et al., 2008; Mollapour et al., 2011; Hombach-Barrigah et al., 2019). The equivalent site in yeast HSP90 (T22) was found to be essential for dimerization and ATPase activity (Cunningham et al., 2008). Acetylation and ubiquitination sites were also predicted and mapped. The predicted N-glycosylation sites, N90, N372, and N612 were conserved in all cytosolic HSP90s analyzed in this study, while N51 was determined to be specific to *T. brucei* HSP83 (Supplementary Figure S2). Two ubiquitination sites identified in *T. brucei* HSP83 as K394 and K560 were found conserved in all analyzed cytosolic HSP90 isoforms in this study (Supplementary Figure S2).

### 3.3 TRAP-1

The mitochondrial isoform of the HSP90/HSPC family was first identified in association with the mammalian tumor necrosis factor 1 (TNF-1) protein, hence termed TRAP-1 (Song et al., 1995). It was promptly suggested as a member of the 90-kDa molecular chaperone family due to strong homology with other HSP90 members (Song et al., 1995). Since then, TRAP-1/HSPC5 orthologues have been identified in a variety of eukaryotic and prokaryotic organisms and was also recently referred to as HSP84 in *T. brucei* (Meyer and Shapiro, 2021). RNAi knockdown of HSP84 showed growth defects and led to loss of kinetoplasts in bloodstream form trypanosomes (Meyer and Shapiro, 2021). Our study identified a single entry for a putative *TRAP-1* gene annotated in the genomes of both *T b. brucei* (Tb927.11.2650) and *T b. gambiense* (Tbg972.11.2900) (Table 1). The selected kinetoplastids in this study were also identified to encode a single copy of TRAP-1 (Table 1), which are consistent with previous studies (Folgueira and Requena, 2007), except for *T. cruzi*, which was previously stated to encode for two TRAP-1 orthologues (Folgueira and Requena, 2007; Shonhai et al., 2011). Phylogenetic analysis indicates a general conservation in trypanosomatid TRAP-1 proteins (Supplementary Figure S4), though little experimental characterization of these genes has been conducted in kinetoplastids. It is predicted that the cellular role of the trypanosomatid TRAP-1 proteins will be orthologous to human TRAP-1, whose major functions is to maintain mitochondrial integrity, modulate mitochondrial metabolism, and protect against mitochondrial apoptosis (Altieri et al., 2012). Furthermore, human TRAP-1 counteracts protein aggregation inside the mitochondria and supports protein folding (Siegelin et al., 2011), leading to healthy, intact mitochondria.

Mammalian TRAP-1 orthologues are localized predominantly in the mitochondrial matrix, where it exists as at least six different protein variants, resulting from splicing patterns, amino acid additions and/or deletions (Cechetto and Gupta, 2000; Felts et al., 2000). The translation of the main TRAP-1 mRNA generates a precursor protein of 704 amino acids that contains a putative 59-amino acid, N-terminal mitochondrial import sequence, which is removed upon organelle import (Felts et al., 2000; Schleiff and Becker, 2011). It was predicted that both TbbTRAP-1 and TbgTRAP-1 localize in the mitochondria, as the proteins possess a positively charged N-terminal leader sequence (Supplementary Figure S1). Proteomic and localization studies confirmed that TbbTRAP-1 localizes to the mitochondria (Panigrahi et al., 2009; Dean et al., 2017), but interestingly the protein is also present in the flagella of *T b. brucei* BSF parasites (Subota et al., 2014) (Table 1). The subcellular distribution of TbbTRAP-1 during the parasite’s life cycle could be related to the shape and functional plasticity of the *T. brucei* single mitochondrion, which undergoes profound alterations to adapt to the different host environments (Osellame et al., 2012). Phenotypic knockdown of TbbTRAP-1 had a detrimental effect on the survival and fitness of the parasite at the procyclic stage of its life cycle and negatively affected parasite differentiation (Alsford et al., 2011). Thus, *T. brucei* TRAP-1 proteins may be an important modulator of mitochondrial bioenergetics at the procyclic stage, as well as play an integral role in parasite pathogenesis.

The phosphorylation sites, S363 and S453, were conserved in the middle domain of TRAP-1 for all trypanosome proteins, while S439 was conserved in TRAP-1 for all proteins studied including humans (Supplementary Figure S2). Several amino acids were identified as potential targets for post-translational modifications in human TRAP-1, yet the phosphorylation mechanism remains to be revealed (Altieri et al., 2012). Acetylation sites found specific for TbbTRAP-1 include K109, K480, and K601 (Supplementary Figure S2). Most of the PTMs of HSP90 and other inferences stated are yet to be verified experimentally.

### 3.4 GRP94

The glucose-regulated 94 kDa protein (GRP94) is a HSP90 family member residing in the lumen of the endoplasmic reticulum (ER) (Argon and Simen, 1999), where it is involved in the maturation of membrane-resident and secreted protein clients (Marzec et al., 2012). *GRP94* is present as a single gene in all metazoa, although the gene is not found in many unicellular organisms such as bacteria, archaea, yeast, and most fungi (Marzec et al., 2012). This study identified a single putative entry for the *GRP94* gene in both *T. brucei* subspecies and the selected trypanosomatids in this study (Table 1). These findings are consistent with previous findings for *T. brucei* and *L. major* (Folgueira and Requena, 2007), though previous reports indicated that *T. cruzi* CL Brener Esmeraldo-like strain encodes three GRP94 orthologues (Folgueira and Requena, 2007; Shonhai et al., 2011). However, this study identified that only one *GRP94* gene in *T. cruzi* CL Brener Esmeraldo-like strain (TcCLB.506,989.190) was found to encode for a full-length sequence. The genome of this *T. cruzi* strain needs to be further investigated to determine if these partial sequences of the *GRP94* genes (TcCLB.506591.4 and TcCLB.503811.10) are due to sequencing errors.

Both TbbGRP94 and TbgGRP94 genes are present on chromosome III and are shown to encode for proteins considerably longer in amino acid sequence when compared to HSP83 (Supplementary Figure S1), which is characteristic of GRP94 protein members (Stechmann and Cavalier-Smith, 2003; Johnson, 2012). GRP94 proteins share structural similarity with cytosolic HSP90 proteins, though the N-terminus contains an ER signal peptide while the C-terminal MEEVD peptide is replaced with the KDEL motif that is required for retention in the ER (Argon and Simen, 1999). Sequence analysis of TbbGRP94 and TbgGRP94 indicates that the GRP94 protein shares domain architecture with typical GRP94 proteins including the possession of an N-terminal ER signal peptide (Supplementary Figure S1). However, a variation in the C-terminal ER retention motif, KDEL, is observed in all the trypanosomatid orthologues of GRP94; AGDL in *Trypanosoma* spp., KEEL in *B. saltans*, and EGDL in *C. fasciculata* and all *Leishmania* spp (Supplementary Figure S1). Transcriptomic and proteomic studies revealed that TbbGRP94 is expressed at all life cycles and throughout the phases of the cell cycle (Supplementary Table S2). Proteomic studies confirm the presence of GRP94 in flagella and cell surface (Subota et al., 2014; Shimogawa et al., 2015).

In trypanosomatids, the first recognized and characterized *GRP94* gene was in *Leishmania infantum* (*L. infantum*). The GRP94 ortholog in *L. infantum* was shown to localize in the ER and share many of the activities of GRP94s of other eukaryotes (Descoteaux, 2002). Unlike GRP94 in mammalian cells, LinGRP94 is not essential for cell viability, and *LinGRP94* mRNA is induced developmentally rather than by canonical GRP94-inducing stresses (Descoteaux, 2002). The protein was highly immunogenic during *Leishmania* infection (Larreta et al., 2000, 2002), and essential for lipophosphoglycan (LPG) assembly (Descoteaux, 2002), an abundant surface glycolipid of *Leishmania* promastigotes that is critical to parasite virulence (Yao et al., 2003). Effectively, the critical role of GRP94 in *Leishmania* appears to be adapted to the synthesis of glycoconjugates and directing the host immune response implicating a pivotal role in parasite virulence (Descoteaux, 2002). Though whether this specialized role is conserved in *T. brucei* and other trypanosomatids will need to be elucidated. The function and cellular roles of TbGRP94 should be explored, given the immunogenic and antigenic properties shown by the *L. infantum* GRP94, as this protein could constitute a valuable molecule for diagnostic purposes, and quite possibly a potential candidate for studies of protective immunogenicity. N-glycosylation sites, N137, N370, and N639, were conserved across all species studies (Supplementary Figure S2). GRP94 phosphorylation sites, S63 and S372, were conserved for all species analyzed, while S625 was conserved in *T. brucei* and *T. cruzi* (Supplementary Figure S2). K472 and K504 acetylation sites were conserved in all the trypanosome species, while K515, K542, R587, and Q646 were unique to *T. brucei* (Supplementary Figure S2).

### 3.5 The *T. brucei* HSP90 co-chaperone system

In all organisms, HSP90 is a dynamic protein that undergoes a conformational cycle that is directionally determined, in large part by ATP binding/hydrolysis, and a cohort of proteins termed co-chaperones (Panaretou et al., 1998; Prodromou, 1999; Johnson and Brown, 2009). The HSP90 co-chaperone system in intracellular protozoan parasites has been explored in previous studies (Seraphim et al., 2013; Figueras et al., 2014). Thus, using the human and trypanosomatid systems, this study analyzed the composition of the *T. brucei* HSP83 co-chaperone system. It was determined in this study that *T. brucei* possesses a similar number of co-chaperones compared to humans (Table 2), with one notable absence being cell division cycle 37 (Cdc37). The absence of a gene encoding for Cdc37 has also been noted in several intracellular protozoan parasites (Chua et al., 2014; Figueras et al., 2014; Tatu and Neckers, 2014; Hombach-Barrigah et al., 2019) and was not evident in 10 out of 19 divergent eukaryotic species examined in a study by Johnson and Brown (2009). Cdc37 is a co-chaperone that has a specialized and indispensable role in the maturation and/or stabilization of a large subset of protein kinases in mammalian cells (Smith and Workman, 2009). The absence of Cdc37 in some species is that clients that are dependent on a specific co-chaperone in one species may not require HSP90 for function in other species, thus the protein kinases in protozoan parasites may have evolved in such a way that the proteins bind a different co-chaperone or are independent of HSP90 for function. Since little is known about why a protein becomes dependent on HSP90 for activity or stability, it poses interesting questions on the mechanism by which the maturation and regulation of protein kinases in protozoan parasite is mediated dependent or independent of HSP83. Exploration of this mechanism may provide a potential avenue for chemotherapeutics since protein kinases are also an attractive drug target in infectious disease, such as African trypanosomiasis. The identified HSP83 co-chaperones in both *T. brucei* subspecies are listed in Table 2. In addition, the HSP90 co-chaperones were categorized in this study based on the presence of a TPR or CS (CHORD and SGT1) domain.

**TABLE 2:** HSP83/HSPC co-chaperones from *Trypanosoma brucei* with their putative orthologues in *T. cruzi*, *L. major*, *C. fasciculata*, *B. saltans*, and *H. sapiens*.

*H. sapiens*	*H. sapiens*	*T. brucei*	*T. cruzi* ^3^	*L. major*	*C. fasciculata*	*B. saltans*		
Name	Gene ID^1^	Gene ID^1^	Gene ID^1^	Gene ID^1^	Gene ID^1^	Gene ID^1^	Localization^2^	Reference
**A: TPR-containing Hsp90 co-chaperones**								
STi1/HOP	10963	Tb927.5.2940	Tc_MARK_9009	LmjF08.1110	CFAC1_020023900	BSAL_57725	CYTO	Gunasekera et al. (2012); Urbaniak et al. (2012); Butter et al. (2013); Shimogawa et al. (2015); Dean et al. (2017)
							NUC	
		Tbg972.5.4130	C4B63_59g115				CELL SURFACE (BSF, PF)	
PP5	5536	Tb927.10.13670 Tbg972.10.16800	TcCLB.507.993.190	LmjF.18.0150	CFAC1_140007400	BSAL_15705	CYTO (BSF, PF)	Gunasekera et al. (2012); Urbaniak et al. (2012); Butter et al. (2013); Dean et al. (2017)
			C4B63_4g368					
Cyp40	5481	Tb927.9.9780 Tbg972.9.5630	TcCLB.506,885.400 Tc_MARK_4311	LmjF.35.4770	CFAC1_300099000	BSAL_06490	CYTO	Oberholzer et al. (2011); Dean et al. (2017)
			C4B63_2g294				FLAGELLAR (BSF)	
DnaJC7	7266	Tb927.10.4900	TcCLB.504.203.60	LmjF.36.0500	CFAC1_250012000	BSAL_30720	CYTO	Urbaniak et al. (2012); Butter et al. (2013); Dean et al. (2017)
			Tc_MARK_8493					
		Tbg972.10.5950	C4B63_13g112				NUC (BSF, PF)	
FKBP5	2289	Tb927.10.16100 Tbg972.10.19710	TcCLB.511.353.10 Tc_MARK_4665	LmjF.19.1530	CFAC1_210025000	BSAL_03610 BSAL_65235	CYTO	Gunasekera et al. (2012); Urbaniak et al. (2012); Butter et al. (2013); Subota et al. (2014); Shimogawa et al. (2015); Dean et al. (2017)
			C4B63_157g28 C4B63_171g30				FLAGELLAR (BSF, PF)	
SGT	6449	Tb927.6.4000 Tbg972.6.3780	TcCLB.511.737.10 Tc_MARK_2022	LmjF.30.2740	CFAC1_260051600	BSAL_66445	CYTO	Gunasekera et al. (2012); Urbaniak et al. (2012); Butter et al. (2013); Subota et al. (2014); Shimogawa et al. (2015); Dean et al. (2017)
							FLAGELLAR	
			C4B63_18g260				CELL SURFACE (BSF, PF)	
TPR domain protein	7268	Tb927.10.11380	TcCLB.507.709.70	LmjF.33.0700	CFAC1_280016100		CYTO	Urbaniak et al. (2012); Butter et al. (2013); Dean et al. (2017)
		Tbg972.10.13740	Tc_MARK_3,620					
			C4B63_84g34					
**B: CS-containing Hsp90 co-chaperones**								
p23	10728	Tb927.9.10230 Tb927.10.2620 Tbg972.9.5930 Tbg972.10.3260	TcCLB.509.551.70 TcCLB.506.407.60	LmjF.35.4470 LmjF.34.0210	CFAC1_300096200 CFAC1_290030000	BSAL_38665	CYTO	Dean et al. (2017)	
							FLAGELLAR		
			C4B63_2g235 C4B63_47g40				NUC		
CHORD	26548	Tb927.1.3170	TcCLB.507.837.40	LmjF.20.1620	CFAC1_170031900	BSAL_00100	CYTO (BSF, PF)	Gunasekera et al. (2012); Urbaniak et al. (2012); Butter et al. (2013); Dean et al. (2017)	
			Tc_MARK_7,282						
		Tbg972.1.1930	C4B63_93g75						
Aarsd1	80755	Tb927.9.6650	TcCLB.504.883.50	LmjF.15.0690	CFAC1_240038900	BSAL_93700	CYTO	Dean et al. (2017)	
			Tc_MARK_5844						
		Tbg972.9.3510	C4B63_136g36						
PIH1D1	55011	Tb927.9.10490 Tbg972.9.6100	TcCLB.506.147.150	LmjF.35.4330	CFAC1_300094700		CYTO	Dean et al. (2017)	
			Tc_MARK_4354						
			C4B63_80g31						
PIH1D3	139212	Tb927.3.4410 Tbg972.3.4850	TcCLB.507.257.160	LmjF.29.1850	CFAC1_190042200	BSAL_07820	CYTO (BSF, PF)	Urbaniak et al. (2012); Butter et al. (2013); Dean et al. (2017)	
			Tc_MARK_3,141						
			C4B63_10g277			BSAL_56185			
Ubiquitin hydrolase	10869	Tb927.9.13430 Tbg972.9.8400	TcCLB.510.749.40	LmjF.35.2410	CFAC1_300073100	BSAL_27670	CYTO	Dean et al. (2017)	
			Tc_MARK_4527						
			C4B63_2g83						
CS domain/TPR repeat	161582	Tb927.11.16050 Tbg972.11.17990	TcCLB.509.073.90	LmjF.32.2850	CFAC1_190042200	BSAL_62790	CYTO	Dean et al. (2017)	
			Tc_MARK_3396						
			C4B63_2g215						
NADH-cytochrome B5 reductase	51167	Tb927.11.3750 Tbg972.11.4290	TcCLB.511.047.40	LmjF.13.1060	CFAC1_220039400	BSAL_93355	CYTO	Dean et al. (2017)	
			Tc_MARK_6965						
			C4B63_46g107						
LRRC	23639	Tb927.3.4770 Tbg972.3.5330	TcCLB.503.671.30	LmjF.29.2210	CFAC1_200030000	BSAL_68670	CYTO	Butter et al. (2013); Dean et al. (2017)	
			Tc_MARK_3,178						
			C4B63_23g85						
**C: Non-TPR, non-CS-containing Hsp90 co-chaperones**								
Aha1	10598	Tb927.10.13710 Tbg972.10.16840	TcCLB.507.993.150	LmjF.18.0210	CFAC1_140008400	BSAL_15670	CYTO	Gunasekera et al. (2012); Urbaniak et al. (2012); Butter et al. (2013); Dean et al. (2017)	
			Tc_MARK_4860						
			C4B63_4g357				NUC (BSF, PF)		
Phosphoducin	79031	Tb927.11.4930	TcCLB.507.669.130	LmjF.24.0170	CFAC1_21006700	BSAL_83285	CYTO	Dean et al. (2017)	
			Tc_MARK_6540						
		Tbg972.11.5550	C4B63_28g45						

*a* The Gene IDs for the *T b. brucei* (Tb refers to Tbb), *T b. gambiense*, *T. cruzi*, *C. fasciculata, B. saltans*, and *L. major* HSP83/HSPC co-chaperones were retrieved from the TriTrypDB database (http://tritrypdb.org/tritrypdb/; Aslett et al., 2010). The Gene IDs for the members of the *H. sapiens* HSP90/HSPC co-chaperones were retrieved from NCBI (https://www.ncbi.nlm.nih.gov/).

b The Gene IDs, for the orthologues, identified by reciprocal BLASTP analysis, of three strains of *T. cruzi* are listed. *T. cruzi* CL, Brener Esmeraldo-like (TcCLB), *T. cruzi* Dm28c 2018 (C4B63), and *T. cruzi* marinkelli strain B7 (Tc_MARK).

c Subcellular localizations for the *T. brucei* HSP83/HSPC co-chaperone proteins were acquired from using the TrypTag database (http://tryptag.org/; Dean et al., 2017) and/or determined using various proteomic datasets listed in the materials and methods.

CYT-Cytosol; MITO- mitochondrion; NUC- nucleus; ER- endoplasmic reticulum; GYLCO-glycosomes; FLAGELLAR- flagellar; CELL SURFACE- cell surface.

### 3.6 TPR-containing HSP83 co-chaperones

Seven putative TPR-containing co-chaperones were identified in this study.

#### 3.6.1 Stress-inducible protein 1 (STI1)

Stress-inducible protein 1 (STI1), also known as HSP70/HSP90-organizing protein (HOP or STIP1) in mammals, is one of the best studied co-chaperones in the HSP90 reaction cycle (Chang et al., 1997; Johnson et al., 1998) as it acts as an adapter protein, mediating the interaction between HSP70 and HSP90 through its TPR domains (Brinker et al., 2002; Odunuga et al., 2003; Baindur-Hudson et al., 2015). STI1/HOP is a widely conserved HSP90 co-chaperone and has been annotated and characterized across diverse organisms including several kinetoplastid protists. Initially thought to be an indispensable protein, recent discoveries in yeast and some eukaryotes show that direct interaction can take place *in vitro* between HSP70 and HSP90 in the absence of HOP (Kravats et al., 2018; Bhattacharya et al., 2020). A single *STI1/HOP* gene was found encoded in both *T. brucei* subspecies (Table 2). Nine TPR motifs arranged into three TPR domains (TPR1, TPR2A, and TPR2B) in addition to two domains rich in proline and aspartic acid (DP1 and DP2) were predicted (Scheufler et al., 2000; Nelson et al., 2003). Both STI1/HOP orthologues in *T. cruzi* and *L. major* were found to immunoprecipitate with HSP83 and HSP70 and co-localize with these chaperones in the cytoplasm and/or around nucleus (Webb et al., 1997; Schmidt et al., 2011). The expression of HOP isoforms was increased in response to different environmental stresses (Webb et al., 1997; Schmidt et al., 2011), with LmjHOP being upregulated when the parasites are exposed under heat stress conditions (Webb et al., 1997), whereas only nutritional stress-induced expression of TcSTI1 in the late growth phase of epimastigotes (Schmidt et al., 2011). The HSP90-STI1 complex in *L. major* and *T. cruzi* has been shown to be pivotal to parasite differentiation (Webb et al., 1997; Hombach et al., 2013). Proteomic analysis in *T. brucei* indicates that TbbSTI1 is part of the cell surface (PF) proteome during the procyclic stage (Shimogawa et al., 2015). Though TbbSti1 is present in both the BSF and PF stages of the parasite, it was more highly expressed in the bloodstream form (Gunasekera et al., 2012; Urbaniak et al., 2012; Butter et al., 2013). These data suggest that the STI1 ortholog in both *T. brucei* subspecies should function as an adapter protein for TbHSP83 and TbHSP70s, participating in the foldosome apparatus necessary for maintaining proteostasis, cytoprotection, and modulating parasite differentiation.

#### 3.6.2 Protein phosphatase 5 (PP5)

Protein phosphatase 5 (PP5) is a member of the PPP family of serine/threonine protein phosphatases and it associates with HSP90 in complexes during client protein maturation (Cohen, 1997; Chinkers, 2001; Golden et al., 2008). PP5 is characteristically unique from other PPP family members, in which it possesses an N-terminal TPR domain (Borthwick et al., 2001), which mediates interaction with HSP90 (Chen et al., 1996). This interaction enables PP5 to modify the phosphorylation status of HSP90 client proteins (Golden et al., 2008). The gene for PP5 in *T b. brucei* (TbbPP5) has been extensively studied. TbbPP5 encodes a ∼52-kDa protein that possesses the canonical N-terminal TPR domain and phosphatase catalytic domain (Anderson et al., 2006). TbbPP5 interacted with TbbHSP90 *in vivo* and co-localized with the chaperone in the cytosol of PRO parasites (Jones et al., 2008). Both TbbPP5 and TbbHSP90, upon heat shock and geldanamycin treatment, accumulated in the nucleus (Jones et al., 2008), indicating that both TbbPP5 and TbbHSP90 translocate to the nucleus when the parasites are exposed to proteotoxic stresses (Jones et al., 2008). TbbPP5 was detected in both BSF and PF stages of the parasite but upregulated in the procyclic form (Gunasekera et al., 2012; Urbaniak et al., 2012; Butter et al., 2013). Overexpression of TbbPP5 was found to partially negate the effect of geldanamycin treatment on cell growth, which indicates that the co-chaperone enhances the chaperoning function of TbbHSP90 and promotes the folding and maturation process of important regulatory molecules, which facilitate cell growth.

#### 3.6.3 Peptidyl-prolyl cis–trans-isomerases (PPIases)

The immunophilin superfamily consists of highly conserved proteins with rotamase or peptidyl-prolyl cis–trans-isomerase (PPIase) activity that accelerates protein folding by mediating the isomerization of X-Pro-peptide bonds (Galat, 2003; Pratt et al., 2004). The best characterized PPIases belong to two families, the cyclophilin-type (Cyp) and the FKB-506 drug-binding protein type (FKBP) (Steiner and Haughey, 2010). Data mining of the *T. brucei* genome identified that Cyp40 and a putative FKB-506 binding like protein (FKBPL) are present in the extracellular parasite proteome (Table 2). Investigation of the domain structure and sequence conservation indicate that both Cyp40 and FKBPL in *T. brucei* were shown to display the characteristic two-domain structure of a N-terminal PPIase domain and a C-terminal TPR domain (data not shown). Though it must be noted that the C-terminal TPR domain in kinetoplastid Cyp40 underwent substantial evolutionary modification (Yau et al., 2010), thus potentially impacting Cyp40-HSP83 interactions. Future structure/function studies should explore the effect these modifications have on the isomerase and chaperone activities of the protein in comparison to its human counterpart.

Studies conducted on the Cyp40 ortholog in *L. donovani* have revealed that the protein functions in *Leishmania* stage-specific morphogenesis, motility, and the development of infectious-stage parasites (Yau et al., 2010, 2014). The study conducted by Yau et al. (2014) also suggested that LdCyp40 and LdFKBP2 functions in regulating *Leishmania* cytoskeletal dynamics. Given the capacity of Cyp40 and FKBP52 to compete for molecular partners (Ratajczak et al., 2003), LdCyp40 may interact with microtubules to promote tubulin polymerization as a means of counteracting LdFKBP52-mediated depolymerization. RNAi-mediated knockdown of both Cyp40 and FKBPL in *T b. brucei* parasites demonstrated that these proteins are essential at the BSF stage and are required for parasite differentiation (Alsford et al., 2011; Gunasekera et al., 2012; Urbaniak et al., 2012; Butter et al., 2013). Proteomic data predicted these proteins to reside in the cytosol and flagellar (Oberholzer et al., 2011; Subota et al., 2014). Together these data indicate that *T. brucei* Cyp40 and FKBPL may play essential roles in morphogenesis, motility, and the development of infectious-stage parasites.

#### 3.6.4 J-protein 52

The J-protein family is a major subset of co-chaperones for the HSP70 chaperone machinery, and they are broadly classified into four subtypes (I–IV). The J-protein family from *T. brucei* has been explored previously (Bentley et al., 2019). It was shown in that study that J52 is one of six type III J proteins in *T. brucei* that possesses the TPR domain (others are J42, J51, J52, J53, J65, and J67) (Bentley et al., 2019). J52 is predicted to reside in the cytosol together with J51 and J42 (Bentley et al., 2019). DnaJC7/Tpr2, the human ortholog of J52 was first identified as a cytosolic protein *via* a two-hybrid screen for interaction with a GAP-related segment (GRD) of neurofibromin. It was reported to encode seven TPR units and possess a domain of high similarity to the DnaJ family (Murthy et al., 1996). DnaJC7 also regulates the multichaperone system involving HSP70 and HSP90 but in a nucleotide-independent manner with HSP90. DnaJC7 is predominantly thought to be involved in retrograde transport of client proteins from HSP90 to HSP70 (Brychzy et al., 2003; Moffatt et al., 2008). Proteomic analysis showed J52 to be upregulated in the procyclic form of the parasite (Urbaniak et al., 2012; Butter et al., 2013).

#### 3.6.5 Small glutamine-rich TPR-containing protein (SGT)

The small glutamine-rich TPR-containing protein (SGT) is a co-chaperone involved in a specific branch of the global cellular quality control network that determines the fate of secretory and membrane proteins that mislocalize to the cytosol (Leznicki and High, 2012; Wunderley et al., 2014). Human SGT is a modular protein characterized by three characteristic sequence motifs, namely, an N-terminal dimerization domain, central TPR domain, and a glutamine-rich region at the C terminus (Roberts et al., 2015). The SGT orthologues in trypanosomatids are atypical (Table 2), as these proteins all lack the characteristic glutamine-rich region and contain a substituted region with charged amino acid residues (Ommen et al., 2010). Proteomic analysis identified TbbSGT to be upregulated in the procyclic form of the parasite and was found in the flagellar and cell surface proteome (Gunasekera et al., 2012; Urbaniak et al., 2012; Butter et al., 2013; Subota et al., 2014; Shimogawa et al., 2015). The SGT ortholog in *L. donovani* is an essential protein for *L. donovani* promastigote growth and viability (Ommen et al., 2010). LdSGT was shown to form large, stable complexes that included HSP83, HSP70, HIP, HOP, J-proteins, and HSP100 (Ommen et al., 2010), whereas recombinant *L. braziliensis* SGT was shown to interact with both LbHSP90 and HsHSP70-1A (Coto et al., 2018). Therefore, the orthologous proteins in *T b. brucei* and *T b. gambiense* may have developed the same activity and assist in the formation of the *T. brucei* HSP83 chaperone system. Though future studies should be conducted to elucidate SGT-HSP70/HSP83 interaction in *T. brucei*.

#### 3.6.6 Tetratricopeptide repeat protein 4 (TTC4)

The co-chaperone TTC4 is the Tetratricopeptide repeat protein 4, a member of the TPR family that has been isolated and characterized in humans and is implicated in the pathogenesis of skin melanomas (Su et al., 1999; Poetsch et al., 2000). An ortholog of TTC4 has been characterized in *Drosophila* (Pit47) with both proteins shown to be nucleoplasmic; both contain three TPR motifs and are abundant in proliferating tissue (Crevel et al., 2008). A putative ortholog of TTC4 was found in *T. brucei* and other organisms in this study except in *B. saltans*. Proteomic analysis identified *T. brucei* TTC4 to be upregulated in the procyclic form of the parasite (Urbaniak et al., 2012; Butter et al., 2013).

### 3.7 CS-containing HSP83 co-chaperones

Nine putative CS-containing co-chaperones were identified in this study.

#### 3.7.1 p23

The co-chaperone p23 is a small acidic protein that binds the HSP90 NBD to stabilize the closed conformation of HSP90, inhibiting ATPase activity and preventing client protein release from the complex (Young, 2000; McLaughlin et al., 2006). In addition to its HSP90 co-chaperone function, p23 has its own chaperoning activity *in vitro* and can suppress the aggregation of denatured proteins (Bose et al., 1996; Freeman et al., 1996). *In silico* analysis of the genomes of both *T. brucei* subspecies revealed that the parasite possesses two evolutionarily divergent p23 orthologues, and subsequently these orthologous proteins were named p23a and p23b (Table 2). The possession of two putative p23 proteins was found to be conserved in all the trypanosomatids in this study except *B. saltans* (Table 2). The Tbp23a and Tbp23b proteins share 28% identity to each other and share 33 and 26% identity, respectively, to human p23. In addition, RNAi knockdown of these proteins showed that each p23 protein is essential to parasite viability at specific stages of the life cycle (Alsford et al., 2011). The orthologues of these proteins have been explored in two *Leishmania* spp. (Batista et al., 2015). Both proteins in *L. braziliensis* possessed intrinsic chaperone activity, but they have different client protein specificities; they also inhibit LbrHSP83 ATPase activity to different extents (Batista et al., 2015). Such functional differences might be important in both HSP90 regulation and in their interactions with client proteins during the life stage transformations of kinetoplastid parasites. However, to support these assertions, more functional and *in vivo* studies of trypanosomatid p23a and p23b proteins are needed.

#### 3.7.2 The cysteine- and histidine-rich domain-containing protein (CHORD)

The cysteine- and histidine-rich domain-containing protein (CHORD) is characterized by six cysteine and three histidine residues as well as a C-terminal CS domain as the characteristic domains of the CHORD-containing proteins (Wu et al., 2005). In humans there are two CHORD domains, CHORD-I was found to be dispensable toward the HSP90 interaction while CHORD-II is essential (Wu et al., 2005). CHORD was identified as an ADP-dependent HSP90 co-chaperone in humans as its interaction was shown to be stimulated by high ADP:ATP ratio in cell culture lysates (Gano and Simon, 2010). Data mining identified a single *CHORD* gene in *T. brucei* genome, and all other organisms were studied, and the CHORD protein was found upregulated in procyclic form of *T. brucei* parasite (Gunasekera et al., 2012; Urbaniak et al., 2012; Butter et al., 2013)*.*

#### 3.7.3 Alanyl-tRNA synthetase domain-containing 1 name (Aarsd1)

The mammalian *Aarsd1* gene is a complex gene with large number of exons. The gene gained its name—alanyl-tRNA synthetase domain-containing 1 name (*Aarsd1*) from the shared homology of its 3′ exons to the editing domain of tRNA synthetases (Echeverría et al., 2016). As a co-chaperone with 44% identity to p23 in its CS domain, it is primarily expressed in the heart and skeletal issues and competes with p23 for binding to HSP90 (Taipale et al., 2014; Echeverría et al., 2016). Aarsd1 has previously been identified in *T. brucei* with its involvement in preventin*g* misaminoacylation (Beebe, 2003; Cestari et al., 2013). Data mining revealed a single *Aarsd1* gene in all the organisms studied.

#### 3.7.4 Protein interacting with HSP90 domain-containing protein 1 (PIH1D1/PIH1)

Protein interacting with HSP90 domain-containing protein 1 (PIH1D1/PIH1) also called Nop17 (Zhao et al., 2008) is involved in pre-RNA processing (Gonzales et al., 2005) and functions as an adapter protein that aids in recruiting clients (Henri et al., 2018). PIH1s a component of the R2TP (RUVBL1-RUVBL2-RPAP3-PIH1D1) complex, which has been found to be conserved in many species including yeast and humans (Henri et al., 2018; Martino et al., 2018). The human PIH1 contains an N-terminal domain with which it binds phosphorylated substrates and a C-terminal CS domain to bind other substrates of the R2TP complex (Hořejší et al., 2014). Data mining revealed the ortholog of PIH1 in *T. cruzi* and *L. major* and is the putative pre-RNA processing protein/Nop17 but the ortholog in *T. brucei* is alternatively named a component of motile flagella 56 (CMF56). This protein is absent in *B. saltans.*

#### 3.7.5 PIH1D3

PIH1D3 in humans participates in axonemal dynein assembly in the testis and the respiratory system and mutations in *PIH1D3* have been shown to be a prominent cause of primary ciliary dyskinesia (Olcese et al., 2017). The ortholog of PIH1D3 in all organisms studied is the pre-RNA processing protein/NOP17. Proteomic analysis showed the presence of the PIH1D3 protein in both the bloodstream and procyclic forms of the parasite.

#### 3.7.6 NADH cytochrome B5 reductase 4 (Ncb5or)

Ncb5or is a soluble flavohemoprotein with an N-terminal cytochrome b5-like domain and a C-terminal cytochrome b5 reductase domain (Zhu et al., 2004); it is present in a wide range of tissues in humans including some cancerous cell lines and supposedly functions as an oxygen sensor (Zhu et al., 1999). It contains the CS motif similar to p23 with which it mediates protein–protein interactions (Garcia-Ranea et al., 2002). Orthologues of Ncb5or are present in all organisms studied.

#### 3.7.7 Leucine-rich repeat containing protein (LRRC)

Leucine-rich repeat containing proteins in eukaryotes share functional links with the co-chaperone SGT and together they are involved in the HSP90 chaperone machinery complex activation (Stuttmann et al., 2008). An ortholog of the LRRC protein was found in all organisms studied and it was upregulated in the procyclic form of *T. brucei* parasites (Butter et al., 2013).

#### 3.7.8 Ubiquitin carboxyl-terminal hydrolase (Usp)

Ubiquitin carboxyl-terminal hydrolase 19 (Usp19) in humans has been implicated in various cancers and as a prognostic biomarker in renal cell carcinoma therapy (Shahriyari et al., 2019; Hu et al., 2020). A putative ortholog for ubiquitin carboxyl-terminal hydrolase was found in all organisms studied.

#### 3.7.9 Dyslexia susceptibility 1 candidate gene 1 protein (DYX1C1)

Dyslexia susceptibility 1 candidate gene 1 protein (DYX1C1) in humans has been characterized to possess three TPR domains and is expressed in many tissues including the brain (Taipale et al., 2003). The ortholog in *T. brucei* is a putative CS domain/TPR repeat protein.

### 3.8 Non-TPR, non-CS-containing HSP83 co-chaperones

#### 3.8.1 Activator of HSP90 ATPase homolog 1 (Aha1)

Aha1 has been identified as the primary activator of the ATPase activity of HSP90 and it acts independently of the other co-chaperones. Homologs of Aha1 have been identified across species from yeast to mammals; Aha1 binds with both its N- and C-terminal domain to the NBD and MD of HSP90 to facilitate the dimerization of the chaperone (Mayer et al., 2002; Koulov et al., 2010; Retzlaff et al., 2010). Data mining of the *T. brucei* genome identified that the parasite encodes for a single *Aha1* gene (Table 2). The Aha1 ortholog in *L. braziliensis* (LbrAha1) has been characterized, where it was shown to be a cognate protein that shared several structural and functional properties with the human and yeast orthologues. This suggested similar functional mechanism among these proteins despite the low degree of conservation in the amino acid sequence (Seraphim et al., 2013). Recombinant LbrAha1 stimulated the weak ATPase activity of recombinant LbrHSP83 by around 10-fold, exhibiting a cooperative behavior according to the model that two LbrAha1 molecules can act on one LbHSP83 dimer (Seraphim et al., 2013). Data from proteomic analysis in *T. brucei* revealed that TbbAha1 is upregulated in the BSF stage of the parasite (Gunasekera et al., 2012; Urbaniak et al., 2012; Butter et al., 2013) as well as being essential to parasite viability at this stage of life cycle (Alsford et al., 2011).

#### 3.8.2 Phosphoducin (Pdc)

Phosphoducins in eukaryotes and other members of the phosphoducin family have been shown to function as chaperones/co-chaperones in the G-protein coupled receptors signal transduction pathways (Savage et al., 2000; Willardson and Howlett, 2007). Data mining revealed an ortholog of phosphoducin was found in all organisms studied.

## 4 Conclusion

The HSP90 family contains an abundant and essential group of proteins, which are highly conserved and implicated in a myriad of cellular functions. Due to their role in cellular proteostasis, they have been implicated in the pathology of many diseases which warrants their targeting as therapeutics (Samant et al., 2012)*.* In this article, we report an *in silico* overview of HSP90 and its co-chaperones in both *T. b. brucei* and *T. b. gambiense* in relation to human and other trypanosomal species, including non-parasitic *Bodo saltans* and the insect infecting *Crithidia fasciculata*. *T. b. brucei* was found to have 12 putative HSP90 proteins, 10 of which are cytosolic (HSP83). Multiple copies of HSP83 may allow the parasite to reach a high synthesis level of the proteins in an organism that relies on post-transcriptional regulation, and this explains its high levels in the cell even under non-stress conditions (Requena et al., 2015). The expansion of the HSP90 chaperone complement also reiterates its importance in the biology and functioning of these protozoan parasites (Folgueira and Requena, 2007; Shonhai et al., 2011; Urményi et al., 2014). HSP83 was also found in both stages of the parasite but upregulated in the bloodstream form (BSF), this is similar to previous findings of much higher transcripts of HSP83 in bloodstream forms of *T. brucei* reflecting their temperature induced role of differentiation (Ploeg et al., 1985). The upregulation of HSP83 together with the co-chaperone Sti1 in the BSF may be a further indication of their heat inducibility and involvement in cell defiance, as seen in HSP70 (Urményi et al., 2014).

The protein sequence identities between human and *T. brucei* HSP90 proteins was the lowest in the linker and C-terminal domains; furthermore, the 3D structure revealed differences in the secondary structure and orientations of both regions. These differences may result in an altered mechanism of interacting with co-chaperones. This study identified 18 co-chaperones in the *T. brucei* HSP83 chaperone system, which is less than the current number of 50 co-chaperones in the human system, confirming that the HSP90 chaperone machinery is species-specific (Johnson and Brown, 2009; Dean and Johnson, 2021). We predict that additional co-chaperones of *T. brucei* will be uncovered, some of which will be unique to trypanosomes and possibly *T. brucei*; and this will provide an interface for targeting chaperone/co-chaperone interactions as potential drug targets. Many of the recently discovered co-chaperones in humans are linked to human diseases including cancer (Dean and Johnson, 2021), and while orthologues have been found in *T. brucei* their roles remain to be elucidated. Many of these co-chaperones in *T. brucei* need to be further explored. So far, only the cytosolic HSP90 has been shown to require the function of co-chaperones, the other forms of HSP90 function in the absence of co-chaperones (Richter et al., 2007; Masgras et al., 2017). HSP90 partners with co-chaperones in order to maintain homeostasis; however, these partnerships appear to be dictated by the client protein being chaperoned (Radli and Rüdiger, 2017; Sahasrabudhe et al., 2017). A detailed report for clients in HSP90 is still largely absent (Roy et al., 2012). Previous studies have indicated that inhibitors targeting HSP83 have been shown to cure mice of *T. brucei* infection, although the toxicity of inhibitors to HSP90 in higher eukaryotes is attributed to a functional loss of client proteins and possible cell cycle arrest (Meyer and Shapiro, 2013). Most of the identified HSP90 client proteins in mammals are kinases (Taipale et al., 2012). Despite the fact that most clients for *T. brucei* HSP90 have not been identified, over 170 protein kinases (about 30% of the number present in their human host), have been recognized (Parsons et al., 2005; Nett et al., 2009b). In addition to being regulated by co-chaperones, HSP90 is also regulated by various post-translational modifications. Some of these PTM sites have been indicated as potential regulatory sites which affect the binding affinity of inhibitors in PfHSP90 (Pallavi et al., 2010). A number of unique PTM sites were identified in the TbHSP90 proteins and these could be targeted by inhibitors. The *T. brucei* HSP90, its co-chaperone network, post-translational modifications, and its regulatory mechanisms as well as the subtle structural differences compared to human HSP90 all provide a context for a HSP90-targeted therapy in *T. brucei*.

## Data Availability

The original contributions presented in the study are included in the article/Supplementary Material; further inquiries can be directed to the corresponding author.
