# Supplementary material for: In silico analysis of the HSP90 chaperone system from the African trypanosome, Trypanosoma brucei
Source: Front Mol Biosci. 2022 Sep 23;9:947078. doi: 10.3389/fmolb.2022.947078 (PMC9538636; doi:10.3389/fmolb.2022.947078)
Supplement: Supplementary file 1 [file DataSheet2.PDF]

Supplementary Figure S2

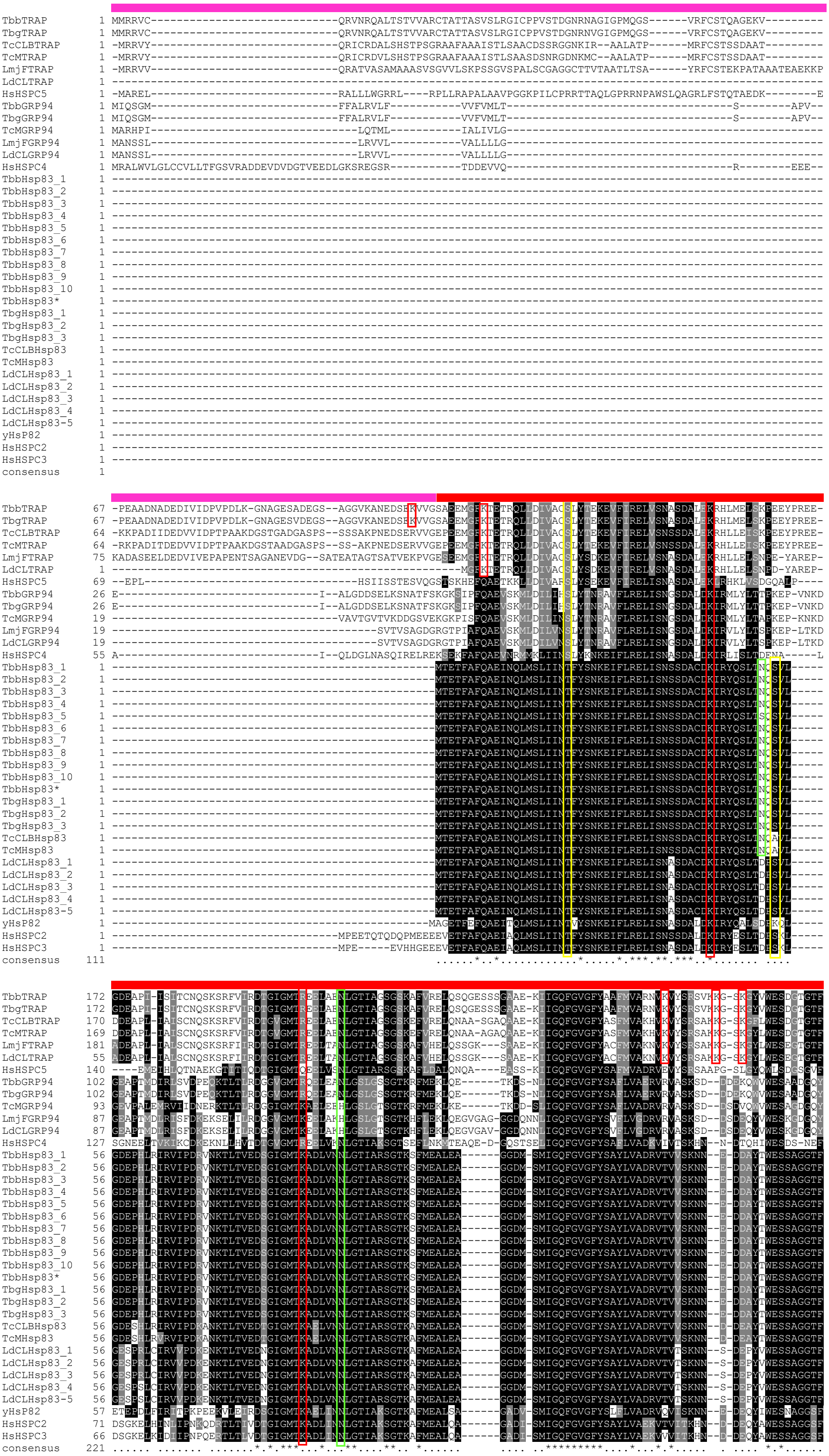

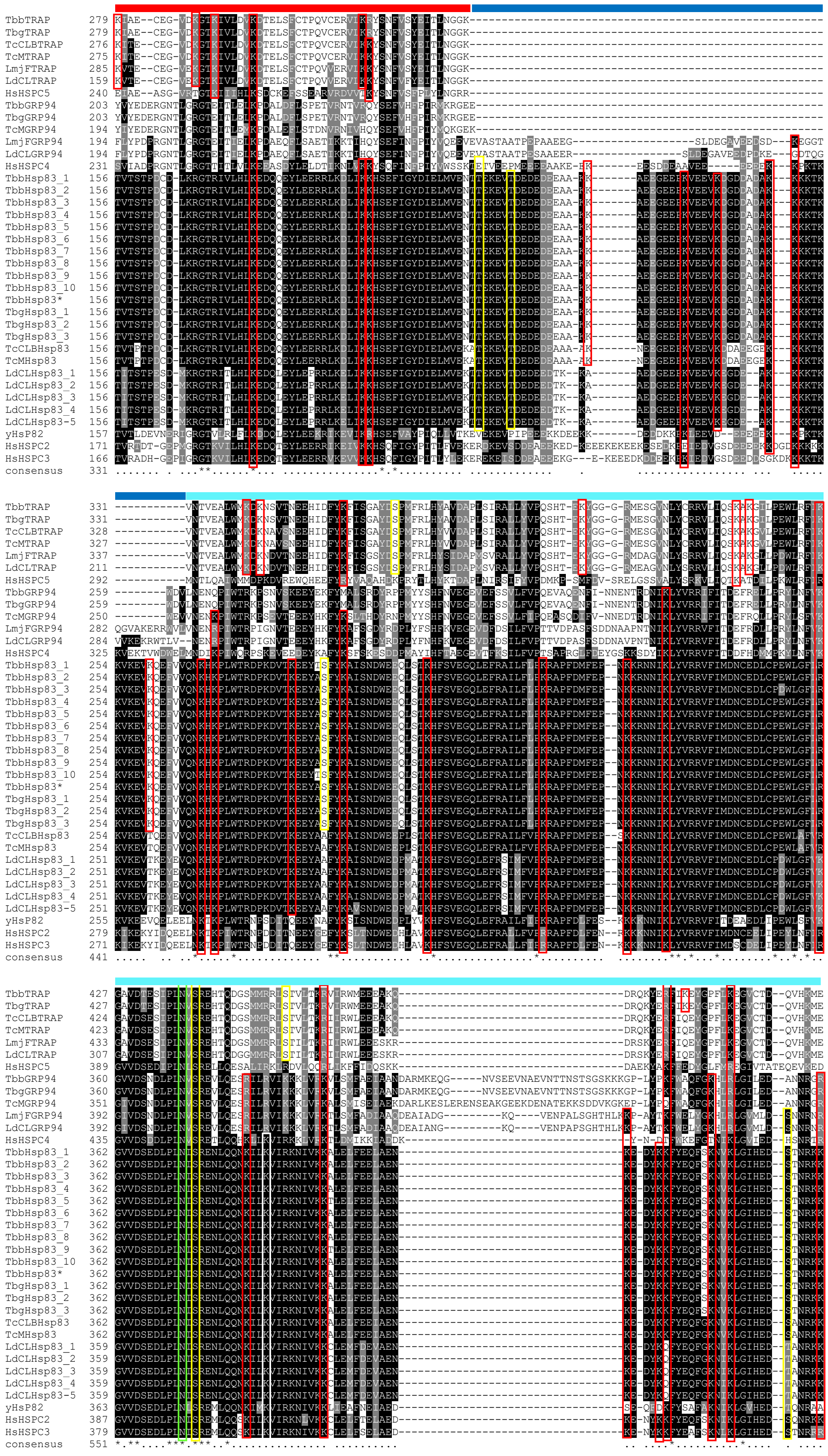

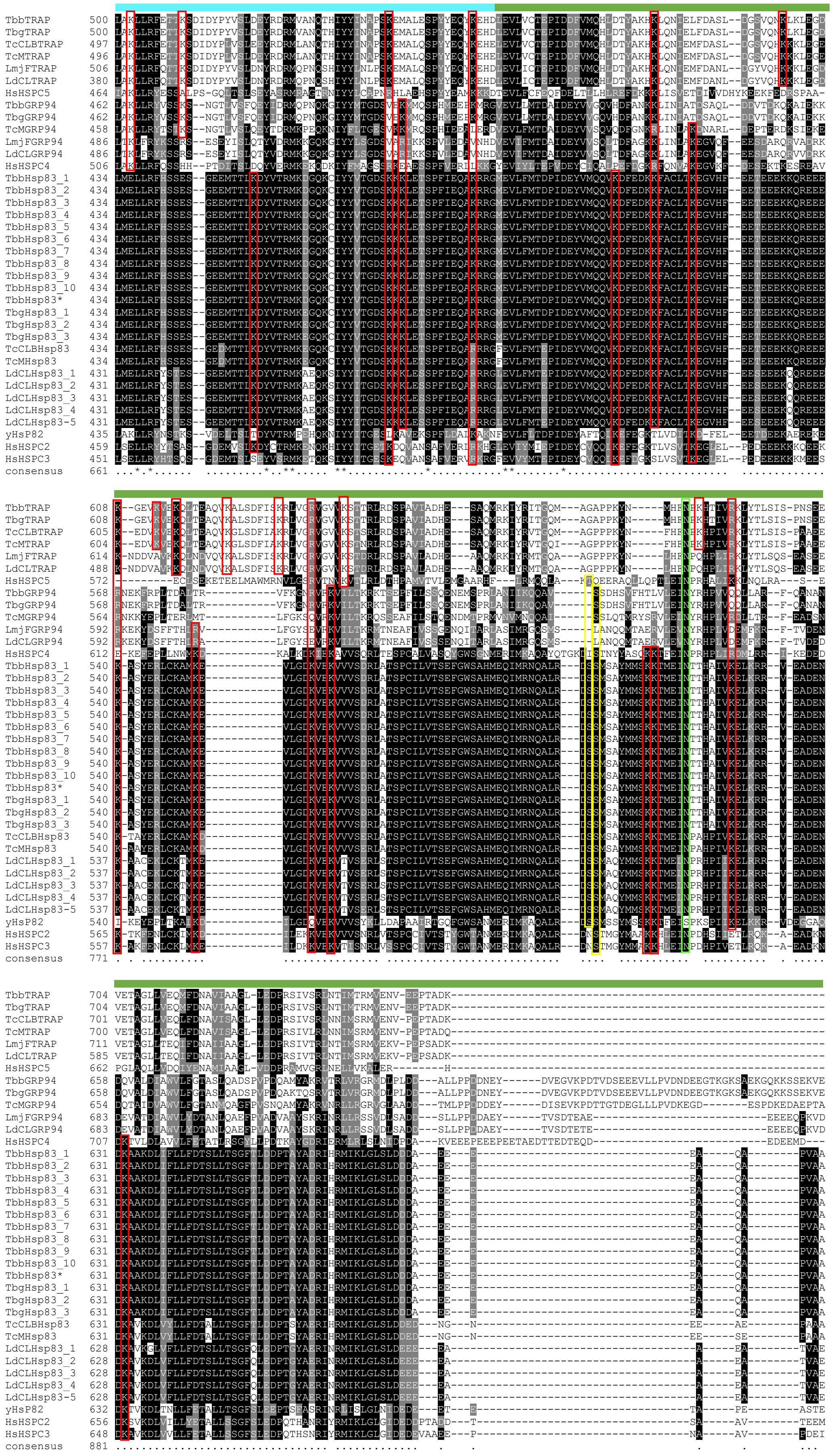

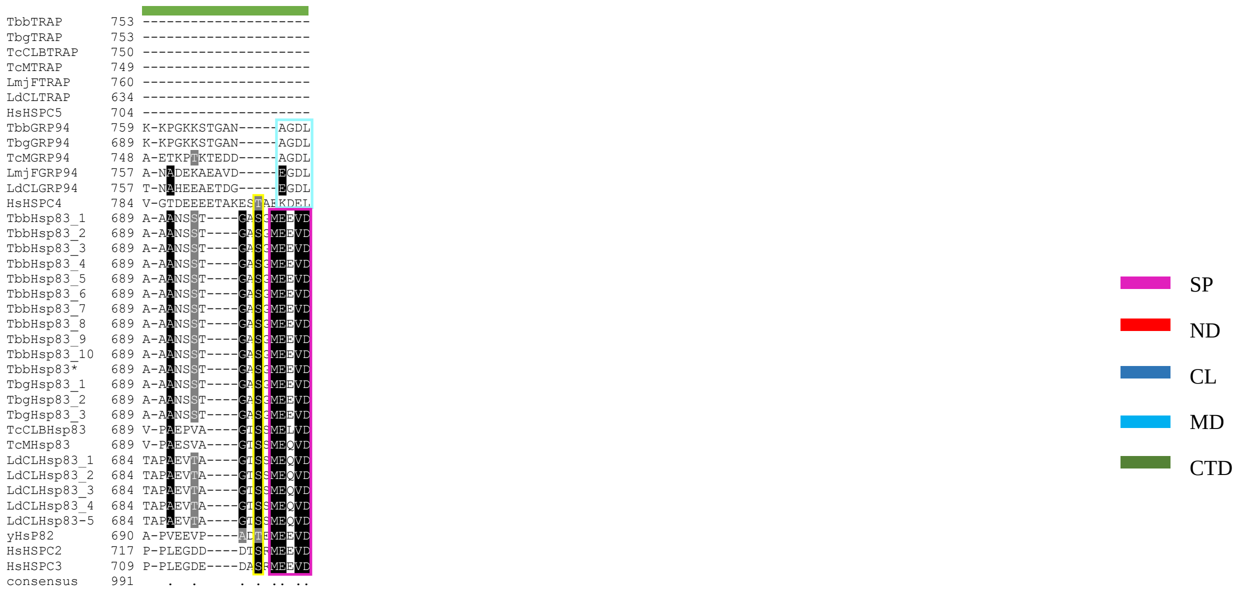

Figure S2: Mapping of the PTMs of the *T. brucei*, humans and selected trypanosomatid HSP90 proteins.

Multiple sequence alignment of the full-length amino acid sequences was performed using the in-built ClustalW program (Larkin et al. 2007) with default parameters in the MEGA7 software (Kumar et al. 2016). Degree of amino acid conservation is symbolized by the following: (*) all fully conserved residues; (:) one of the residues is fully conserved and (.) residues are weakly conserved. The C-terminus motifs are empty-boxed in magenta for the cytosolic HSP90 and light blue for the mitochondrial TRAP-1. Residues involved in post translational modifications accordingly with the MS PTM's proteomic studies by Nett et al, (2009b) and Zhang et al, (2020) Ooi et al, 2020 are colored red for acetylation, green for N-glycosylation and yellow for phosphorylation. The red and yellow empty-boxed are highlighting conserved modified residues. Accession numbers for the Hsp90/HSPC amino acid sequences used in this study are provided in Supplementary Table S1.
