# Supplementary material for: In silico analysis of the HSP90 chaperone system from the African trypanosome, Trypanosoma brucei
Source: Front Mol Biosci. 2022 Sep 23;9:947078. doi: 10.3389/fmolb.2022.947078 (PMC9538636; doi:10.3389/fmolb.2022.947078)
Supplement: Supplementary file 3 [file DataSheet4.DOCX]

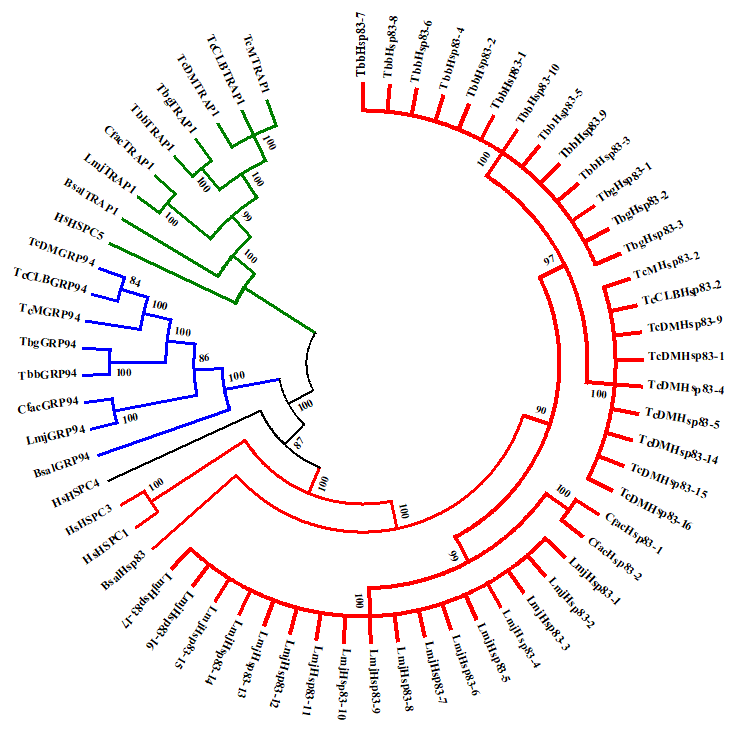


**Figure S4: Phylogenetic analysis of the HSP90/HSPC family from *T. brucei* in relation to human and selected trypanosomatids.** Multiple sequence alignment of the full-length amino acid sequences of the HSP90/HSPC gene families in human and selected kinetoplastid parasites. The multiple sequence alignment provided in Figure S1 was performed using the in-built ClustalW program (Larkin et al., 2007) with default parameters on the MEGA X software (Kumar et al., 2018). The phylogenetic tree was constructed by MEGA 7 using the Maximum-likelihood method based on the Jones–Taylor–Thornton (JTT) matrix-based model of amino acid substitution (Jones et al., 1992) with gamma distribution shape parameter (G). The alignment gaps were excluded from the analysis, and the number of amino acid sites used to construct the tree numbered 572. Bootstrap analysis was computed with 1000 replicates. Accession numbers for the *T. b. brucei* (Tbb), *T. b. gambiense* (Tbg), *T. cruzi* (TcCLB, CL Brener Esmeraldo; TcM, marinkellei strain B7; TcD, Dm28c 2018), *C. fasciculata* (Cf), *B. saltans* (Bs), and *L. major* (Lmj) HSP90 amino acid sequences can be found in Table S1. Accession numbers for human (Hs; *H. sapiens*) and selected trypanosomatid HSPC/HSP90 amino acid sequences are also provided in Table S1. The subcellular localisation for HSP90s is indicated by coloured branches. Red: cytosolic; blue: endoplasmic reticulum; and green: mitochondrion. The numbers on the branches represent the bootstrap values.
