# Supplementary material for: In silico analysis of the HSP90 chaperone system from the African trypanosome, Trypanosoma brucei
Source: Front Mol Biosci. 2022 Sep 23;9:947078. doi: 10.3389/fmolb.2022.947078 (PMC9538636; doi:10.3389/fmolb.2022.947078)
Supplement: Supplementary file 4 [file Table1.DOCX]

| *H. sapiens* | | *T. brucei* | | *T. cruzi* ^3^ | | | *L. major* | | *C. fasciculata* | | *B. saltans* | |
| --- | --- | --- | --- | --- | --- | --- | --- | --- | --- | --- | --- | --- |
| Name | **Ascension ID**^1^ | **Name** | **Ascension ID^2^** | | **Name** | **Ascension ID^2^** | **Name** | **Ascension ID^2^** | **Name** | **Ascension ID^2^** | **Name** | **Ascension ID^2^** |
| Hsp90α  Hsp90β | NP_001017963.2  NP_001258899 .1 | Hsp83-1  Hsp83-1  Hsp83-1  Hsp83-1  Hsp83-1  Hsp83-1  Hsp83-1  Hsp83-1  Hsp83-1  Hsp83-1  Hsp83-1  Hsp83-2  Hsp83-3 | Tb927.10.10890  Tb927.10.10900  Tb927.10.10910  Tb927.10.10920  Tb927.10.10930 Tb927.10.10940  Tb927.10.10950 Tb927.10.10960 Tb927.10.10970 Tb927.10.10980  Tbg972.10.13260 Tbg972.10.13270 Tbg972.10.13280 | Hsp83-2  Hsp83-1  Hsp83-4  Hsp83-5  Hsp83-9  Hsp83-14  Hsp83-15  Hsp83-16  Hsp83-2 | | TcCLB.507713.30  C4B63_113g25  C4B63_113g29  C4B63_113g30 C4B63_113g33 C4B63_84g87 C4B63_84g88 C4B63_84g89  Tc_MARK_3581 | Hsp83-1  Hsp83-2  Hsp83-3  Hsp83-4  Hsp83-5  Hsp83-6  Hsp83-7  Hsp83-8  Hsp83-9  Hsp83-10  Hsp83-11  Hsp83-12  Hsp83-13  Hsp83-14  Hsp83-15  Hsp83-16  Hsp83-17 | LmjF.33.0312 LmjF.33.0314 LmjF.33.0316 LmjF.33.0318 LmjF.33.0320 LmjF.33.0323 LmjF.33.0326 LmjF.33.0330 LmjF.33.0333 LmjF.33.0336 LmjF.33.0340 LmjF.33.0343  LmjF.33.0346 LmjF.33.0350 LmjF.33.0355 LmjF.33.0360 LmjF.33.0365 | Hsp83-1  Hsp83-2 | CFAC1_80011900 CFAC1_280012000 | Hsp83 | BSAL_87515 |
| GRP94 | NP_003290.1 | GRP94  GRP94 | Tb927.3.3580  Tbg972.3.3850 | GRP94  GRP94 | | C4B63_10g439  Tc_MARK_3058 | GRP94 | LmjF.29.0760 | GRP94 | CFAC1_100018800 | GRP94 | BSAL_88715 |
| TRAP-1 | NP_057376.2 | TRAP-1  TRAP-1 | Tb927.11.2650  Tbg972.11.2900 | TRAP-1  TRAP-1  TRAP-1 | | TcCLB.504153.310  C4B63_2g430  Tc_MARK_6238 | TRAP-1 | LmjF33.2390 | TRAP-1 | CFAC1_230028300 | TRAP-1 | BSAL_33145 |

**Table S1**. **Accession numbers for the Hsp90/HSPC proteins in *T. brucei* and their respective orthologues in trypanosomatids and *H. sapiens****.*

^1^ The Gene IDs for the members of the *H. sapiens* Hsp90/HSPC protein family were retrieved from NCBI (https://www.ncbi.nlm.nih.gov/).

^2^  The Gene IDs for the members of the *T. b. brucei*, *T. b. gambiense*, *T. cruzi*, *C. fasciculata*, *B. saltans* and *L. major* Hsp90/HSPC protein family were retrieved from the TriTrypDB database (http://tritrypdb.org/tritrypdb/; 78).

^3^The Gene IDs for the orthologues, identified by reciprocal BLASTP analysis, of three strains of *T. cruzi* are listed. *T. cruzi* CL Brener Esmeraldo-like (TcCLB), *T. cruzi* Dm28c 2018 (C4B63), and *T. cruzi* marinkelli strain B7 (Tc_MARK).
