# Supplementary material for: In silico analysis of the HSP90 chaperone system from the African trypanosome, Trypanosoma brucei
Source: Front Mol Biosci. 2022 Sep 23;9:947078. doi: 10.3389/fmolb.2022.947078 (PMC9538636; doi:10.3389/fmolb.2022.947078)
Supplement: Supplementary file 5 [file DataSheet3.docx]

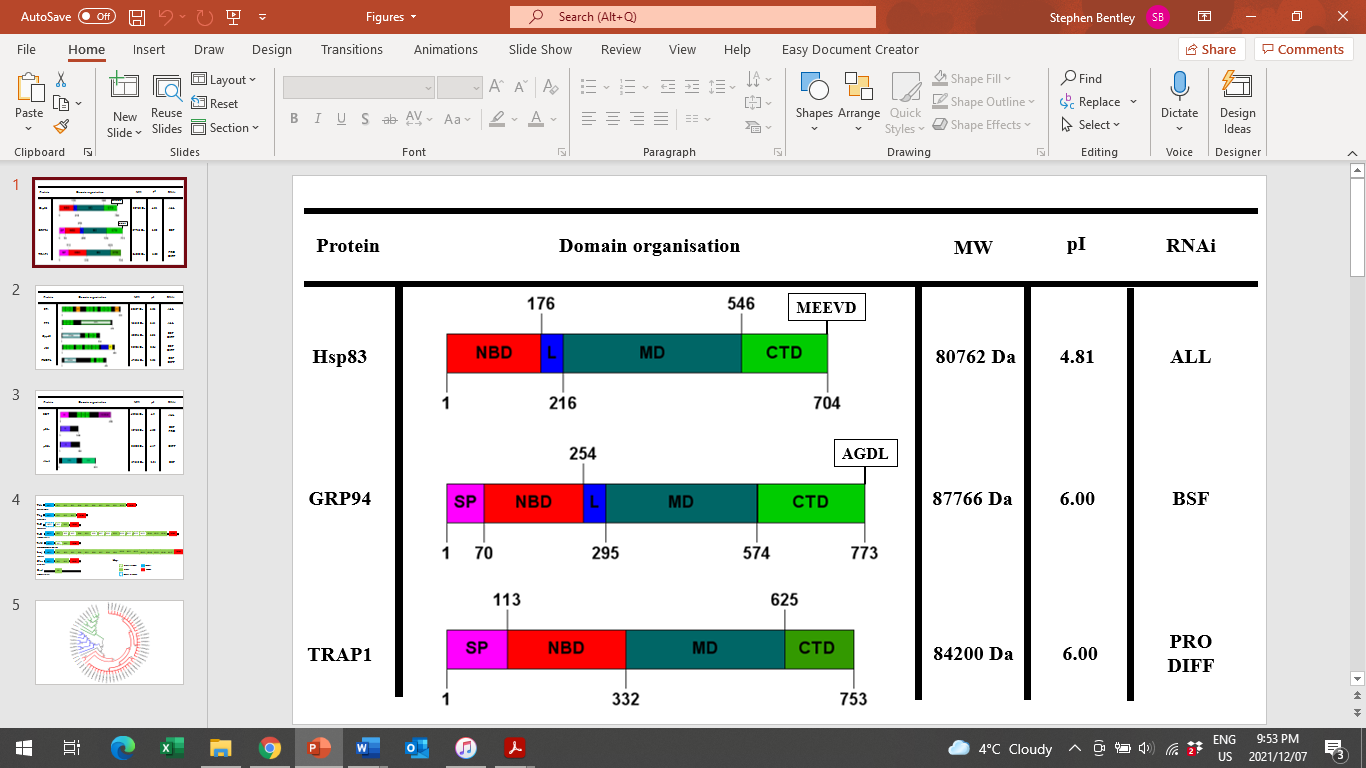


**Figure S3**: Schematic representation of the domain architecture of the Hsp90/HSPC proteins in *T. brucei*. Each protein sequence is represented by a coloured bar with the numbering on the bottom of the bar indicating the length of the protein in amino acid residues. Protein domains and other associated features that were identified using Pro-site (Sigrist et al., 2010) and SMART (Letunic et al., 2012) are also shown and include the N-terminal nucleotide binding domain (NBD; red), variable charger linker domain (L; dark blue), middle client protein-binding domain (MD; light blue), a C-terminal dimerization domain (CTD; green) and targeting signal peptides (SP; pink). The physiochemical properties, molecular weight (MW) and isoelectric point (pI), for each T. brucei Hsp90 protein was calculated using the compute pI/Mw tool from ExPASy (https://web.expasy.org/compute_pi/; (Gasteiger et al., 2005)). Data on the phenotypic knockdown screen on parasite viability, using RNAi conducted by Alsford et al. (Alsford et al., 2011), for each Hsp90/HSPC protein member is provided: ALL-Required for all lifecycle stages; BSF- Required for the bloodstream stage; PRO- Required for the procyclic stage; DIFF- Required for parasite differentiation.
