# Supplementary material for: In silico analysis of the HSP90 chaperone system from the African trypanosome, Trypanosoma brucei
Source: Front Mol Biosci. 2022 Sep 23;9:947078. doi: 10.3389/fmolb.2022.947078 (PMC9538636; doi:10.3389/fmolb.2022.947078)
Supplement: Supplementary file 6 [file DataSheet1.DOCX]

**Supplementary Figure S1**

SP

HsHSPC5 MARELRALLLWGRRLRPLLRAPALAAVPG---------------GKPILCPRRT-----T 40

LmjTRAP1 ----------MRRVVQRATVASAMAAASVSGVVLSKPS---SGVSPALSCGAG---GCTT 44

CfacTRAP1 ----------MRRAVQYAAVAPAMACVQMRQPAVAEGNVVSQIASQRAVCAAT--PTTTT 48

BsalTRAP1 ------------------------------------------------------------ 0

TbbTRAP1 ---------MMRRVCQRVNRQALTSTVV------ARCTATTASVSLRGICPPVSTDGNRN 45

TbgTRAP1 ---------MMRRVCQRVNRQALTSTVV------ARCTATTASVSLRGICPPVSTDGNRN 45

TcMTRAP1 ----------MRRVYQRICRDVLSHSTP------SGRAAFAAAIST--LSAASDSNRGDN 42

TcCLBTRAP1 ----------MRRVYQRICRDALSHSTP------SGRAAFAAAIST--LSAACDSSRGGN 42

TcDMTRAP1 ----------MRRVYQRICRDALIHSTP------SGRAAFAAAIST--LSAAYDSIRGGN 42

TbbGRP94 ------------------------------------------------------------ 0

TbgGRP94 ------------------------------------------------------------ 0

TcMGRP94 ------------------------------------------------------------ 0

TcCLBGRP94 ------------------------------------------------------------ 0

TcDMGRP94 ------------------------------------------------------------ 0

BsalGRP94 ------------------------------------------------------------ 0

LmjGRP94 ------------------------------------------------------------ 0

CfacGRP94 ------------------------------------------------------------ 0

HsHSPC4 ------------------------------------------------------------ 0

HsHSPC1 ---------------MP-------PCSGGDGSTPPGPS------LRDRDCPAQSAEYP-R 31

HsHSPC3 ------------------------------------------------------------ 0

BsalHsp83 ------------------------------------------------------------ 0

TbbHsp83-5 ------------------------------------------------------------ 0

TbbHsp83-9 ------------------------------------------------------------ 0

TbgHsp83-1 ------------------------------------------------------------ 0

TbgHsp83-2 ------------------------------------------------------------ 0

TbgHsp83-3 ------------------------------------------------------------ 0

TbbHsp83-10 ------------------------------------------------------------ 0

TbbHsp83-1 ------------------------------------------------------------ 0

TbbHsp83-2 ------------------------------------------------------------ 0

TbbHsp83-4 ------------------------------------------------------------ 0

TbbHsp83-6 ------------------------------------------------------------ 0

TbbHsp83-7 ------------------------------------------------------------ 0

TbbHsp83-8 ------------------------------------------------------------ 0

TbbHsp83-3 ------------------------------------------------------------ 0

TcMHsp83-2 ------------------------------------------------------------ 0

TcCLBHsp83-2 ------------------------------------------------------------ 0

TcDMHsp83-1 ------------------------------------------------------------ 0

TcDMHsp83-4 ------------------------------------------------------------ 0

TcDMHsp83-5 ------------------------------------------------------------ 0

TcDMHsp83-14 ------------------------------------------------------------ 0

TcDMHsp83-15 ------------------------------------------------------------ 0

TcDMHsp83-16 ------------------------------------------------------------ 0

TcDMHsp83-9 ------------------------------------------------------------ 0

CfacHsp83-1 ------------------------------------------------------------ 0

CfacHsp83-2 ------------------------------------------------------------ 0

LmjHsp83-1 ------------------------------------------------------------ 0

LmjHsp83-2 ------------------------------------------------------------ 0

LmjHsp83-3 ------------------------------------------------------------ 0

LmjHsp83-5 ------------------------------------------------------------ 0

LmjHsp83-6 ------------------------------------------------------------ 0

LmjHsp83-7 ------------------------------------------------------------ 0

LmjHsp83-9 ------------------------------------------------------------ 0

LmjHsp83-10 ------------------------------------------------------------ 0

LmjHsp83-11 ------------------------------------------------------------ 0

LmjHsp83-12 ------------------------------------------------------------ 0

LmjHsp83-13 ------------------------------------------------------------ 0

LmjHsp83-14 ------------------------------------------------------------ 0

LmjHsp83-15 ------------------------------------------------------------ 0

LmjHsp83-16 ------------------------------------------------------------ 0

LmjHsp83-17 ------------------------------------------------------------ 0

LmjHsp83-4 ------------------------------------------------------------ 0

LmjHsp83-8 ------------------------------------------------------------ 0

SP

HsHSPC5 AQLGPRRNP---------------------------AWSLQA----------------GR 57

LmjTRAP1 VTAATLTSAYRFCSTEKPATAAAT--EAEKKPKADASEELDE----------------DV 86

CfacTRAP1 ANLSAFCTARRFCSTEKSAAAAAAAAEADAKKPAATAEEMDE----------------DV 92

BsalTRAP1 ---------MSLQQRMSSSTT------------TPKDTTTDD----------------DV 23

TbbTRAP1 AGIGPMQGSVRFCSTQAGEKV------------PEAADNADE----------------DI 77

TbgTRAP1 VGIGPMQGSVRFCSTQAGEKV------------PEAADNADE----------------DI 77

TcMTRAP1 KMCAALATPMRFCSTSSDAAT------------RKPADITDE----------------DV 74

TcCLBTRAP1 KIRAALATPMRFCSTSSDAAT------------KKPADIIDE----------------DV 74

TcDMTRAP1 KIRAAMATPMRFCSTSSDSAT------------KKPADITDE----------------DV 74

TbbGRP94 ------------------------------------------MIQSGMFFALRVLFVVFV 18

TbgGRP94 ------------------------------------------MIQSGMFFALRVLFVVFV 18

TcMGRP94 ------------------------------------------MARHPI---LQTMLIALI 15

TcCLBGRP94 ------------------------------------------MARHPI---IQAILIALI 15

TcDMGRP94 ------------------------------------------MARHSI---IQAILIALI 15

BsalGRP94 ------------------------------------------MRLSLI---FRIACVALL 15

LmjGRP94 ------------------------------------------MANSSL---LRVVLVALL 15

CfacGRP94 ------------------------------------------MSSSPV---LRVVLVALL 15

HsHSPC4 -------------------------------------------MRALWVLGL---CCVLL 14

HsHSPC1 DRLDPRPGSPSEAS-----------SPPFLRSRAPVNWYQEKAQVFLWHLMVSGSTTLLC 80

HsHSPC3 ------------------------------------------------------------ 0

BsalHsp83 ------------------------------------------------------------ 0

TbbHsp83-5 ------------------------------------------------------------ 0

TbbHsp83-9 ------------------------------------------------------------ 0

TbgHsp83-1 ------------------------------------------------------------ 0

TbgHsp83-2 ------------------------------------------------------------ 0

TbgHsp83-3 ------------------------------------------------------------ 0

TbbHsp83-10 ------------------------------------------------------------ 0

TbbHsp83-1 ------------------------------------------------------------ 0

TbbHsp83-2 ------------------------------------------------------------ 0

TbbHsp83-4 ------------------------------------------------------------ 0

TbbHsp83-6 ------------------------------------------------------------ 0

TbbHsp83-7 ------------------------------------------------------------ 0

TbbHsp83-8 ------------------------------------------------------------ 0

TbbHsp83-3 ------------------------------------------------------------ 0

TcMHsp83-2 ------------------------------------------------------------ 0

TcCLBHsp83-2 ------------------------------------------------------------ 0

TcDMHsp83-1 ------------------------------------------------------------ 0

TcDMHsp83-4 ------------------------------------------------------------ 0

TcDMHsp83-5 ------------------------------------------------------------ 0

TcDMHsp83-14 ------------------------------------------------------------ 0

TcDMHsp83-15 ------------------------------------------------------------ 0

TcDMHsp83-16 ------------------------------------------------------------ 0

TcDMHsp83-9 ------------------------------------------------------------ 0

CfacHsp83-1 ------------------------------------------------------------ 0

CfacHsp83-2 ------------------------------------------------------------ 0

LmjHsp83-1 ------------------------------------------------------------ 0

LmjHsp83-2 ------------------------------------------------------------ 0

LmjHsp83-3 ------------------------------------------------------------ 0

LmjHsp83-5 ------------------------------------------------------------ 0

LmjHsp83-6 ------------------------------------------------------------ 0

LmjHsp83-7 ------------------------------------------------------------ 0

LmjHsp83-9 ------------------------------------------------------------ 0

LmjHsp83-10 ------------------------------------------------------------ 0

LmjHsp83-11 ------------------------------------------------------------ 0

LmjHsp83-12 ------------------------------------------------------------ 0

LmjHsp83-13 ------------------------------------------------------------ 0

LmjHsp83-14 ------------------------------------------------------------ 0

LmjHsp83-15 ------------------------------------------------------------ 0

LmjHsp83-16 ------------------------------------------------------------ 0

LmjHsp83-17 ------------------------------------------------------------ 0

LmjHsp83-4 ------------------------------------------------------------ 0

LmjHsp83-8 ------------------------------------------------------------ 0

SP

HsHSPC5 LFS----------T----QTAEDKE----------------------EPLHSIISSTESV 81

LmjTRAP1 IVEPAPENTS---AGANEV-DGSAT----------------------EATAGTSATVEKP 120

CfacTRAP1 IIEPVPENVT---TKGNEA----------------------------DAPKGAPVGAEKP 121

BsalTRAP1 VIEPVPEKAA---GS-----------------------------------TSASQDAENV 45

TbbTRAP1 VIDPVPDLKG---NAG--ESADEGS----------------------AGGVKANEDSEKV 110

TbgTRAP1 VIDPVPDLKG---NAG--ESVDEGS----------------------AGGVKANEDSEKV 110

TcMTRAP1 VIDPTPAAKD---GSTAADGASPS------------------------SSAKPNEDSERV 107

TcCLBTRAP1 VIDPTPAAKD---GSTGADGASPS-----------------------SSSAKPNEDSERV 108

TcDMTRAP1 VIDPTPAAKD---GSTGADGASTSS----------------------SSSAKPNEDSERV 109

TbbGRP94 MLTSAPVEIALGDDSEL--------------------------------------KSNAT 40

TbgGRP94 MLTSAPVEIALGDDSEL--------------------------------------KSNAT 40

TcMGRP94 VLGVAV------TGVTV--------------------------------------KDDGS 31

TcCLBGRP94 VLGVAV------TGVTV--------------------------------------KDDGS 31

TcDMGRP94 VLGVAV------TGVTV--------------------------------------KDDGS 31

BsalGRP94 AFAIV--------------------------------------------------GEESA 25

LmjGRP94 LLGSVT-------------------------------------------------V-SAG 25

CfacGRP94 LLSSIS-------------------------------------------------VTSAG 26

HsHSPC4 TFGSVRADDEVDVDGTVEEDLGKSREGSRTDDEVVQREEEAIQL-----DGLNASQIREL 69

HsHSPC1 LWKQPFHVSAFPVTASLA--FRQSQ-G--AGQHLYKDLQPFILLRLLMPEETQTQDQPME 135

HsHSPC3 -----------------------------------------------MPEE-----VHHG 8

BsalHsp83 ------------------------------------------------------------ 0

TbbHsp83-5 ------------------------------------------------------------ 0

TbbHsp83-9 ------------------------------------------------------------ 0

TbgHsp83-1 ------------------------------------------------------------ 0

TbgHsp83-2 ------------------------------------------------------------ 0

TbgHsp83-3 ------------------------------------------------------------ 0

TbbHsp83-10 ------------------------------------------------------------ 0

TbbHsp83-1 ------------------------------------------------------------ 0

TbbHsp83-2 ------------------------------------------------------------ 0

TbbHsp83-4 ------------------------------------------------------------ 0

TbbHsp83-6 ------------------------------------------------------------ 0

TbbHsp83-7 ------------------------------------------------------------ 0

TbbHsp83-8 ------------------------------------------------------------ 0

TbbHsp83-3 ------------------------------------------------------------ 0

TcMHsp83-2 ------------------------------------------------------------ 0

TcCLBHsp83-2 ------------------------------------------------------------ 0

TcDMHsp83-1 ------------------------------------------------------------ 0

TcDMHsp83-4 ------------------------------------------------------------ 0

TcDMHsp83-5 ------------------------------------------------------------ 0

TcDMHsp83-14 ------------------------------------------------------------ 0

TcDMHsp83-15 ------------------------------------------------------------ 0

TcDMHsp83-16 ------------------------------------------------------------ 0

TcDMHsp83-9 ------------------------------------------------------------ 0

CfacHsp83-1 ------------------------------------------------------------ 0

CfacHsp83-2 ------------------------------------------------------------ 0

LmjHsp83-1 ------------------------------------------------------------ 0

LmjHsp83-2 ------------------------------------------------------------ 0

LmjHsp83-3 ------------------------------------------------------------ 0

LmjHsp83-5 ------------------------------------------------------------ 0

LmjHsp83-6 ------------------------------------------------------------ 0

LmjHsp83-7 ------------------------------------------------------------ 0

LmjHsp83-9 ------------------------------------------------------------ 0

LmjHsp83-10 ------------------------------------------------------------ 0

LmjHsp83-11 ------------------------------------------------------------ 0

LmjHsp83-12 ------------------------------------------------------------ 0

LmjHsp83-13 ------------------------------------------------------------ 0

LmjHsp83-14 ------------------------------------------------------------ 0

LmjHsp83-15 ------------------------------------------------------------ 0

LmjHsp83-16 ------------------------------------------------------------ 0

LmjHsp83-17 ------------------------------------------------------------ 0

LmjHsp83-4 ------------------------------------------------------------ 0

LmjHsp83-8 ------------------------------------------------------------ 0

SP

NBD

HsHSPC5 QGSTSKHEFQAETKKLLDIVARSLYSEKEVFIRELISNASDALEKLRHKLVSDGQALPE- 140

LmjTRAP1 VGESEEMGFKTETRQLLDIVACSLYSDKEVFIRELVSNASDALEKRHLLELSNPE-YARE 179

CfacTRAP1 VGESEEMGFKTETRQLLDIVACSLYSDKEVFIRELVSNASDALEKRHLVELSNPD-FARA 180

BsalTRAP1 VGEAEEMGFKTETRQLLDIVACSLYSDKEVFVRELVSNASDALEKRRHLEVTNPEEYGRE 105

TbbTRAP1 VGSAEEMGFKTETRQLLDIVACSLYTEKEVFIRELVSNASDALEKRHLMELSKPEEYPRE 170

TbgTRAP1 VGSAEEMGFKTETRQLLDIVACSLYTEKEVFIRELVSNASDALEKRHLMELSKPEEYPRE 170

TcMTRAP1 VGEPEEMGFKTETRQLLDIVACSLYTEKEVFIRELVSNSSDALEKRHLLEISKPEEYPRE 167

TcCLBTRAP1 VGEPEEMGFKTETRQLLDIVACSLYTEKEVFIRELVSNSSDALEKRHLLEISKPEEYPRE 168

TcDMTRAP1 VGESEEMGFKTETRQLLDIVACSLYTEKEVFIRELVSNSSDALEKRHLLEISKPEEYPRE 169

TbbGRP94 FSKGKSIPFQAEVSKMLDILIHSLYTNRAVFLRELISNGSDALDKIRMLYLTTPKEPVNK 100

TbgGRP94 FSKGKSIPFQAEVSKMLDILIHSLYTNRAVFLRELISNGSDALDKIRMLYLTTPKEPVNK 100

TcMGRP94 VEKGKPISFQAEVSKMLDILINSLYTNRAVFLRELISNGSDALDKIRMLYLTAPKEPKNK 91

TcCLBGRP94 VEKGRPISFQAEVSKMLDILINSLYTNRAVFLRELISNGSDALDKIRMLYLTAPKEPKNK 91

TcDMGRP94 VEKGRPISFQAEVSKMLDILINSLYTNRAVFLRELISNGSDALDKIRMLYLTAPKEPKNK 91

BsalGRP94 SGKGSPITFQAEVSKMLDILINSLYTNRNIFLREIISNASDALDKIRFFYLTTPREPTNA 85

LmjGRP94 DGRGTPIAFQAEVSKMLDILVNSLYTNRAVFLRELISNGSDALDKIRVLYLTSPKEPLTK 85

CfacGRP94 DGRGAPITFQAEVSKMLDILVNSLYTNRAIFLRELISNGSDALDKIRVLYLTSPKEPLNA 86

HsHSPC4 REKSEKFAFQAEVNRMMKLIINSLYKNKEIFLRELISNASDALDKIRLISLTDEN----A 125

HsHSPC1 EEEVETFAFQAEIAQLMSLIINTFYSNKEIFLRELISNSSDALDKIRYESLTDPS----K 191

HsHSPC3 EEEVETFAFQAEIAQLMSLIINTFYSNKEIFLRELISNASDALDKIRYESLTDPS----K 64

BsalHsp83 -MASETFAFQAEINQLMSLIINTFYSNKEIFLRELISNASDACDKIRYQSLTNKD----V 55

TbbHsp83-5 --MTETFAFQAEINQLMSLIINTFYSNKEIFLRELISNSSDACDKIRYQSLTSQS----V 54

TbbHsp83-9 --MTETFAFQAEINQLMSLIINTFYSNKEIFLRELISNSSDACDKIRYQSLTNQS----V 54

TbgHsp83-1 --MTETFAFQAEINQLMSLIINTFYSNKEIFLRELISNSSDACDKIRYQSLTNQS----V 54

TbgHsp83-2 --MTETFAFQAEINQLMSLIINTFYSNKEIFLRELISNSSDACDKIRYQSLTNQS----V 54

TbgHsp83-3 --MTETFAFQAEINQLMSLIINTFYSNKEIFLRELISNSSDACDKIRYQSLTNQS----V 54

TbbHsp83-10 --MTETFAFQAEINQLMSLIINTFYSNKEIFLRELISNSSDACDKIRYQSLTNQS----V 54

TbbHsp83-1 --MTETFAFQAEINQLMSLIINTFYSNKEIFLRELISNSSDACDKIRYQSLTNQS----V 54

TbbHsp83-2 --MTETFAFQAEINQLMSLIINTFYSNKEIFLRELISNSSDACDKIRYQSLTNQS----V 54

TbbHsp83-4 --MTETFAFQAEINQLMSLIINTFYSNKEIFLRELISNSSDACDKIRYQSLTNQS----V 54

TbbHsp83-6 --MTETFAFQAEINQLMSLIINTFYSNKEIFLRELISNSSDACDKIRYQSLTNQS----V 54

TbbHsp83-7 --MTETFAFQAEINQLMSLIINTFYSNKEIFLRELISNSSDACDKIRYQSLTNQS----V 54

TbbHsp83-8 --MTETFAFQAEINQLMSLIINTFYSNKEIFLRELISNSSDACDKIRYQSLTNQS----V 54

TbbHsp83-3 --MTETFAFQAEINQLMSLIINTFYSNKEIFLRELISNSSDACDKIRYQSLTNQS----V 54

TcMHsp83-2 --MTETFAFQAEINQLMSLIINTFYSNKEIFLRELISNSSDACDKIRYQSLTNQA----V 54

TcCLBHsp83-2 --MTETFAFQAEINQLMSLIINTFYSNKEIFLRELISNSSDACDKIRYQSLTNQA----V 54

TcDMHsp83-1 --MTETFAFQAEINQLMSLIINTFYSNKEIFLRELISNSSDACDKIRYQSLTNQA----V 54

TcDMHsp83-4 --MTETFAFQAEINQLMSLIINTFYSNKEIFLRELISNSSDACDKIRYQSLTNQA----V 54

TcDMHsp83-5 --MTETFAFQAEINQLMSLIINTFYSNKEIFLRELISNSSDACDKIRYQSLTNQA----V 54

TcDMHsp83-14 --MTETFAFQAEINQLMSLIINTFYSNKEIFLRELISNSSDACDKIRYQSLTNQA----V 54

TcDMHsp83-15 --MTETFAFQAEINQLMSLIINTFYSNKEIFLRELISNSSDACDKIRYQSLTNQA----V 54

TcDMHsp83-16 --MTETFAFQAEINQLMSLIINTFYSNKEIFLRELISNSSDACDKIRYQSLTNQA----V 54

TcDMHsp83-9 --MTETFAFQAEINQLMSLIINTFYSNKEIFLRELISNSSDACDKIRYQSLTNQA----V 54

CfacHsp83-1 --MTETFAFQAEINQLMSLIINTFYSNKEIFLRELISNASDACDKIRYQSLTDPS----V 54

CfacHsp83-2 --MTETFAFQAEINQLMSLIINTFYSNKEIFLRELISNASDACDKIRYQSLTDPS----V 54

LmjHsp83-1 --MTETFAFQAEINQLMSLIINTFYSNKEIFLRELISNASDACDKIRYQSLTDPS----V 54

LmjHsp83-2 --MTETFAFQAEINQLMSLIINTFYSNKEIFLRELISNASDACDKIRYQSLTDPS----V 54

LmjHsp83-3 --MTETFAFQAEINQLMSLIINTFYSNKEIFLRELISNASDACDKIRYQSLTDPS----V 54

LmjHsp83-5 --MTETFAFQAEINQLMSLIINTFYSNKEIFLRELISNASDACDKIRYQSLTDPS----V 54

LmjHsp83-6 --MTETFAFQAEINQLMSLIINTFYSNKEIFLRELISNASDACDKIRYQSLTDPS----V 54

LmjHsp83-7 --MTETFAFQAEINQLMSLIINTFYSNKEIFLRELISNASDACDKIRYQSLTDPS----V 54

LmjHsp83-9 --MTETFAFQAEINQLMSLIINTFYSNKEIFLRELISNASDACDKIRYQSLTDPS----V 54

LmjHsp83-10 --MTETFAFQAEINQLMSLIINTFYSNKEIFLRELISNASDACDKIRYQSLTDPS----V 54

LmjHsp83-11 --MTETFAFQAEINQLMSLIINTFYSNKEIFLRELISNASDACDKIRYQSLTDPS----V 54

LmjHsp83-12 --MTETFAFQAEINQLMSLIINTFYSNKEIFLRELISNASDACDKIRYQSLTDPS----V 54

LmjHsp83-13 --MTETFAFQAEINQLMSLIINTFYSNKEIFLRELISNASDACDKIRYQSLTDPS----V 54

LmjHsp83-14 --MTETFAFQAEINQLMSLIINTFYSNKEIFLRELISNASDACDKIRYQSLTDPS----V 54

LmjHsp83-15 --MTETFAFQAEINQLMSLIINTFYSNKEIFLRELISNASDACDKIRYQSLTDPS----V 54

LmjHsp83-16 --MTETFAFQAEINQLMSLIINTFYSNKEIFLRELISNASDACDKIRYQSLTDPS----V 54

LmjHsp83-17 --MTETFAFQAEINQLMSLIINTFYSNKEIFLRELISNASDACDKIRYQSLTDPS----V 54

LmjHsp83-4 --MTETFAFQAEINQLMSLIINTFYSNKEIFLRELISNASDACDKIRYQSLTDPS----V 54

LmjHsp83-8 --MTETFAFQAEINQLMSLIINTFYSNKEIFLRELISNASDACDKIRYQSLTDPS----V 54

*::* :::.:: ::*.:: :*:**::**.*** :* : ::

NBD

HsHSPC5 ------MEIHLQTNAEKGTITIQDTGIGMTQEELVSNLGTIARSGSKAFLDALQNQAE-- 192

LmjTRAP1 -PADEAPLIALSCNQSKSRFIIRDTGIGMTREELTANLGTIAGSGSKAFVHELQSSGK-- 236

CfacTRAP1 -SDDEAPLIAISCNQSKSRFIIRDTGIGMTREELAANLGTIAGSGSKAFVRELQSSGQ-- 237

BsalTRAP1 -EGDEAMNISLTCNQSKSRFVVRDTGIGMTKEAFFVRDTGIG------------MTKEEL 152

TbbTRAP1 -EGDEAPIISITCNQSKSRFVIRDTGIGMTREELAENLGTIAGSGSKAFVRELQSQGESS 229

TbgTRAP1 -EGDEAPIISITCNQSKSRFVIRDTGIGMTREELAENLGTIAGSGSKAFVRELQSQGESS 229

TcMTRAP1 -EDDEAPLIAISCNQSKSRFVIRDTGVGMTREELAENLGTIAGSGSKEFVRELQNAAA-G 225

TcCLBTRAP1 -EDDEAPLIAISCNQSKSRFVIRDTGVGMTREELAENLGTIAGSGSKEFVRELQNAAS-G 226

TcDMTRAP1 -EDDEAPLIAISCNQSKSRFVIRDTGVGMTREELAENLGTIAGSGSKEFVRELQSAAS-G 227

TbbGRP94 DGEAPTMDIRLSVDPEQKTLTLRDGGVGMTRQELEANLGSLGSSGTKRFMEKLQETK--- 157

TbgGRP94 DGEAPTMDIRLSVDPEQKTLTLRDGGVGMTRQELEANLGSLGSSGTKRFMEKLQETK--- 157

TcMGRP94 DGEVPALEMRVIIDNERKTLTLRDGGIGMTKAELEEHLGSLGTSGTKRFMEKLKETK--- 148

TcCLBGRP94 DGEVPALEMRVIIDNERKTLTLRDGGIGMTKAELEEHLGSLGTSGTKRFMEKLKETK--- 148

TcDMGRP94 EGEVPALEMRVIIDNERKTLTLRDGGIGMTKAELEEHLGSLGASGTKRFMEKLKETK--- 148

BsalGRP94 NGEAPTMDIRIVVDREKRLFIMRDGGVGMTKEELAANLGSLGSSGTKRFLEKMKDSS--- 142

LmjGRP94 DGEAPTMDLRISFDKEKSELILRDGGVGMTKEELAKHLGSLGTSGTKHFLEKLQEGVGAG 145

CfacGRP94 DGAAPSMDIRISFNKDKSELVIRDGGVGMNKDELAGHLGSLGTSGTKRFLEKMQEGG--A 144

HsHSPC4 LSGNEELTVKIKCDKEKNLLHVTDTGVGMTREELVKNLGTIAKSGTSEFLNKMTEAQEDG 185

HsHSPC1 LDSGKELHINLIPNKQDRTLTIVDTGIGMTKADLINNLGTIAKSGTKAFMEALQAG---- 247

HsHSPC3 LDSGKELKIDIIPNPQERTLTLVDTGIGMTKADLINNLGTIAKSGTKAFMEALQAG---- 120

BsalHsp83 LGTETHLRIRVIPDKANKTITLWDSGIGMTKADMVNNLGTIARSGTKAFMEAIECG---- 111

TbbHsp83-5 LGDEPHLRIRVIPDRVNKTLTVEDSGIGMTKADLVNNLGTIARSGTKSFMEALEAG---- 110

TbbHsp83-9 LGDEPHLRIRVIPDRVNKTLTVEDSGIGMTKADLVNNLGTIARSGTKSFMEALEAG---- 110

TbgHsp83-1 LGDEPHLRIRVIPDRVNKTLTVEDSGIGMTKADLVNNLGTIARSGTKSFMEALEAG---- 110

TbgHsp83-2 LGDEPHLRIRVIPDRVNKTLTVEDSGIGMTKADLVNNLGTIARSGTKSFMEALEAG---- 110

TbgHsp83-3 LGDEPHLRIRVIPDRVNKTLTVEDSGIGMTKADLVNNLGTIARSGTKSFMEALEAG---- 110

TbbHsp83-10 LGDEPHLRIRVIPDRVNKTLTVEDSGIGMTKADLVNNLGTIARSGTKSFMEALEAG---- 110

TbbHsp83-1 LGDEPHLRIRVIPDRVNKTLTVEDSGIGMTKADLVNNLGTIARSGTKSFMEALEAG---- 110

TbbHsp83-2 LGDEPHLRIRVIPDRVNKTLTVEDSGIGMTKADLVNNLGTIARSGTKSFMEALEAG---- 110

TbbHsp83-4 LGDEPHLRIRVIPDRVNKTLTVEDSGIGMTKADLVNNLGTIARSGTKSFMEALEAG---- 110

TbbHsp83-6 LGDEPHLRIRVIPDRVNKTLTVEDSGIGMTKADLVNNLGTIARSGTKSFMEALEAG---- 110

TbbHsp83-7 LGDEPHLRIRVIPDRVNKTLTVEDSGIGMTKADLVNNLGTIARSGTKSFMEALEAG---- 110

TbbHsp83-8 LGDEPHLRIRVIPDRVNKTLTVEDSGIGMTKADLVNNLGTIARSGTKSFMEALEAG---- 110

TbbHsp83-3 LGDEPHLRIRVIPDRVNKTLTVEDSGIGMTKADLVNNLGTIARSGTKSFMEALEAG---- 110

TcMHsp83-2 LGDESHLRVRVIPDKANKTLTVEDTGIGMTKAELVNNLGTIARSGTKAFMEALEAG---- 110

TcCLBHsp83-2 LGDESHLRIRVIPDKANKTLTVEDTGIGMTKAELVNNLGTIARSGTKAFMEALEAG---- 110

TcDMHsp83-1 LGDESHLRIRVVPDKANKTLTVEDTGIGMTKAELVNNLGTIARSGTKAFMEALEAG---- 110

TcDMHsp83-4 LGDESHLRIRVVPDKANKTLTVEDTGIGMTKAELVNNLGTIARSGTKAFMEALEAG---- 110

TcDMHsp83-5 LGDESHLRIRVVPDKANKTLTVEDTGIGMTKAELVNNLGTIARSGTKAFMEALEAG---- 110

TcDMHsp83-14 LGDESHLRIRVVPDKANKTLTVEDTGIGMTKAELVNNLGTIARSGTKAFMEALEAG---- 110

TcDMHsp83-15 LGDESHLRIRVVPDKANKTLTVEDTGIGMTKAELVNNLGTIARSGTKAFMEALEAG---- 110

TcDMHsp83-16 LGDESHLRIRVVPDKANKTLTVEDTGIGMTKAELVNNLGTIARSGTKAFMEALEAG---- 110

TcDMHsp83-9 LGDESHLRIRVVPDKANKTLTVEDTGIGMTKAELVNNLGTIARSGTKAFMEALEAG---- 110

CfacHsp83-1 LGDETRLRIRVIPDKANKTLTVEDNGIGMTKADLVNNLGTIARSGTKAFMEALEAG---- 110

CfacHsp83-2 LGDETRLRIRVIPDKANKTLTVEDNGIGMTKADLVNNLGTIARSGTKAFMEALEAG---- 110

LmjHsp83-1 LGESPRLCIRVVPDKENKTLTVEDNGIGMTKADLVNNLGTIARSGTKAFMEALEAG---- 110

LmjHsp83-2 LGESPRLCIRVVPDKENKTLTVEDNGIGMTKADLVNNLGTIARSGTKAFMEALEAG---- 110

LmjHsp83-3 LGESPRLCIRVVPDKENKTLTVEDNGIGMTKADLVNNLGTIARSGTKAFMEALEAG---- 110

LmjHsp83-5 LGESPRLCIRVVPDKENKTLTVEDNGIGMTKADLVNNLGTIARSGTKAFMEALEAG---- 110

LmjHsp83-6 LGESPRLCIRVVPDKENKTLTVEDNGIGMTKADLVNNLGTIARSGTKAFMEALEAG---- 110

LmjHsp83-7 LGESPRLCIRVVPDKENKTLTVEDNGIGMTKADLVNNLGTIARSGTKAFMEALEAG---- 110

LmjHsp83-9 LGESPRLCIRVVPDKENKTLTVEDNGIGMTKADLVNNLGTIARSGTKAFMEALEAG---- 110

LmjHsp83-10 LGESPRLCIRVVPDKENKTLTVEDNGIGMTKADLVNNLGTIARSGTKAFMEALEAG---- 110

LmjHsp83-11 LGESPRLCIRVVPDKENKTLTVEDNGIGMTKADLVNNLGTIARSGTKAFMEALEAG---- 110

LmjHsp83-12 LGESPRLCIRVVPDKENKTLTVEDNGIGMTKADLVNNLGTIARSGTKAFMEALEAG---- 110

LmjHsp83-13 LGESPRLCIRVVPDKENKTLTVEDNGIGMTKADLVNNLGTIARSGTKAFMEALEAG---- 110

LmjHsp83-14 LGESPRLCIRVVPDKENKTLTVEDNGIGMTKADLVNNLGTIARSGTKAFMEALEAG---- 110

LmjHsp83-15 LGESPRLCIRVVPDKENKTLTVEDNGIGMTKADLVNNLGTIARSGTKAFMEALEAG---- 110

LmjHsp83-16 LGESPRLCIRVVPDKENKTLTVEDNGIGMTKADLVNNLGTIARSGTKAFMEALEAG---- 110

LmjHsp83-17 LGESPRLCIRVVPDKENKTLTVEDNGIGMTKADLVNNLGTIARSGTKAFMEALEAG---- 110

LmjHsp83-4 LGESPRLCIRVVPDKENKTLTVEDNGIGMTKADLVNNLGTIARSGTKAFMEALEAG---- 110

LmjHsp83-8 LGESPRLCIRVVPDKENKTLTVEDNGIGMTKADLVNNLGTIARSGTKAFMEALEAG---- 110

: : : : : * *:**.: : . :.

NBD

HsHSPC5 --ASSKIIGQFGVGFYSAFMVADRVEVYSRSAAPGSLGYQWLSDGSGVFEIA-E--ASGV 247

LmjTRAP1 -SAAEKIIGQFGVGFYACFMVAKNVKVYSRSAKKGSKGYLWESEGTGTFKVT-E--CEGV 292

CfacTRAP1 -SAAEKIIGQFGVGFYACFMVAKSVKVYSRSAKKDSKGYLWESDGTGTFKIT-E--CEGV 293

BsalTRAP1 GAAADKIIGQFGVGFYSAFMVSKYVKVYSRSAKKGSKGYLWESDGTGTFKIS-E--CEGV 209

TbbTRAP1 SGAAEKIIGQFGVGFYAAFMVARNVKVYSRSVKKGSKGYVWESDGTGTFKIA-E--CEGV 286

TbgTRAP1 SGAAEKIIGQFGVGFYAAFMVARNVKVYSRSVKKGSKGYVWESDGTGTFKIA-E--CEGV 286

TcMTRAP1 AQAAEKIIGQFGVGFYASFMVAKHVKVYSRSAKKGSKGYLWESDGTGTFKIT-E--CEGV 282

TcCLBTRAP1 AQAAEKIIGQFGVGFYASFMVAKHVKVYSRSAKKGSKGFLWESDGTGTFKIT-E--CEGV 283

TcDMTRAP1 AQAAEKIIGQFGVGFYASFMVAKHVKVFSRSAKKGSKGYLWESDGTGTFKIT-E--CEGV 284

TbbGRP94 ---DSNLIGQFGVGFYSAFLVAERVRVASKSDD-DEKQWVWESAADGQYYVYEDERGNTL 213

TbgGRP94 ---DSNLIGQFGVGFYSAFLVAERVRVASKSDD-DEKQWVWESAADGQYYVYEDERGNTL 213

TcMGRP94 ---DDSLIGQFGVGFYSAFLVADRVRVASKSDD-SDVQWVWESAGDGQYYIYEDERGNTL 204

TcCLBGRP94 ---DDSLIGQFGVGFYSAFLVADRVRVASKSDD-SDVQWVWESAGDGQYYIYEDERGNTL 204

TcDMGRP94 ---DDSLIGQFGVGFYSAFLVADRVRVASKSDD-SDVQWVWESAGDGQYYIYEDERGNTL 204

BsalGRP94 ---DANFIGQFGVGFYSVFLVADKVRVASKHDD-SEKQWVWESTGDGTFFLYEDERGNTL 198

LmjGRP94 GGDQNNLIGQFGVGFYSVFLVGDRVRVASKSDD-SDEQYVWESKGDGQYFLYPDPRGNTL 204

CfacGRP94 AGDQNNLIGQFGVGFYSVFLVGDRVRVASKSDD-SDEQYVWESTGNGQYFLYPDPRGNTL 203

HsHSPC4 Q-STSELIGQFGVGFYSAFLVADKVIVTSKHNN--DTQHIWESDSNEFS-VIADPRGNTL 241

HsHSPC1 --ADISMIGQFGVGFYSAYLVAEKVTVITKHND--DEQYAWESSAGGSF-TVRTDTGEPM 302

HsHSPC3 --ADISMIGQFGVGFYSAYLVAEKVVVITKHND--DEQYAWESSAGGSF-TVRADHGEPI 175

BsalHsp83 --GDISMIGQFGVGFYSAYLIADRVTVVSKHND--DDAYVWESSAGGTF-TVTPYSGTDM 166

TbbHsp83-5 --GDMSMIGQFGVGFYSAYLVADRVTVVSKNNE--DDAYTWESSAGGTF-TVTSTPDCDL 165

TbbHsp83-9 --GDMSMIGQFGVGFYSAYLVADRVTVVSKNNE--DDAYTWESSAGGTF-TVTSTPDCDL 165

TbgHsp83-1 --GDMSMIGQFGVGFYSAYLVADRVTVVSKNNE--DDAYTWESSAGGTF-TVTSTPDCDL 165

TbgHsp83-2 --GDMSMIGQFGVGFYSAYLVADRVTVVSKNNE--DDAYTWESSAGGTF-TVTSTPDCDL 165

TbgHsp83-3 --GDMSMIGQFGVGFYSAYLVADRVTVVSKNNE--DDAYTWESSAGGTF-TVTSTPDCDL 165

TbbHsp83-10 --GDMSMIGQFGVGFYSAYLVADRVTVVSKNNE--DDAYTWESSAGGTF-TVTSTPDCDL 165

TbbHsp83-1 --GDMSMIGQFGVGFYSAYLVADRVTVVSKNNE--DDAYTWESSAGGTF-TVTSTPDCDL 165

TbbHsp83-2 --GDMSMIGQFGVGFYSAYLVADRVTVVSKNNE--DDAYTWESSAGGTF-TVTSTPDCDL 165

TbbHsp83-4 --GDMSMIGQFGVGFYSAYLVADRVTVVSKNNE--DDAYTWESSAGGTF-TVTSTPDCDL 165

TbbHsp83-6 --GDMSMIGQFGVGFYSAYLVADRVTVVSKNNE--DDAYTWESSAGGTF-TVTSTPDCDL 165

TbbHsp83-7 --GDMSMIGQFGVGFYSAYLVADRVTVVSKNNE--DDAYTWESSAGGTF-TVTSTPDCDL 165

TbbHsp83-8 --GDMSMIGQFGVGFYSAYLVADRVTVVSKNNE--DDAYTWESSAGGTF-TVTSTPDCDL 165

TbbHsp83-3 --GDMSMIGQFGVGFYSAYLVADRVTVVSKNNE--DDAYTWESSAGGTF-TVTSTPDCDL 165

TcMHsp83-2 --GDMSMIGQFGVGFYSAYLVADRVTVVSKNND--DEAYTWESSAGGTF-TVTPTPDCDL 165

TcCLBHsp83-2 --GDMSMIGQFGVGFYSAYLVADRVTVVSKNND--DEAYTWESSAGGTF-TVTPTPDCDL 165

TcDMHsp83-1 --GDMSMIGQFGVGFYSAYLVADRVTVVSKNND--DEAYTWESSAGGTF-TVTPTPDCDL 165

TcDMHsp83-4 --GDMSMIGQFGVGFYSAYLVADRVTVVSKNND--DEAYTWESSAGGTF-TVTPTPDCDL 165

TcDMHsp83-5 --GDMSMIGQFGVGFYSAYLVADRVTVVSKNND--DEAYTWESSAGGTF-TVTPTPDCDL 165

TcDMHsp83-14 --GDMSMIGQFGVGFYSAYLVADRVTVVSKNND--DEAYTWESSAGGTF-TVTPTPDCDL 165

TcDMHsp83-15 --GDMSMIGQFGVGFYSAYLVADRVTVVSKNND--DEAYTWESSAGGTF-TVTPTPDCDL 165

TcDMHsp83-16 --GDMSMIGQFGVGFYSAYLVADRVTVVSKNND--DEAYTWESSAGGTF-TVTPTPDCDL 165

TcDMHsp83-9 --GDMSMIGQFGVGFYSAYLVADRVTVVSKNND--DEAYTWESSAGGTF-TVTPTPDCDL 165

CfacHsp83-1 --GDMSMIGQFGVGFYSAYLVADRVTVVSKNNA--DEAYVWESSAGGTF-TIASVADSDL 165

CfacHsp83-2 --GDMSMIGQFGVGFYSAYLVADRVTVVSKNNA--DEAYVWESSAGGTF-TIASVADSDL 165

LmjHsp83-1 --GDMSMIGQFGVGFYSAYLVADRVTVTSKNNS--DESYVWESSAGGTF-TITSTPESDM 165

LmjHsp83-2 --GDMSMIGQFGVGFYSAYLVADRVTVTSKNNS--DESYVWESSAGGTF-TITSTPESDM 165

LmjHsp83-3 --GDMSMIGQFGVGFYSAYLVADRVTVTSKNNS--DESYVWESSAGGTF-TITSTPESDM 165

LmjHsp83-5 --GDMSMIGQFGVGFYSAYLVADRVTVTSKNNS--DESYVWESSAGGTF-TITSTPESDM 165

LmjHsp83-6 --GDMSMIGQFGVGFYSAYLVADRVTVTSKNNS--DESYVWESSAGGTF-TITSTPESDM 165

LmjHsp83-7 --GDMSMIGQFGVGFYSAYLVADRVTVTSKNNS--DESYVWESSAGGTF-TITSTPESDM 165

LmjHsp83-9 --GDMSMIGQFGVGFYSAYLVADRVTVTSKNNS--DESYVWESSAGGTF-TITSTPESDM 165

LmjHsp83-10 --GDMSMIGQFGVGFYSAYLVADRVTVTSKNNS--DESYVWESSAGGTF-TITSTPESDM 165

LmjHsp83-11 --GDMSMIGQFGVGFYSAYLVADRVTVTSKNNS--DESYVWESSAGGTF-TITSTPESDM 165

LmjHsp83-12 --GDMSMIGQFGVGFYSAYLVADRVTVTSKNNS--DESYVWESSAGGTF-TITSTPESDM 165

LmjHsp83-13 --GDMSMIGQFGVGFYSAYLVADRVTVTSKNNS--DESYVWESSAGGTF-TITSTPESDM 165

LmjHsp83-14 --GDMSMIGQFGVGFYSAYLVADRVTVTSKNNS--DESYVWESSAGGTF-TITSTPESDM 165

LmjHsp83-15 --GDMSMIGQFGVGFYSAYLVADRVTVTSKNNS--DESYVWESSAGGTF-TITSTPESDM 165

LmjHsp83-16 --GDMSMIGQFGVGFYSAYLVADRVTVTSKNNS--DESYVWESSAGGTF-TITSTPESDM 165

LmjHsp83-17 --GDMSMIGQFGVGFYSAYLVADRVTVTSKNNS--DESYVWESSAGGTF-TITSTPESDM 165

LmjHsp83-4 --GDMSMIGQFGVGFYSAYLVADRVTVTSKNNS--DESYVWESSAGGTF-TITSTPESDM 165

LmjHsp83-8 --GDMSMIGQFGVGFYSAYLVADRVTVTSKNNS--DESYVWESSAGGTF-TITSTPESDM 165

.:*********: :::. * * :: . * * . :

CLD

NBD

HsHSPC5 RTGTKIIIHLKSDCKEFSSEARVRDVVTKYSNFVSFPLYLNGR----------------- 290

LmjTRAP1 EKGTKIVLDVKDTELSFCTPQVVERVLKKYSNFVSYEITLNGG----------------- 335

CfacTRAP1 EKGTKIVLDVKDTELSFCTPQVVERVLKKYSNFVSFEITLNGG----------------- 336

BsalTRAP1 EKGTKIVLDVKDTELSFCTPQVCERVLKKYSNFVSFDITLNGG----------------- 252

TbbTRAP1 DKGTKIVLDVKDTELSFCTPQVCERVLKRYSNFVSYEITLNGG----------------- 329

TbgTRAP1 DKGTKIVLDVKDTELSFCTPQVCERVLKRYSNFVSYEITLNGG----------------- 329

TcMTRAP1 DKGTKIVLDVKDTELSFCTPQVCERVLKKYSNFVSYEITLNGG----------------- 325

TcCLBTRAP1 DKGTKIVLDVKDTELSFCTPQVCERVLKKYSNFVSYEITLNGG----------------- 326

TcDMTRAP1 DKGTKIVLDVKDTELSFCTPQVCERVLKKYSNFVSYEITLNGG----------------- 327

TbbGRP94 GRGTEITLELKPDALDFLSPETVRNTVRQYSEFVHFPIRMKR------------------ 255

TbgGRP94 GRGTEITLELKPDALDFLSPETVRNTVRQYSEFVHFPIRMKR------------------ 255

TcMGRP94 GRGTEITLEMKPDALEFLSTDNVRNIVHQYSEFVHFPIYMQK------------------ 246

TcCLBGRP94 GRGTEITLEMKPDALEFLSTDNVRDIVHQYSEFVHFPIYMQK------------------ 246

TcDMGRP94 GRGTEITLEMKPDALEFLSTDNVRDIVHQYSEFVHFPIYMQK------------------ 246

BsalGRP94 GRGSELTLELKKDADEYLDIDKVKEAIHKYSEFIHFPIYIQTTKTEKVKKAAE-AESTE- 256

LmjGRP94 GRGTEITIELKPDAEQFLSAETIKKTIHQYSEFINFPIYVQEEVEVASTAATPEPAAEEG 264

CfacGRP94 GRGTEITIEVKPDAEQFLSAETIKKTIHQYSEFINFPIYVEEEVAVEAAKKGG-AKEEEE 262

HsHSPC4 GRGTTITLVLKEEASDYLELDTIKNLVKKYSQFINFPIYVWSSKTETVEEPMEEEEAAKE 301

HsHSPC1 GRGTKVILHLKEDQTEYLEERRIKEIVKKHSQFIGYPITLFVEKERDKEVSDDEAEEKED 362

HsHSPC3 GRGTKVILHLKEDQTEYLEERRVKEVVKKHSQFIGYPITLYLEKEREKEISDDEAEEEKG 235

BsalHsp83 TRGTRIILHLKEDQQEYLEERRIKDLIKKHSEFIGYDIELQVEKTSEKEVTDEDEEEK-- 224

TbbHsp83-5 KRGTRIVLHLKEDQQEYLEERRLKDLIKKHSEFIGYDIELMVENTTEKEVTDEDEDEEAA 225

TbbHsp83-9 KRGTRIVLHLKEDQQEYLEERRLKDLIKKHSEFIGYDIELMVENTTEKEVTDEDEDEEAA 225

TbgHsp83-1 KRGTRIVLHLKEDQQEYLEERRLKDLIKKHSEFIGYDIELMVENTTEKEVTDEDEDEEAA 225

TbgHsp83-2 KRGTRIVLHLKEDQQEYLEERRLKDLIKKHSEFIGYDIELMVENTTEKEVTDEDEDEEAA 225

TbgHsp83-3 KRGTRIVLHLKEDQQEYLEERRLKDLIKKHSEFIGYDIELMVENTTEKEVTDEDEDEEAA 225

TbbHsp83-10 KRGTRIVLHLKEDQQEYLEERRLKDLIKKHSEFIGYDIELMVENTTEKEVTDEDEDEEAA 225

TbbHsp83-1 KRGTRIVLHLKEDQQEYLEERRLKDLIKKHSEFIGYDIELMVENTTEKEVTDEDEDEEAA 225

TbbHsp83-2 KRGTRIVLHLKEDQQEYLEERRLKDLIKKHSEFIGYDIELMVENTTEKEVTDEDEDEEAA 225

TbbHsp83-4 KRGTRIVLHLKEDQQEYLEERRLKDLIKKHSEFIGYDIELMVENTTEKEVTDEDEDEEAA 225

TbbHsp83-6 KRGTRIVLHLKEDQQEYLEERRLKDLIKKHSEFIGYDIELMVENTTEKEVTDEDEDEEAA 225

TbbHsp83-7 KRGTRIVLHLKEDQQEYLEERRLKDLIKKHSEFIGYDIELMVENTTEKEVTDEDEDEEAA 225

TbbHsp83-8 KRGTRIVLHLKEDQQEYLEERRLKDLIKKHSEFIGYDIELMVENTTEKEVTDEDEDEEAA 225

TbbHsp83-3 KRGTRIVLHLKEDQQEYLEERRLKDLIKKHSEFIGYDIELMVENTTEKEVTDEDEDEEAA 225

TcMHsp83-2 KRGTRIVLHLKEDQQEYLEERRLKDLIKKHSEFIGYDIELMVEKATEKEVTDEDEDEAAA 225

TcCLBHsp83-2 KRGTRIVLHLKEDQQEYLEERRLKDLIKKHSEFIGYDIELMVEKATEKEVTDEDEDEAAA 225

TcDMHsp83-1 KRGTRIVLHLKEDQQEYLEERRLKDLIKKHSEFIGYDIELMVEKATEKEVTDEDEDEAAA 225

TcDMHsp83-4 KRGTRIVLHLKEDQQEYLEERRLKDLIKKHSEFIGYDIELMVEKATEKEVTDEDEDEAAA 225

TcDMHsp83-5 KRGTRIVLHLKEDQQEYLEERRLKDLIKKHSEFIGYDIELMVEKATEKEVTDEDEDEAAA 225

TcDMHsp83-14 KRGTRIVLHLKEDQQEYLEERRLKDLIKKHSEFIGYDIELMVEKATEKEVTDEDEDEAAA 225

TcDMHsp83-15 KRGTRIVLHLKEDQQEYLEERRLKDLIKKHSEFIGYDIELMVEKATEKEVTDEDEDEAAA 225

TcDMHsp83-16 KRGTRIVLHLKEDQQEYLEERRLKDLIKKHSEFIGYDIELMVEKATEKEVTDEDEDEAAA 225

TcDMHsp83-9 KRGTRIVLHLKEDQQEYLEERRLKDLIKKHSEFIGYDIELMVEKATEKEVTDEDEDEAAA 225

CfacHsp83-1 KRGTRITLHLKEDQQEYLEERRVKELIKKHSEFIGYDIELLVEKTTEKEVTDEDEEEK-K 224

CfacHsp83-2 KRGTRITLHLKEDQQEYLEERRVKELIKKHSEFIGYDIELLVEKTTEKEVTDEDEEEK-K 224

LmjHsp83-1 KRGTRITLHLKEDQMEYLEPRRLKELIKKHSEFIGYDIELMVEKTTEKEVTDEDEEDT-K 224

LmjHsp83-2 KRGTRITLHLKEDQMEYLEPRRLKELIKKHSEFIGYDIELMVEKTTEKEVTDEDEEDT-K 224

LmjHsp83-3 KRGTRITLHLKEDQMEYLEPRRLKELIKKHSEFIGYDIELMVEKTTEKEVTDEDEEDT-K 224

LmjHsp83-5 KRGTRITLHLKEDQMEYLEPRRLKELIKKHSEFIGYDIELMVEKTTEKEVTDEDEEDT-K 224

LmjHsp83-6 KRGTRITLHLKEDQMEYLEPRRLKELIKKHSEFIGYDIELMVEKTTEKEVTDEDEEDT-K 224

LmjHsp83-7 KRGTRITLHLKEDQMEYLEPRRLKELIKKHSEFIGYDIELMVEKTTEKEVTDEDEEDT-K 224

LmjHsp83-9 KRGTRITLHLKEDQMEYLEPRRLKELIKKHSEFIGYDIELMVEKTTEKEVTDEDEEDT-K 224

LmjHsp83-10 KRGTRITLHLKEDQMEYLEPRRLKELIKKHSEFIGYDIELMVEKTTEKEVTDEDEEDT-K 224

LmjHsp83-11 KRGTRITLHLKEDQMEYLEPRRLKELIKKHSEFIGYDIELMVEKTTEKEVTDEDEEDT-K 224

LmjHsp83-12 KRGTRITLHLKEDQMEYLEPRRLKELIKKHSEFIGYDIELMVEKTTEKEVTDEDEEDT-K 224

LmjHsp83-13 KRGTRITLHLKEDQMEYLEPRRLKELIKKHSEFIGYDIELMVEKTTEKEVTDEDEEDT-K 224

LmjHsp83-14 KRGTRITLHLKEDQMEYLEPRRLKELIKKHSEFIGYDIELMVEKTTEKEVTDEDEEDT-K 224

LmjHsp83-15 KRGTRITLHLKEDQMEYLEPRRLKELIKKHSEFIGYDIELMVEKTTEKEVTDEDEEDT-K 224

LmjHsp83-16 KRGTRITLHLKEDQMEYLEPRRLKELIKKHSEFIGYDIELMVEKTTEKEVTDEDEEDT-K 224

LmjHsp83-17 KRGTRITLHLKEDQMEYLEPRRLKELIKKHSEFIGYDIELMVEKTTEKEVTDEDEEDT-K 224

LmjHsp83-4 KRGTRITLHLKEDQMEYLEPRRLKELIKKHSEFIGYDIELMVEKTTEKEVTDEDEEDT-K 224

LmjHsp83-8 KRGTRITLHLKEDQMEYLEPRRLKELIKKHSEFIGYDIELMVEKTTEKEVTDEDEEDT-K 224

*: : : :* .: . : ::*:*: : : :

CLD

HsHSPC5 ------------------------------------------------RMNTLQAIWMMD 302

LmjTRAP1 ------------------------------------------------KVNTVEALWMKD 347

CfacTRAP1 ------------------------------------------------KVNTVEALWMKD 348

BsalTRAP1 ------------------------------------------------KVNTVEALWMKE 264

TbbTRAP1 ------------------------------------------------KVNTVEALWMKD 341

TbgTRAP1 ------------------------------------------------KVNTVEALWMKD 341

TcMTRAP1 ------------------------------------------------KVNTVEALWMKD 337

TcCLBTRAP1 ------------------------------------------------KVNTVEALWMKD 338

TcDMTRAP1 ------------------------------------------------KVNTVEALWMKD 339

TbbGRP94 -------------------------------------------GEEWDVLNENQPIWTRK 272

TbgGRP94 -------------------------------------------GEEWDVLNENQPIWTRK 272

TcMGRP94 -------------------------------------------GEKWEVVNENKPIWTRK 263

TcCLBGRP94 -------------------------------------------GEKWEVVNENKPIWTRK 263

TcDMGRP94 -------------------------------------------GEKWEVVNENKPIWTRK 263

BsalGRP94 -----------AKEDGDD----------EAPAEEKKVEEEEVVTHDWELINENKPIWTRK 295

LmjGRP94 -----------SLDEGAV----------EEDSDKEGGTQGVAKERRWVLVNENRPIWTRP 303

CfacGRP94 -----------VLDEDAI----------ED-----DEETAPATERKWTLVNENRPIWTRP 296

HsHSPC4 ---E----KEESDDEAAV--------EEEEEEKKPKTKKVEKTVWDWELMNDIKPIWQRP 346

HsHSPC1 KEEEKEKEEKESEDKPEIEDVGSDEEEEKKDGDKKKKKKIKEKYIDQEELNKTKPIWTRN 422

HsHSPC3 ---EKEEEDKDDEEKPKIEDVGSDEEDDSGKDKKKKTKKIKEKYIDQEELNKTKPIWTRN 292

BsalHsp83 --------KADDDEEPKVEEVKD---------EKKKTKKVKEVTKEFEIQNKNKPIWTRD 267

TbbHsp83-5 -------KKAEEGEEPKVEEVKDGDDADA---KKKKTKKVKEVKQEFVVQNKHKPLWTRD 275

TbbHsp83-9 -------KKAEEGEEPKVEEVKDGDDADA---KKKKTKKVKEVKQEFVVQNKHKPLWTRD 275

TbgHsp83-1 -------KKAEEGEEPKVEEVKDGDDADA---KKKKTKKVKEVKQEFVVQNKHKPLWTRD 275

TbgHsp83-2 -------KKAEEGEEPKVEEVKDGDDADA---KKKKTKKVKEVKQEFVVQNKHKPLWTRD 275

TbgHsp83-3 -------KKAEEGEEPKVEEVKDGDDADA---KKKKTKKVKEVKQEFVVQNKHKPLWTRD 275

TbbHsp83-10 -------KKAEEGEEPKVEEVKDGDDADA---KKKKTKKVKEVKQEFVVQNKHKPLWTRD 275

TbbHsp83-1 -------KKAEEGEEPKVEEVKDGDDADA---KKKKTKKVKEVKQEFVVQNKHKPLWTRD 275

TbbHsp83-2 -------KKAEEGEEPKVEEVKDGDDADA---KKKKTKKVKEVKQEFVVQNKHKPLWTRD 275

TbbHsp83-4 -------KKAEEGEEPKVEEVKDGDDADA---KKKKTKKVKEVKQEFVVQNKHKPLWTRD 275

TbbHsp83-6 -------KKAEEGEEPKVEEVKDGDDADA---KKKKTKKVKEVKQEFVVQNKHKPLWTRD 275

TbbHsp83-7 -------KKAEEGEEPKVEEVKDGDDADA---KKKKTKKVKEVKQEFVVQNKHKPLWTRD 275

TbbHsp83-8 -------KKAEEGEEPKVEEVKDGDDADA---KKKKTKKVKEVKQEFVVQNKHKPLWTRD 275

TbbHsp83-3 -------KKAEEGEEPKVEEVKDGDDADA---KKKKTKKVKEVKQEFVVQNKHKPLWTRD 275

TcMHsp83-2 -------AKNEEGEEPKVEEVKDDAEEGE---KKKKTKKVKEVTQEFVVQNKHKPLWTRD 275

TcCLBHsp83-2 -------AKNEEGEEPKVEEVKDDAEEGE---KKKKTKKVKEVTQEFVVQNKHKPLWTRD 275

TcDMHsp83-1 -------TKNEEGEEPKVEEVKDDAEEGE---KKKKTKKVKEVTQEFVVQNKHKPLWTRD 275

TcDMHsp83-4 -------TKNEEGEEPKVEEVKDDAEEGE---KKKKTKKVKEVTQEFVVQNKHKPLWTRD 275

TcDMHsp83-5 -------TKNEEGEEPKVEEVKDDAEEGE---KKKKTKKVKEVTQEFVVQNKHKPLWTRD 275

TcDMHsp83-14 -------TKNEEGEEPKVEEVKDDAEEGE---KKKKTKKVKEVTQEFVVQNKHKPLWTRD 275

TcDMHsp83-15 -------TKNEEGEEPKVEEVKDDAEEGE---KKKKTKKVKEVTQEFVVQNKHKPLWTRD 275

TcDMHsp83-16 -------TKNEEGEEPKVEEVKDDAEEGE---KKKKTKKVKEVTQEFVVQNKHKPLWTRD 275

TcDMHsp83-9 -------TKNEEGEEPKVEEVKDDAEEGE---KKKKTKKVKEVTQEFVVQNKHKPLWTRD 275

CfacHsp83-1 --------EGENEEEPKVEEVKDGEED-----K-KKTKKVKEVTKEYEIQNKHKPLWTRD 270

CfacHsp83-2 --------EGENEEEPKVEEVKDGEED-----K-KKTKKVKEVTKEYEIQNKHKPLWTRD 270

LmjHsp83-1 -------KADEDGEEPKVEEVKEGDEG-----KKKKTKKVKEVTKEYEVQNKHKPLWTRD 272

LmjHsp83-2 -------KADEDGEEPKVEEVKEGDEG-----KKKKTKKVKEVTKEYEVQNKHKPLWTRD 272

LmjHsp83-3 -------KADEDGEEPKVEEVKEGDEG-----KKKKTKKVKEVTKEYEVQNKHKPLWTRD 272

LmjHsp83-5 -------KADEDGEEPKVEEVKEGDEG-----KKKKTKKVKEVTKEYEVQNKHKPLWTRD 272

LmjHsp83-6 -------KADEDGEEPKVEEVKEGDEG-----KKKKTKKVKEVTKEYEVQNKHKPLWTRD 272

LmjHsp83-7 -------KADEDGEEPKVEEVKEGDEG-----KKKKTKKVKEVTKEYEVQNKHKPLWTRD 272

LmjHsp83-9 -------KADEDGEEPKVEEVKEGDEG-----KKKKTKKVKEVTKEYEVQNKHKPLWTRD 272

LmjHsp83-10 -------KADEDGEEPKVEEVKEGDEG-----KKKKTKKVKEVTKEYEVQNKHKPLWTRD 272

LmjHsp83-11 -------KADEDGEEPKVEEVKEGDEG-----KKKKTKKVKEVTKEYEVQNKHKPLWTRD 272

LmjHsp83-12 -------KADEDGEEPKVEEVKEGDEG-----KKKKTKKVKEVTKEYEVQNKHKPLWTRD 272

LmjHsp83-13 -------KADEDGEEPKVEEVKEGDEG-----KKKKTKKVKEVTKEYEVQNKHKPLWTRD 272

LmjHsp83-14 -------KADEDGEEPKVEEVKEGDEG-----KKKKTKKVKEVTKEYEVQNKHKPLWTRD 272

LmjHsp83-15 -------KADEDGEEPKVEEVKEGDEG-----KKKKTKKVKEVTKEYEVQNKHKPLWTRD 272

LmjHsp83-16 -------KADEDGEEPKVEEVKEGDEG-----KKKKTKKVKEVTKEYEVQNKHKPLWTRD 272

LmjHsp83-17 -------KADEDGEEPKVEEVKEGDEG-----KKKKTKKVKEVTKEYEVQNKHKPLWTRD 272

LmjHsp83-4 -------KADEDGEEPKVEEVKEGDEG-----KKKKTKKVKEVTKEYEVQNKHKPLWTRD 272

LmjHsp83-8 -------KADEDGEEPKVEEVKEGDEG-----KKKKTKKVKEVTKEYEVQNKHKPLWTRD 272

* . :*

MD

HsHSPC5 PKDVREWQHEEFYRYVAQAHDKPRYTLHYKTDAPLNIRSIFYVPDMKPSM-FDV-SRELG 360

LmjTRAP1 KNDVTNEEHIDFYKFISGSYDSPMFRLHYSIDAPMSVRALLYVPQSHTEK-YGG-G-RMD 404

CfacTRAP1 KNDITNEEHIDFYKFMSGSYDSPMFRLHYSIDAPMSVRALLYVPQSHTEK-YGG-G-RME 405

BsalTRAP1 KNDVSNEEHIDFYKFISGAYDSPLMRLHYSVDAPLTVRALLYIPQSHTEK-YGG-G-RME 321

TbbTRAP1 KNSVTNEEHIDFYKFISGAYDSPMFRLHYAVDAPLSIRALLYVPQSHTEK-YGG-G-RME 398

TbgTRAP1 KNSVTNEEHIDFYKFISGAYDSPMFRLHYAVDAPLSIRALLYVPQSHTEK-YGG-G-RME 398

TcMTRAP1 KNAVSNEEHIDFYKFISGAYDSPMFRLHYVVDAPLSIRALLYVPQSHTEK-YGG-G-RME 394

TcCLBTRAP1 KNAVSNEEHIDFYKFISGAYDSPMFRLHYVVDAPLSIRALLYVPQSHTEK-YGG-G-RME 395

TcDMTRAP1 KNAISNEEHIDFYKFISGAYDSPMFRLHYVVDAPLSIRALLYVPQSHTEK-YGG-G-RME 396

TbbGRP94 PSNVSKEEYEKFYMALSRDYRPPMYYSHFNVEGEVEFSSVLFVPQEVAQENFINN-ENTR 331

TbgGRP94 PSNVSKEEYEKFYMALSRDYRPPMYYSHFNVEGEVEFSSVLFVPQEVAQENFINN-ENTR 331

TcMGRP94 PSEVTEEEYHKFYKSLTHDYRNPMYYSHFNVEGEVEFSSVLFIPQEASQDIFVNN-EDTR 322

TcCLBGRP94 PSEVTEEEYHKFYKSLTHDYRNPMYYSHFNVEGEVEFSSVLFIPQEASQDIFVNN-EDTR 322

TcDMGRP94 PSEVTEEEYHKFYKSLTHDYRNPMYYSHFNVEGEVEFSSVLFIPQEASQDIFVNN-EDTR 322

BsalGRP94 AVEITDAEYNSFFKSLTKDYDDPMFYTHFSAEGEVEFRSILFIPSHSNTNVFDTS--VVQ 353

LmjGRP94 IGNVTEEEYHKFYKAFSGDYRDPLYFSHFKVEGEVDFDSILFVPTTVDPASFSDDNAAPN 363

CfacGRP94 IGNVTEAEYHKFYKSFSGDYRDPLYFNHFRVEGEVEFDSVLFVPATVDVSAFSDDNAQPN 356

HsHSPC4 SKEVEEDEYKAFYKSFSKESDDPMAYIHFTAEGEVTFKSILFVPTSAPRGLFDEYGSKKS 406

HsHSPC1 PDDITNEEYGEFYKSLTNDWEDHLAVKHFSVEGQLEFRALLFVPRRAPFDLFEN--RKKK 480

HsHSPC3 PDDITQEEYGEFYKSLTNDWEDHLAVKHFSVEGQLEFRALLFIPRRAPFDLFEN--KKKK 350

BsalHsp83 PKDVTKEEYGSFYKAISNDWEEPLQYKHFSVEGQLEFRCILFAPKRAPFDMFEP--NKKR 325

TbbHsp83-5 PKDVTKEEYASFYKAISNDWEEQLSTKHFSVEGQLEFRAILFLPKRAPFDMFEP--NKKR 333

TbbHsp83-9 PKDVTKEEYASFYKAISNDWEEQLSTKHFSVEGQLEFRAILFLPKRAPFDMFEP--NKKR 333

TbgHsp83-1 PKDVTKEEYASFYKAISNDWEEQLSTKHFSVEGQLEFRAILFLPKRAPFDMFEP--NKKR 333

TbgHsp83-2 PKDVTKEEYASFYKAISNDWEEQLSTKHFSVEGQLEFRAILFLPKRAPFDMFEP--NKKR 333

TbgHsp83-3 PKDVTKEEYASFYKAISNDWEEQLSTKHFSVEGQLEFRAILFLPKRAPFDMFEP--NKKR 333

TbbHsp83-10 PKDVTKEEYTSFYKAISNDWEEQLSTKHFSVEGQLEFRAILFLPKRAPFDMFEP--NKKR 333

TbbHsp83-1 PKDVTKEEYASFYKAISNDWEEQLSTKHFSVEGQLEFRAILFLPKRAPFDMFEP--NKKR 333

TbbHsp83-2 PKDVTKEEYASFYKAISNDWEEQLSTKHFSVEGQLEFRAILFLPKRAPFDMFEP--NKKR 333

TbbHsp83-4 PKDVTKEEYASFYKAISNDWEEQLSTKHFSVEGQLEFRAILFLPKRAPFDMFEP--NKKR 333

TbbHsp83-6 PKDVTKEEYASFYKAISNDWEEQLSTKHFSVEGQLEFRAILFLPKRAPFDMFEP--NKKR 333

TbbHsp83-7 PKDVTKEEYASFYKAISNDWEEQLSTKHFSVEGQLEFRAILFLPKRAPFDMFEP--NKKR 333

TbbHsp83-8 PKDVTKEEYASFYKAISNDWEEQLSTKHFSVEGQLEFRAILFLPKRAPFDMFEP--NKKR 333

TbbHsp83-3 PKDVTKEEYASFYKAISNDWEEQLSTKHFSVEGQLEFRAILFLPKRAPFDMFEP--NKKR 333

TcMHsp83-2 PKDVTKEEYAAFYKAISNDWEEPLSTKHFSVEGQLEFRAILFVPKRAPFDMFEP--NKKR 333

TcCLBHsp83-2 PKDVTKEEYAAFYKAISNDWEEPLSTKHFSVEGQLEFRAILFVPKRAPFDMFEP--SKKR 333

TcDMHsp83-1 PKDVTKEEYAAFYKAISNDWEEPLSTKHFSVEGQLEFRAILFVPKRAPFDMFEP--SKKR 333

TcDMHsp83-4 PKDVTKEEYAAFYKAISNDWEEPLSTKHFSVEGQLEFRAILFVPKRAPFDMFEP--SKKR 333

TcDMHsp83-5 PKDVTKEEYAAFYKAISNDWEEPLSTKHFSVEGQLEFRAILFVPKRAPFDMFEP--SKKR 333

TcDMHsp83-14 PKDVTKEEYAAFYKAISNDWEEPLSTKHFSVEGQLEFRAILFVPKRAPFDMFEP--SKKR 333

TcDMHsp83-15 PKDVTKEEYAAFYKAISNDWEEPLSTKHFSVEGQLEFRAILFVPKRAPFDMFEP--SKKR 333

TcDMHsp83-16 PKDVTKEEYAAFYKAISNDWEEPLSTKHFSVEGQLEFRAILFVPKRAPFDMFEP--SKKR 333

TcDMHsp83-9 PKDVTKEEYAAFYKAISNDWEEPLSTKHFSVEGQLEFRAILFVPKRAPFDMFEP--SKKR 333

CfacHsp83-1 PKDVTKEEYAAFYKAISNDWEDPAATKHFSVEGQLEFRSILFVPKRAPFDMFEP--NKKR 328

CfacHsp83-2 PKDVTKEEYAAFYKAISNDWEDPAATKHFSVEGQLEFRSILFVPKRAPFDMFEP--NKKR 328

LmjHsp83-1 PKDVTKEEYAAFYKAISNDWEDPAATKHFSVEGQLEFRSIMFVPKRAPFDMFEP--NKKR 330

LmjHsp83-2 PKDVTKEEYAAFYKAISNDWEDPAATKHFSVEGQLEFRSIMFVPKRAPFDMFEP--NKKR 330

LmjHsp83-3 PKDVTKEEYAAFYKAISNDWEDPAATKHFSVEGQLEFRSIMFVPKRAPFDMFEP--NKKR 330

LmjHsp83-5 PKDVTKEEYAAFYKAISNDWEDPAATKHFSVEGQLEFRSIMFVPKRAPFDMFEP--NKKR 330

LmjHsp83-6 PKDVTKEEYAAFYKAISNDWEDPAATKHFSVEGQLEFRSIMFVPKRAPFDMFEP--NKKR 330

LmjHsp83-7 PKDVTKEEYAAFYKAISNDWEDPAATKHFSVEGQLEFRSIMFVPKRAPFDMFEP--NKKR 330

LmjHsp83-9 PKDVTKEEYAAFYKAISNDWEDPAATKHFSVEGQLEFRSIMFVPKRAPFDMFEP--NKKR 330

LmjHsp83-10 PKDVTKEEYAAFYKAISNDWEDPAATKHFSVEGQLEFRSIMFVPKRAPFDMFEP--NKKR 330

LmjHsp83-11 PKDVTKEEYAAFYKAISNDWEDPAATKHFSVEGQLEFRSIMFVPKRAPFDMFEP--NKKR 330

LmjHsp83-12 PKDVTKEEYAAFYKAISNDWEDPAATKHFSVEGQLEFRSIMFVPKRAPFDMFEP--NKKR 330

LmjHsp83-13 PKDVTKEEYAAFYKAISNDWEDPAATKHFSVEGQLEFRSIMFVPKRAPFDMFEP--NKKR 330

LmjHsp83-14 PKDVTKEEYAAFYKAISNDWEDPAATKHFSVEGQLEFRSIMFVPKRAPFDMFEP--NKKR 330

LmjHsp83-15 PKDVTKEEYAAFYKAISNDWEDPAATKHFSVEGQLEFRSIMFVPKRAPFDMFEP--NKKR 330

LmjHsp83-16 PKDVTKEEYAAFYKAISNDWEDPAATKHFSVEGQLEFRSIMFVPKRAPFDMFEP--NKKR 330

LmjHsp83-17 PKDVTKEEYAAFYKAISNDWEDPAATKHFSVEGQLEFRSIMFVPKRAPFDMFEP--NKKR 330

LmjHsp83-4 PKDVTKEEYAAFYKAISNDWEDPAATKHFSVEGQLEFRSIMFVPKRAPFDMFEP--NKKR 330

LmjHsp83-8 PKDVTKEEYAAFYKAISNDWEDPAATKHFSVEGQLEFRSIMFVPKRAPFDMFEP--NKKR 330

: . :: *: .: *: :. : . .::: * :

MD

HsHSPC5 SSVALYSRKVLIQTKATDILPKWLRFIRGVVDSEDIPLNLSRELLQESALIRKLRDVLQQ 420

LmjTRAP1 AGVNLYSRRVLIQSKAKGLLPDWLRFIKGAVDSESIPLNVSREHTQDGSMMRRLSTILTK 464

CfacTRAP1 SGVSLYSRRVLIQSKAKGLLPDWLRFIKGAVDSESIPLNVSREHTQDGGMMRRLSTILTK 465

BsalTRAP1 SGVSLYCRRVLIQSKAKNLLPEWLRFIKGAIDCENIPLNISREHTQDGGMMRRLSTVITK 381

TbbTRAP1 SGVNLYCRRVLIQSKAKGILPEWLRFIKGAVDTESIPLNVSREHTQDGSMMRRLSTVLTK 458

TbgTRAP1 SGVNLYCRRVLIQSKAKGILPEWLRFIKGAVDTESIPLNVSREHTQDGSMMRRLSTVLTK 458

TcMTRAP1 SGVNLYCRRVLIQSKAKGVLPEWLRFIKGAVDSESIPLNVSREHTQDGSMMRRLSTVLTK 454

TcCLBTRAP1 SGVNLYCRRVLIQSKAKGVLPEWLRFIKGAVDSESIPLNVSREHTQDGSMMRRLSTVLTK 455

TcDMTRAP1 SGVNLYCRRVLIQSKAKGVLPEWLRFIKGAVDSESIPLNVSREHTQDGSMMRRLSTVLTK 456

TbbGRP94 DNIKLYVRRIFITDEFRELLPRYLNFVKGVVDSNDLPLNVSREVLQESRILRVIKKKLVR 391

TbgGRP94 DNIKLYVRRIFITDEFRELLPRYLNFVKGVVDSNDLPLNVSREVLQESRILRVIKKKLVR 391

TcMGRP94 DNIKLYVRRIFITDEFRQLLPRYLSFVKGIVDSNDLPLNVSREVLQESRILRVIKKKLVR 382

TcCLBGRP94 DNIKLYVRRIFITDEFRQLLPRYLSFVRGIVDSNDLPLNVSREVLQESRILRVIKKKLVR 382

TcDMGRP94 DNIKLYVRRIFITDEFRQLLPRYLSFVRGIVDSNDLPLNVSREVLQESRILRVIKKKLVR 382

BsalGRP94 ANIRLYVRRVFITDDFRDLLPRYLNFIKGVVDSDDLPLNVSREVLQESRILRVIKKKLVR 413

LmjGRP94 TNIKLYVRRVFITDEFRDLLPRYLNFVKGIVDSNDLPLNVSREVLQESRILRVIKKKLVR 423

CfacGRP94 TNIKLYVRRVFITDEFRDLLPRYLNFVKGIVDSNDLPLNVSREVLQESRILRVIKKKLVR 416

HsHSPC4 DYIKLYVRRVFITDDFHDMMPKYLNFVKGVVDSDDLPLNVSRETLQQHKLLKVIRKKLVR 466

HsHSPC1 NNIKLYVRRVFIMDNCEELIPEYLNFIRGVVDSEDLPLNISREMLQQSKILKVIRKNLVK 540

HsHSPC3 NNIKLYVRRVFIMDSCDELIPEYLNFIRGVVDSEDLPLNISREMLQQSKILKVIRKNIVK 410

BsalHsp83 NNIKLYVRRVFIMDNCEDLCPEWLGFLKGVVDSEDLPLNISRENLQQNKVLKVIKKNIVK 385

TbbHsp83-5 NNIKLYVRRVFIMDNCEDLCPEWLGFLRGVVDSEDLPLNISRENLQQNKILKVIRKNIVK 393

TbbHsp83-9 NNIKLYVRRVFIMDNCEDLCPEWLGFLRGVVDSEDLPLNISRENLQQNKILKVIRKNIVK 393

TbgHsp83-1 NNIKLYVRRVFIMDNCEDLCPEWLGFLRGVVDSEDLPLNISRENLQQNKILKVIRKNIVK 393

TbgHsp83-2 NNIKLYVRRVFIMDNCEDLCPEWLGFLRGVVDSEDLPLNISRENLQQNKILKVIRKNIVK 393

TbgHsp83-3 NNIKLYVRRVFIMDNCEDLCPEWLGFLRGVVDSEDLPLNISRENLQQNKILKVIRKNIVK 393

TbbHsp83-10 NNIKLYVRRVFIMDNCEDLCPEWLGFLRGVVDSEDLPLNISRENLQQNKILKVIRKNIVK 393

TbbHsp83-1 NNIKLYVRRVFIMDNCEDLCPEWLGFLRGVVDSEDLPLNISRENLQQNKILKVIRKNIVK 393

TbbHsp83-2 NNIKLYVRRVFIMDNCEDLCPEWLGFLRGVVDSEDLPLNISRENLQQNKILKVIRKNIVK 393

TbbHsp83-4 NNIKLYVRRVFIMDNCEDLCPEWLGFLRGVVDSEDLPLNISRENLQQNKILKVIRKNIVK 393

TbbHsp83-6 NNIKLYVRRVFIMDNCEDLCPEWLGFLRGVVDSEDLPLNISRENLQQNKILKVIRKNIVK 393

TbbHsp83-7 NNIKLYVRRVFIMDNCEDLCPEWLGFLRGVVDSEDLPLNISRENLQQNKILKVIRKNIVK 393

TbbHsp83-8 NNIKLYVRRVFIMDNCEDLCPEWLGFLRGVVDSEDLPLNISRENLQQNKILKVIRKNIVK 393

TbbHsp83-3 NNIKLYVRRVFIMDNCEDLCPDWLGFLRGVVDSEDLPLNISRENLQQNKILKVIRKNIVK 393

TcMHsp83-2 NNIKLYVRRVFIMDNCEDLCPEWLAFVRGVVDSEDLPLNISRENLQQNKILKVIRKNIVK 393

TcCLBHsp83-2 NNIKLYVRRVFIMDNCEDLCPEWLAFVRGVVDSEDLPLNISRENLQQNKILKVIRKNIVK 393

TcDMHsp83-1 NNIKLYVRRVFIMDNCEDLCPEWLAFVRGVVDSEDLPLNISRENLQQNKILKVIRKNIVK 393

TcDMHsp83-4 NNIKLYVRRVFIMDNCEDLCPEWLAFVRGVVDSEDLPLNISRENLQQNKILKVIRKNIVK 393

TcDMHsp83-5 NNIKLYVRRVFIMDNCEDLCPEWLAFVRGVVDSEDLPLNISRENLQQNKILKVIRKNIVK 393

TcDMHsp83-14 NNIKLYVRRVFIMDNCEDLCPEWLAFVRGVVDSEDLPLNISRENLQQNKILKVIRKNIVK 393

TcDMHsp83-15 NNIKLYVRRVFIMDNCEDLCPEWLAFVRGVVDSEDLPLNISRENLQQNKILKVIRKNIVK 393

TcDMHsp83-16 NNIKLYVRRVFIMDNCEDLCPEWLAFVRGVVDSEDLPLNISRENLQQNKILKVIRKNIVK 393

TcDMHsp83-9 NNIKLYVRRVFIMDNCEDLCPEWLAFVRGVVDSEDLPLNISRENLQQNKILKVIRKNIVK 393

CfacHsp83-1 NNIKLYVRRVFIMDNCEDLCPDWLGFVKGVVDSEDLPLNISRENLQQNKILKVIRKNIVK 388

CfacHsp83-2 NNIKLYVRRVFIMDNCEDLCPDWLGFVKGVVDSEDLPLNISRENLQQNKILKVIRKNIVK 388

LmjHsp83-1 NNIKLYVRRVFIMDNCEDLCPDWLGFVKGVVDSEDLPLNISRENLQQNKILKVIRKNIVK 390

LmjHsp83-2 NNIKLYVRRVFIMDNCEDLCPDWLGFVKGVVDSEDLPLNISRENLQQNKILKVIRKNIVK 390

LmjHsp83-3 NNIKLYVRRVFIMDNCEDLCPDWLGFVKGVVDSEDLPLNISRENLQQNKILKVIRKNIVK 390

LmjHsp83-5 NNIKLYVRRVFIMDNCEDLCPDWLGFVKGVVDSEDLPLNISRENLQQNKILKVIRKNIVK 390

LmjHsp83-6 NNIKLYVRRVFIMDNCEDLCPDWLGFVKGVVDSEDLPLNISRENLQQNKILKVIRKNIVK 390

LmjHsp83-7 NNIKLYVRRVFIMDNCEDLCPDWLGFVKGVVDSEDLPLNISRENLQQNKILKVIRKNIVK 390

LmjHsp83-9 NNIKLYVRRVFIMDNCEDLCPDWLGFVKGVVDSEDLPLNISRENLQQNKILKVIRKNIVK 390

LmjHsp83-10 NNIKLYVRRVFIMDNCEDLCPDWLGFVKGVVDSEDLPLNISRENLQQNKILKVIRKNIVK 390

LmjHsp83-11 NNIKLYVRRVFIMDNCEDLCPDWLGFVKGVVDSEDLPLNISRENLQQNKILKVIRKNIVK 390

LmjHsp83-12 NNIKLYVRRVFIMDNCEDLCPDWLGFVKGVVDSEDLPLNISRENLQQNKILKVIRKNIVK 390

LmjHsp83-13 NNIKLYVRRVFIMDNCEDLCPDWLGFVKGVVDSEDLPLNISRENLQQNKILKVIRKNIVK 390

LmjHsp83-14 NNIKLYVRRVFIMDNCEDLCPDWLGFVKGVVDSEDLPLNISRENLQQNKILKVIRKNIVK 390

LmjHsp83-15 NNIKLYVRRVFIMDNCEDLCPDWLGFVKGVVDSEDLPLNISRENLQQNKILKVIRKNIVK 390

LmjHsp83-16 NNIKLYVRRVFIMDNCEDLCPDWLGFVKGVVDSEDLPLNISRENLQQNKILKVIRKNIVK 390

LmjHsp83-17 NNIKLYVRRVFIMDNCEDLCPDWLGFVKGVVDSEDLPLNISRENLQQNKILKVIRKNIVK 390

LmjHsp83-4 NNIKLYVRRVFIMDNCEDLCPDWLGFVKGVVDSEDLPLNISRENLQQNKILKVIRKNIVK 390

LmjHsp83-8 NNIKLYVRRVFIMDNCEDLCPDWLGFVKGVVDSEDLPLNISRENLQQNKILKVIRKNIVK 390

: ** *:::* . : * :* *::* :* :.:***:*** *: ::: : : :

MD

HsHSPC5 RLIKFFIDQSKKD----------------------------------AEKYAKFFEDYGL 446

LmjTRAP1 RIIRWLEEESKRD----------------------------------RSKYERFIQEYGP 490

CfacTRAP1 RIIRWFEEESKRD----------------------------------RSKYERFIQEYGA 491

BsalTRAP1 RVIRWLEEEAKKD----------------------------------RQAFERFIREFGP 407

TbbTRAP1 RVIRWMEEEAKQD----------------------------------RQKYERFIKEYGP 484

TbgTRAP1 RVIRWMEEEAKQD----------------------------------RQKYERFIKEYGP 484

TcMTRAP1 RIIRWLEEEAKQD----------------------------------RQKYERFIQEYGP 480

TcCLBTRAP1 RIIRWLEEEAKQD----------------------------------RQKYERFIQEYGP 481

TcDMTRAP1 RIIRWLEEEAKQD----------------------------------RQKYERFIQEYGP 482

TbbGRP94 KVLSMFAEIAANDARMKEQGNVSE-----EVNAEVNTTNSTSGSKKKGPLYPKFWAQFGK 446

TbgGRP94 KVLSMFAEIAANDARMKEQGNVSE-----EVNAEVNTTNSTSGSKKKGPLYPKFWAQFGK 446

TcMGRP94 KALSMISEIAEKDARLKESLERENSEAKGEEKDENATEKKSDDVKGKEPLYPQFWAQFGK 442

TcCLBGRP94 KALSMISEIAEKDARLKEGLEKEKSGEDGEAKDENTTEKKSDDDKGKEPLYPKFWAQFGK 442

TcDMGRP94 KALSMISEIAEKDARLKEGLEKEKSGEDGEAKDENTTGKKSGDDKGKEPLYPKFWAQFGK 442

BsalGRP94 KALAMIADIAASDKKLEAAKDDDEAAEA----EEKKDDVTAGNKQLKASTYPKFWEEYGK 469

LmjGRP94 KTLSMFADIAAQDEAIADGKQ-------------VENPALSGHTHLKKPAYTKFWELYGK 470

CfacGRP94 KALTMFSDIAEQDEAIAQGKQ-------------PESLAPTGHTHLTKPTYTKFWELFGK 463

HsHSPC4 KTLDMIKKIADDK-----------------------------------YN-DTFWKEFGT 490

HsHSPC1 KCLELFTELAEDK-----------------------------------ENYKKFYEQFSK 565

HsHSPC3 KCLELFSELAEDK-----------------------------------ENYKKFYEAFSK 435

BsalHsp83 KALELFEEIAENK-----------------------------------EDYLKFYEQFGK 410

TbbHsp83-5 KTLELFEELAENK-----------------------------------EDYKKFYEQFSK 418

TbbHsp83-9 KTLELFEELAENK-----------------------------------EDYKKFYEQFSK 418

TbgHsp83-1 KALELFEELAENK-----------------------------------EDYKKFYEQFSK 418

TbgHsp83-2 KALELFEELAENK-----------------------------------EDYKKFYEQFSK 418

TbgHsp83-3 KALELFEELAENK-----------------------------------EDYKKFYEQFSK 418

TbbHsp83-10 KALELFEELAENK-----------------------------------EDYKKFYEQFSK 418

TbbHsp83-1 KALELFEELAENK-----------------------------------EDYKKFYEQFSK 418

TbbHsp83-2 KALELFEELAENK-----------------------------------EDYKKFYEQFSK 418

TbbHsp83-4 KALELFEELAENK-----------------------------------EDYKKFYEQFSK 418

TbbHsp83-6 KALELFEELAENK-----------------------------------EDYKKFYEQFSK 418

TbbHsp83-7 KALELFEELAENK-----------------------------------EDYKKFYEQFSK 418

TbbHsp83-8 KALELFEELAENK-----------------------------------EDYKKFYEQFSK 418

TbbHsp83-3 KALELFEELAENK-----------------------------------EDYKKFYEQFSK 418

TcMHsp83-2 KALELFEEIAENK-----------------------------------EDYKKFYEQFGK 418

TcCLBHsp83-2 KALELFEEIAENK-----------------------------------EDYKKFYEQFGK 418

TcDMHsp83-1 KALELFEEIAENK-----------------------------------EDYKKFYEQFGK 418

TcDMHsp83-4 KALELFEEIAENK-----------------------------------EDYKKFYEQFGK 418

TcDMHsp83-5 KALELFEEIAENK-----------------------------------EDYKKFYEQFGK 418

TcDMHsp83-14 KALELFEEIAENK-----------------------------------EDYKKFYEQFGK 418

TcDMHsp83-15 KALELFEEIAENK-----------------------------------EDYKKFYEQFGK 418

TcDMHsp83-16 KALELFEEIAENK-----------------------------------EDYKKFYEQFGK 418

TcDMHsp83-9 KALELFEEIAENK-----------------------------------EDYKKFYEQFGK 418

CfacHsp83-1 KCLDLFDELAENK-----------------------------------EDFKQFYEQFGK 413

CfacHsp83-2 KCLDLFDELAENK-----------------------------------EDFKQFYEQFGK 413

LmjHsp83-1 KCLEMFDEVAENK-----------------------------------EDYKQFYEQFGK 415

LmjHsp83-2 KCLEMFDEVAENK-----------------------------------EDYKQFYEQFGK 415

LmjHsp83-3 KCLEMFDEVAENK-----------------------------------EDYKQFYEQFGK 415

LmjHsp83-5 KCLEMFDEVAENK-----------------------------------EDYKQFYEQFGK 415

LmjHsp83-6 KCLEMFDEVAENK-----------------------------------EDYKQFYEQFGK 415

LmjHsp83-7 KCLEMFDEVAENK-----------------------------------EDYKQFYEQFGK 415

LmjHsp83-9 KCLEMFDEVAENK-----------------------------------EDYKQFYEQFGK 415

LmjHsp83-10 KCLEMFDEVAENK-----------------------------------EDYKQFYEQFGK 415

LmjHsp83-11 KCLEMFDEVAENK-----------------------------------EDYKQFYEQFGK 415

LmjHsp83-12 KCLEMFDEVAENK-----------------------------------EDYKQFYEQFGK 415

LmjHsp83-13 KCLEMFDEVAENK-----------------------------------EDYKQFYEQFGK 415

LmjHsp83-14 KCLEMFDEVAENK-----------------------------------EDYKQFYEQFGK 415

LmjHsp83-15 KCLEMFDEVAENK-----------------------------------EDYKQFYEQFGK 415

LmjHsp83-16 KCLEMFDEVAENK-----------------------------------EDYKQFYEQFGK 415

LmjHsp83-17 KCLEMFDEVAENK-----------------------------------EDYKQFYEQFGK 415

LmjHsp83-4 KCLEMFDEVAENK-----------------------------------EDYKQFYEQFGK 415

LmjHsp83-8 KCLEMFDEVAENK-----------------------------------EDYKQFYEQFGK 415

: : : . : . * :.

MD

HsHSPC5 FMREGIVTATEQEVKEDIAKLLRYESSALP-SGQLTSLSEYASRMRAGTRNIYYLCAPNR 505

LmjTRAP1 FLKEGVCTD--QVHKMELAKLLRFQTTKSDIDYPYVSLDNYRDRMQPNQSHIYYLNLPSK 548

CfacTRAP1 FLKEGICTD--QVHKMDLAKLLRFETTKSDVDYPFVSLDNYRDRMQPNQSHIYYLNSPSK 549

BsalTRAP1 FLKEGCCTD--QVHKMDLAKLLRFETTKTDLDYPLVSLDEYRDRMKPNQTHIYYLNAPTK 465

TbbTRAP1 FLKEGVCTD--QVHKMELAKLLRFETTKSDIDYPYVSLDEYRDRMVANQTHIYYINAPSK 542

TbgTRAP1 FLKEGVCTD--QVHKMELAKLLRFETTKSDIDYPYVSLDEYRDRMVANQTHIYYINAPSK 542

TcMTRAP1 FLKEGVCTD--QVHKMELAKLLRFETTKSDIDYPLVSLDEYRDRMLANQTHIYYINAPSK 538

TcCLBTRAP1 FLKEGVCTD--QVHKMELAKLLRFETTKSDIDYPLVSLEEYRDRMLANQTHIYYINAPSK 539

TcDMTRAP1 FLKEGVCTD--QVHKMELAKLLRFETTKSDIDYPLVSLEEYRDRMLANQTHIYYINAPSK 540

TbbGRP94 HLRLGILED--ANNRGRLAKLLRYVSSKSN--GTLVSFQEYIDRMQPNQKGIYYMTGDSV 502

TbgGRP94 HLRLGILED--ANNRGRLAKLLRYVSSKSN--GTLVSFQEYIDRMQPNQKGIYYMTGDSV 502

TcMGRP94 HIRLGILED--ANNRGRLAKLLRYTSTKSN--GTLVSLQEYTDRMKPEQKNIYFLTGESV 498

TcCLBGRP94 HIRLGILED--ANNRGRLAKLLRYTSTKSN--GTLVSLQEYTDRMKPEQKHIYFLTGDSV 498

TcDMGRP94 HIRLGILED--ANNRGRLAKLLRYTSTKSN--GTLVSLQEYTDRMKPEQKHIYFLTGESV 498

BsalGRP94 NIRLGMIED--GSNRARLTKLLRYKSSKSD--NKLISLQDYVDRMPESQKDIYYVSAESI 525

LmjGRP94 HLRLGVMLD--SNNRNRLTKLFRYKSSRSE--SEYISLQTYVDRMKKGQKGIYYLSGDSV 526

CfacGRP94 HLRLGVMLD--SNNRNRLTKLFRYKSSKSD--DAYISLQTYVDRMKKGQKGIYYISGDSV 519

HsHSPC4 NIKLGVIED--HSNRTRLAKLLRFQSSHHP--TDITSLDQYVERMKEKQDKIYFMAGSSR 546

HsHSPC1 NIKLGIHED--SQNRKKLSELLRYYTSASG--DEMVSLKDYCTRMKENQKHIYYITGETK 621

HsHSPC3 NLKLGIHED--STNRRRLSELLRYHTSQSG--DEMTSLSEYVSRMKETQKSIYYITGESK 491

BsalHsp83 NLKLGIHED--QTNRKKLLELLRYYSTQAS--EEPTTLKDYVTRMKPEQKTIYYITGDSK 466

TbbHsp83-5 NVKLGIHED--STNRKKLMELLRFHSSESG--EEMTTLKDYVTRMKDGQKCIYYVTGDSK 474

TbbHsp83-9 NVKLGIHED--STNRKKLMELLRFHSSESG--EEMTTLKDYVTRMKDGQKCIYYVTGDSK 474

TbgHsp83-1 NVKLGIHED--STNRKKLMELLRFHSSESG--EEMTTLKDYVTRMKEGQKCIYYVTGDSK 474

TbgHsp83-2 NVKLGIHED--STNRKKLMELLRFHSSESG--EEMTTLKDYVTRMKEGQKCIYYVTGDSK 474

TbgHsp83-3 NVKLGIHED--STNRKKLMELLRFHSSESG--EEMTTLKDYVTRMKEGQKCIYYVTGDSK 474

TbbHsp83-10 NVKLGIHED--STNRKKLMELLRFHSSESG--EEMTTLKDYVTRMKDGQKCIYYVTGDSK 474

TbbHsp83-1 NVKLGIHED--STNRKKLMELLRFHSSESG--EEMTTLKDYVTRMKDGQKCIYYVTGDSK 474

TbbHsp83-2 NVKLGIHED--STNRKKLMELLRFHSSESG--EEMTTLKDYVTRMKDGQKCIYYVTGDSK 474

TbbHsp83-4 NVKLGIHED--STNRKKLMELLRFHSSESG--EEMTTLKDYVTRMKDGQKCIYYVTGDSK 474

TbbHsp83-6 NVKLGIHED--STNRKKLMELLRFHSSESG--EEMTTLKDYVTRMKDGQKCIYYVTGDSK 474

TbbHsp83-7 NVKLGIHED--STNRKKLMELLRFHSSESG--EEMTTLKDYVTRMKDGQKCIYYVTGDSK 474

TbbHsp83-8 NVKLGIHED--STNRKKLMELLRFHSSESG--EEMTTLKDYVTRMKDGQKCIYYVTGDSK 474

TbbHsp83-3 NVKLGIHED--STNRKKLMELLRFHSSESG--EEMTTLKDYVTRMKDGQKCIYYVTGDSK 474

TcMHsp83-2 NVKLGIHED--SANRKKLMELLRFHSSESG--EDMTTLKDYVTRMKEGQKCIYYVTGDSK 474

TcCLBHsp83-2 NVKLGIHED--SANRKKLMELLRFHSSESG--EDMTTLKDYVTRMKEGQKCIYYVTGDSK 474

TcDMHsp83-1 NVKLGIHED--SANRKKLMELLRFHSSESG--EDMTTLKDYVTRMKEGQKCIYYVTGDSK 474

TcDMHsp83-4 NVKLGIHED--SANRKKLMELLRFHSSESG--EDMTTLKDYVTRMKEGQKCIYYVTGDSK 474

TcDMHsp83-5 NVKLGIHED--SANRKKLMELLRFHSSESG--EDMTTLKDYVTRMKEGQKCIYYVTGDSK 474

TcDMHsp83-14 NVKLGIHED--SANRKKLMELLRFHSSESG--EDMTTLKDYVTRMKEGQKCIYYVTGDSK 474

TcDMHsp83-15 NVKLGIHED--SANRKKLMELLRFHSSESG--EDMTTLKDYVTRMKEGQKCIYYVTGDSK 474

TcDMHsp83-16 NVKLGIHED--SANRKKLMELLRFHSSESG--EDMTTLKDYVTRMKEGQKCIYYVTGDSK 474

TcDMHsp83-9 NVKLGIHED--SANRKKLMELLRFHSSESG--EDMTTLKDYVTRMKEGQKCIYYVTGDSK 474

CfacHsp83-1 NLKLGIHED--TANRKKLMELLRYASTESG--EELTTLKDYVTRMKPEQKSIYYITGDSK 469

CfacHsp83-2 NLKLGIHED--TANRKKLMELLRYASTESG--EELTTLKDYVTRMKPEQKSIYYITGDSK 469

LmjHsp83-1 NIKLGIHED--TANRKKLMELLRFYSTESG--EEMTTLKDYVTRMKAGQKSIYYITGDSK 471

LmjHsp83-2 NIKLGIHED--TANRKKLMELLRFYSTESG--EEMTTLKDYVTRMKAGQKSIYYITGDSK 471

LmjHsp83-3 NIKLGIHED--TANRKKLMELLRFYSTESG--EEMTTLKDYVTRMKAGQKSIYYITGDSK 471

LmjHsp83-5 NIKLGIHED--TANRKKLMELLRFYSTESG--EEMTTLKDYVTRMKAGQKSIYYITGDSK 471

LmjHsp83-6 NIKLGIHED--TANRKKLMELLRFYSTESG--EEMTTLKDYVTRMKAGQKSIYYITGDSK 471

LmjHsp83-7 NIKLGIHED--TANRKKLMELLRFYSTESG--EEMTTLKDYVTRMKAGQKSIYYITGDSK 471

LmjHsp83-9 NIKLGIHED--TANRKKLMELLRFYSTESG--EEMTTLKDYVTRMKAGQKSIYYITGDSK 471

LmjHsp83-10 NIKLGIHED--TANRKKLMELLRFYSTESG--EEMTTLKDYVTRMKAGQKSIYYITGDSK 471

LmjHsp83-11 NIKLGIHED--TANRKKLMELLRFYSTESG--EEMTTLKDYVTRMKAGQKSIYYITGDSK 471

LmjHsp83-12 NIKLGIHED--TANRKKLMELLRFYSTESG--EEMTTLKDYVTRMKAGQKSIYYITGDSK 471

LmjHsp83-13 NIKLGIHED--TANRKKLMELLRFYSTESG--EEMTTLKDYVTRMKAGQKSIYYITGDSK 471

LmjHsp83-14 NIKLGIHED--TANRKKLMELLRFYSTESG--EEMTTLKDYVTRMKAGQKSIYYITGDSK 471

LmjHsp83-15 NIKLGIHED--TANRKKLMELLRFYSTESG--EEMTTLKDYVTRMKAGQKSIYYITGDSK 471

LmjHsp83-16 NIKLGIHED--TANRKKLMELLRFYSTESG--EEMTTLKDYVTRMKAGQKSIYYITGDSK 471

LmjHsp83-17 NIKLGIHED--TANRKKLMELLRFYSTESG--EEMTTLKDYVTRMKAGQKSIYYITGDSK 471

LmjHsp83-4 NIKLGIHED--TANRKKLMELLRFYSTESG--EEMTTLKDYVTRMKAGQKSIYYITGDSK 471

LmjHsp83-8 NIKLGIHED--TANRKKLMELLRFYSTESG--EEMTTLKDYVTRMKAGQKSIYYITGDSK 471

:: * : : :*:*: :: ::. * ** **:: .

MD

HsHSPC5 HLAEHSPYYEAMKKKDTEVLFCFEQFDELTLLHLREFDKKKLISVETD-IVVDHYKEEK- 563

LmjTRAP1 EMALQSPYYEQYKEHDLEVLICTEPIDDFVMQHLDTYAKHKLQNIEMFDANLDGYVQHKK 608

CfacTRAP1 DMAMQSPYYEQYKEHGLEVLICTEPMDDFVMQHLDTYAKHKLQNIEMFDANLDGYVQHKK 609

BsalTRAP1 EMALMSPYYEQYKEHGLEVLICTEPIDDFVMQHVDTYSKHKLQNIEMFDAQYDGNVQHTK 525

TbbTRAP1 EMALESPYYEQYKEHDLEVLVCTEPIDDFVMQHLDTYAKHKLQNIELFDASLDGSVQNKL 602

TbgTRAP1 EMALESPYYEQYKEHDLEVLVCTEPIDDFVMQHLDTYAKHKLQNIELFDASLDGSVQNKL 602

TcMTRAP1 EMALQSPYYEQYKEHELEVLVCTEPIDDFVMQHLDTYAKHKLQNIEMFDASLDGSVQHKK 598

TcCLBTRAP1 EMALQSPYYEQYKEHELEVLVCTEPIDDFVMQHLDTYAKHKLQNIEMFDASLDGSVQHKK 599

TcDMTRAP1 EMALQSPYYEQYKEHEIEVLVCTEPIDDFVMQHLDTYAKHKLQNIEMFDASLDGSVQHKK 600

TbbGRP94 EKMMQSPHMEEPKMRGVEVLLMTDAIDEYVVGQVHDFANKKLINIATDSAQLDDVTDKQK 562

TbgGRP94 EKMMQSPHMEEPKMRGVEVLLMTDAIDEYVVGQVHDFANKKLINIATDSAQLDDVTDKQK 562

TcMGRP94 KKMRQSPHIEEALERDVEVLFMTDAIDEYVVSQVQDFGNKRLINLAKDNARLDEPTERDK 558

TcCLBGRP94 KKMRQSPHIEEALERDVEVLFMTDAIDEYVVSQVQDFGNKRLINLAKDNARLDEPTERDK 558

TcDMGRP94 KKMRQSPHIEEALERDVEVLFMTDAIDEYVVSQVQDFGNKRLINLAKDNARLDEPTERDK 558

BsalGRP94 EKIKQLPVLEDATNRNLEVLFMTDAIDEYVVGHVTDFAGKKLVNLAKEGVKFEDESKREK 585

LmjGRP94 ARIKKSPVLEDAVNHDVEVIFMTDAIDEYVVSQLTDFAGKKLINLAKEGVQFEESDARQR 586

CfacGRP94 ARIQKSPVLEDAVNHDVEVIFMTDAIDEYVVAQVTDFAGKKLINLAKEGVQFDETDARQR 579

HsHSPC4 KEAESSPFVERLLKKGYEVIYLTEPVDEYCIQALPEFDGKRFQNVAKEGVKFDESEKTKE 606

HsHSPC1 DQVANSAFVERLRKHGLEVIYMIEPIDEYCVQQLKEFEGKTLVSVTKEGLELPEDEEEKK 681

HsHSPC3 EQVANSAFVERVRKRGFEVVYMTEPIDEYCVQQLKEFDGKSLVSVTKEGLELPEDEEEKK 551

BsalHsp83 KKLESSPFIEEAKRRGVEVLFMVDPIDEYVMQQVKDFEDLKFVCLTKEGVKFEESEDEKK 526

TbbHsp83-5 KKLETSPFIEQAKRRGMEVLFMTDPIDEYVMQQVKDFEDKKFACLTKEGVHFEETEEEKK 534

TbbHsp83-9 KKLETSPFIEQAKRRGMEVLFMTDPIDEYVMQQVKDFEDKKFACLTKEGVHFEETEEEKK 534

TbgHsp83-1 KKLETSPFIEQAKRRGMEVLFMTDPIDEYVMQQVKDFEDKKFACLTKEGVHFEETEEEKK 534

TbgHsp83-2 KKLETSPFIEQAKRRGMEVLFMTDPIDEYVMQQVKDFEDKKFACLTKEGVHFEETEEEKK 534

TbgHsp83-3 KKLETSPFIEQAKRRGMEVLFMTDPIDEYVMQQVKDFEDKKFACLTKEGVHFEETEEEKK 534

TbbHsp83-10 KKLETSPFIEQAKRRGMEVLFMTDPIDEYVMQQVKDFEDKKFACLTKEGVHFEETEEEKK 534

TbbHsp83-1 KKLETSPFIEQAKRRGMEVLFMTDPIDEYVMQQVKDFEDKKFACLTKEGVHFEETEEEKK 534

TbbHsp83-2 KKLETSPFIEQAKRRGMEVLFMTDPIDEYVMQQVKDFEDKKFACLTKEGVHFEETEEEKK 534

TbbHsp83-4 KKLETSPFIEQAKRRGMEVLFMTDPIDEYVMQQVKDFEDKKFACLTKEGVHFEETEEEKK 534

TbbHsp83-6 KKLETSPFIEQAKRRGMEVLFMTDPIDEYVMQQVKDFEDKKFACLTKEGVHFEETEEEKK 534

TbbHsp83-7 KKLETSPFIEQAKRRGMEVLFMTDPIDEYVMQQVKDFEDKKFACLTKEGVHFEETEEEKK 534

TbbHsp83-8 KKLETSPFIEQAKRRGMEVLFMTDPIDEYVMQQVKDFEDKKFACLTKEGVHFEETEEEKK 534

TbbHsp83-3 KKLETSPFIEQAKRRGMEVLFMTDPIDEYVMQQVKDFEDKKFACLTKEGVHFEETEEEKK 534

TcMHsp83-2 KKLETSPFIEQARRRGFEVLFMTEPIDEYVMQQVKDFEDKKFACLTKEGVHFEETEEEKK 534

TcCLBHsp83-2 KKLETSPFIEQARRRGFEVLFMTEPIDEYVMQQVKDFEDKKFACLTKEGVHFEETEEEKK 534

TcDMHsp83-1 KKLETSPFIEQARRRGFEVLFMTEPIDEYVMQQVKDFEDKKFACLTKEGVHFEETEEEKK 534

TcDMHsp83-4 KKLETSPFIEQARRRGFEVLFMTEPIDEYVMQQVKDFEDKKFACLTKEGVHFEETEEEKK 534

TcDMHsp83-5 KKLETSPFIEQARRRGFEVLFMTEPIDEYVMQQVKDFEDKKFACLTKEGVHFEETEEEKK 534

TcDMHsp83-14 KKLETSPFIEQARRRGFEVLFMTEPIDEYVMQQVKDFEDKKFACLTKEGVHFEETEEEKK 534

TcDMHsp83-15 KKLETSPFIEQARRRGFEVLFMTEPIDEYVMQQVKDFEDKKFACLTKEGVHFEETEEEKK 534

TcDMHsp83-16 KKLETSPFIEQARRRGFEVLFMTEPIDEYVMQQVKDFEDKKFACLTKEGVHFEETEEEKK 534

TcDMHsp83-9 KKLETSPFIEQARRRGFEVLFMTEPIDEYVMQQVKDFEDKKFACLTKEGVHFEETEEEKK 534

CfacHsp83-1 KKLESSPFIEEAKRRGIEVLFMTEPIDEYVMQQVKDFEDKKFACLTKEGVHFEDSEDEKK 529

CfacHsp83-2 KKLESSPFIEEAKRRGIEVLFMTEPIDEYVMQQVKDFEDKKFACLTKEGVHFEDSEDEKK 529

LmjHsp83-1 KKLETSPFIEQARRRGLEVLFMTEPIDEYVMQQVKDFEDKKFACLTKEGVHFEESEEEKQ 531

LmjHsp83-2 KKLETSPFIEQARRRGLEVLFMTEPIDEYVMQQVKDFEDKKFACLTKEGVHFEESEEEKQ 531

LmjHsp83-3 KKLETSPFIEQARRRGLEVLFMTEPIDEYVMQQVKDFEDKKFACLTKEGVHFEESEEEKQ 531

LmjHsp83-5 KKLETSPFIEQARRRGLEVLFMTEPIDEYVMQQVKDFEDKKFACLTKEGVHFEESEEEKQ 531

LmjHsp83-6 KKLETSPFIEQARRRGLEVLFMTEPIDEYVMQQVKDFEDKKFACLTKEGVHFEESEEEKQ 531

LmjHsp83-7 KKLETSPFIEQARRRGLEVLFMTEPIDEYVMQQVKDFEDKKFACLTKEGVHFEESEEEKQ 531

LmjHsp83-9 KKLETSPFIEQARRRGLEVLFMTEPIDEYVMQQVKDFEDKKFACLTKEGVHFEESEEEKQ 531

LmjHsp83-10 KKLETSPFIEQARRRGLEVLFMTEPIDEYVMQQVKDFEDKKFACLTKEGVHFEESEEEKQ 531

LmjHsp83-11 KKLETSPFIEQARRRGLEVLFMTEPIDEYVMQQVKDFEDKKFACLTKEGVHFEESEEEKQ 531

LmjHsp83-12 KKLETSPFIEQARRRGLEVLFMTEPIDEYVMQQVKDFEDKKFACLTKEGVHFEESEEEKQ 531

LmjHsp83-13 KKLETSPFIEQARRRGLEVLFMTEPIDEYVMQQVKDFEDKKFACLTKEGVHFEESEEEKQ 531

LmjHsp83-14 KKLETSPFIEQARRRGLEVLFMTEPIDEYVMQQVKDFEDKKFACLTKEGVHFEESEEEKQ 531

LmjHsp83-15 KKLETSPFIEQARRRGLEVLFMTEPIDEYVMQQVKDFEDKKFACLTKEGVHFEESEEEKQ 531

LmjHsp83-16 KKLETSPFIEQARRRGLEVLFMTEPIDEYVMQQVKDFEDKKFACLTKEGVHFEESEEEKQ 531

LmjHsp83-17 KKLETSPFIEQARRRGLEVLFMTEPIDEYVMQQVKDFEDKKFACLTKEGVHFEESEEEKQ 531

LmjHsp83-4 KKLETSPFIEQARRRGLEVLFMTEPIDEYVMQQVKDFEDKKFACLTKEGVHFEESEEEKQ 531

LmjHsp83-8 KKLETSPFIEQARRRGLEVLFMTEPIDEYVMQQVKDFEDKKFACLTKEGVHFEESEEEKQ 531

* : **: : .*: : : : : :

MD

HsHSPC5 -F----EDRSPAAECLSEKETEELMAWMRN-VLGSRVTNVKVTLRLDTHPAMVTVLEM-- 615

LmjTRAP1 KLEGDKNDDVAVKKQLNDVQVKALSDFISK-RLVGRVGVVKSTDRLRDSPAVLADHEA-- 665

CfacTRAP1 KMEGDKEEDVSVKKQLSEVQVKALADFMLK-RLVGRIGVVKATSRLRDSPAVLADHES-- 666

BsalTRAP1 KIEGEKKEDVVVKKQLTEVQVKALSDFMSK-RLVGRVGVVKATERLRDSPAVLADHES-- 582

TbbTRAP1 KLEGDK-GEVKVEKQLTEAQVKALSDFISK-RLVGRVGVVKSTTRLRDSPAVIADHES-- 658

TbgTRAP1 KLEGDK-GEVKVEKQLTEAQVKALSDFISK-RLVGRVGVVKSTTRLRDSPAVIADHES-- 658

TcMTRAP1 KLEGEK-EDVKVEKQLTEAQVKGLSDFIAK-RLVGRVGVVKSTNRLRDSPAVIADHES-- 654

TcCLBTRAP1 KLEGEK-EDVKVEKQLTEAQVKGLSDFIAK-RLVGRVGVVKSTSRLRDSPAVIADHES-- 655

TcDMTRAP1 KLEGEK-EDVKVEKQLTEAQVKGLSDFIAK-RLVGRVGVVKSTSRLRDSPAVIADHES-- 656

TbbGRP94 AIEKKRN-----------EKFRPLTDALTRVFKGNRVRKVILTKRKTSEPFILSSQENEM 611

TbgGRP94 AIEKKRN-----------EKFRPLTDALTRVFKGNRVRKVILTKRKTSEPFILSSQENEM 611

TcMGRP94 SIEKERN-----------KKYEPLTERLMTLFGKSQVRKVILTKRQSSEAFILSTQENDM 607

TcCLBGRP94 AIEKERK-----------KKYEPLTERLLTLFGKSQVRKVILTRRQSSEAFILSTQENDM 607

TcDMGRP94 AIEKERN-----------KKYEPLTERLLSLFGKSQVRKVILTRRQSSEAFILSTQENDM 607

BsalGRP94 AIDAKRK-----------EKYEPVLKYFKDLLGE-QVTKVVLTKRKTSEPIILSSRQHDV 633

LmjGRP94 VADRKRK-----------EKYDSFFTHLRVLFGYSEVRKVILTKRMTNEAFIVSSGENQI 635

CfacGRP94 VIDKKRK-----------EKYEALFTRLRTLFGYAEVRKVILTKRLTNEAFILSSGENQI 628

HsHSPC4 SR-EAVE-----------KEFEPLLNWMKDKALKDKIEKAVVSQRLTESPCALVASQYGW 654

HsHSPC1 KQ-EEKK-----------TKFENLCKIMKD-ILEKKVEKVVVSNRLVTSPCCIVTSTYGW 728

HsHSPC3 KM-EESK-----------AKFENLCKLMKE-ILDKKVEKVTISNRLVSSPCCIVTSTYGW 598

BsalHsp83 QK-EEEK-----------ASFEKLCKAMKE-ILGDKVEKVVLTDRLSTSPCILVTSEFGW 573

TbbHsp83-5 QR-EEEK-----------ASYERLCKAMKE-VLGDKVEKVVVSDRLATSPCILVTSEFGW 581

TbbHsp83-9 QR-EEEK-----------ASYERLCKAMKE-VLGDKVEKVVVSDRLATSPCILVTSEFGW 581

TbgHsp83-1 QR-EEEK-----------ASYERLCKAMKE-VLGDKVEKVVVSDRLATSPCILVTSEFGW 581

TbgHsp83-2 QR-EEEK-----------ASYERLCKAMKE-VLGDKVEKVVVSDRLATSPCILVTSEFGW 581

TbgHsp83-3 QR-EEEK-----------ASYERLCKAMKE-VLGDKVEKVVVSDRLATSPCILVTSEFGW 581

TbbHsp83-10 QR-EEEK-----------ASYERLCKAMKE-VLGDKVEKVVVSDRLATSPCILVTSEFGW 581

TbbHsp83-1 QR-EEEK-----------ASYERLCKAMKE-VLGDKVEKVVVSDRLATSPCILVTSEFGW 581

TbbHsp83-2 QR-EEEK-----------ASYERLCKAMKE-VLGDKVEKVVVSDRLATSPCILVTSEFGW 581

TbbHsp83-4 QR-EEEK-----------ASYERLCKAMKE-VLGDKVEKVVVSDRLATSPCILVTSEFGW 581

TbbHsp83-6 QR-EEEK-----------ASYERLCKAMKE-VLGDKVEKVVVSDRLATSPCILVTSEFGW 581

TbbHsp83-7 QR-EEEK-----------ASYERLCKAMKE-VLGDKVEKVVVSDRLATSPCILVTSEFGW 581

TbbHsp83-8 QR-EEEK-----------ASYERLCKAMKE-VLGDKVEKVVVSDRLATSPCILVTSEFGW 581

TbbHsp83-3 QR-EEEK-----------ASYERLCKAMKE-VLGDKVEKVVVSDRLATSPCILVTSEFGW 581

TcMHsp83-2 QR-EEEK-----------AAYERLCKAMKD-VLGDKVEKVVVSERLATSPCILVTSEFGW 581

TcCLBHsp83-2 QR-EEEK-----------TAYERLCKAMKD-VLGDKVEKVVVSERLATSPCILVTSEFGW 581

TcDMHsp83-1 QR-EEEK-----------TAYERLCKAMKD-VLGDKVEKVVVSERLATSPCILVTSEFGW 581

TcDMHsp83-4 QR-EEEK-----------TAYERLCKAMKD-VLGDKVEKVVVSERLATSPCILVTSEFGW 581

TcDMHsp83-5 QR-EEEK-----------TAYERLCKAMKD-VLGDKVEKVVVSERLATSPCILVTSEFGW 581

TcDMHsp83-14 QR-EEEK-----------TAYERLCKAMKD-VLGDKVEKVVVSERLATSPCILVTSEFGW 581

TcDMHsp83-15 QR-EEEK-----------TAYERLCKAMKD-VLGDKVEKVVVSERLATSPCILVTSEFGW 581

TcDMHsp83-16 QR-EEEK-----------TAYERLCKAMKD-VLGDKVEKVVVSERLATSPCILVTSEFGW 581

TcDMHsp83-9 QR-EEEK-----------TAYERLCKAMKD-VLSDKVEKVVVSERLATSPCILVTSEFGW 581

CfacHsp83-1 KR-EEDK-----------AACEKLCKAMKE-ILGDKVEKVAVSERLSTSPCILVTSEFGW 576

CfacHsp83-2 KR-EEDK-----------AACEKLCKAMKE-ILGDKVEKVAVSERLSTSPCILVTSEFGW 576

LmjHsp83-1 QR-EEEK-----------AACEKLCKTMKE-VLGDKVEKVIVSERLSTSPCILVTSEFGW 578

LmjHsp83-2 QR-EEEK-----------AACEKLCKTMKE-VLGDKVEKVIVSERLSTSPCILVTSEFGW 578

LmjHsp83-3 QR-EEEK-----------AACEKLCKTMKE-VLGDKVEKVIVSERLSTSPCILVTSEFGW 578

LmjHsp83-5 QR-EEEK-----------AACEKLCKTMKE-VLGDKVEKVIVSERLSTSPCILVTSEFGW 578

LmjHsp83-6 QR-EEEK-----------AACEKLCKTMKE-VLGDKVEKVIVSERLSTSPCILVTSEFGW 578

LmjHsp83-7 QR-EEEK-----------AACEKLCKTMKE-VLGDKVEKVIVSERLSTSPCILVTSEFGW 578

LmjHsp83-9 QR-EEEK-----------AACEKLCKTMKE-VLGDKVEKVIVSERLSTSPCILVTSEFGW 578

LmjHsp83-10 QR-EEEK-----------AACEKLCKTMKE-VLGDKVEKVIVSERLSTSPCILVTSEFGW 578

LmjHsp83-11 QR-EEEK-----------AACEKLCKTMKE-VLGDKVEKVIVSERLSTSPCILVTSEFGW 578

LmjHsp83-12 QR-EEEK-----------AACEKLCKTMKE-VLGDKVEKVIVSERLSTSPCILVTSEFGW 578

LmjHsp83-13 QR-EEEK-----------AACEKLCKTMKE-VLGDKVEKVIVSERLSTSPCILVTSEFGW 578

LmjHsp83-14 QR-EEEK-----------AACEKLCKTMKE-VLGDKVEKVIVSERLSTSPCILVTSEFGW 578

LmjHsp83-15 QR-EEEK-----------AACEKLCKTMKE-VLGDKVEKVIVSERLSTSPCILVTSEFGW 578

LmjHsp83-16 QR-EEEK-----------AACEKLCKTMKE-VLGDKVEKVIVSERLSTSPCILVTSEFGW 578

LmjHsp83-17 QR-EEEK-----------AACEKLCKTMKE-VLGDKVEKVIVSERLSTSPCILVTSEFGW 578

LmjHsp83-4 QR-EEEK-----------AACEKLCKTMKE-VLGDKVEKVIVSERLSTSPCILVTSEFGW 578

LmjHsp83-8 QR-EEEK-----------AACEKLCKTMKE-VLGDKVEKVIVSERLSTSPCILVTSEFGW 578

. : .: . : * :

MD

HsHSPC5 -GAARHFLRMQQLAKTQ---EERAQLLQPTLEINPRHALIKKLNQLRASE----PGLAQL 667

LmjTRAP1 -AQMRKIYRMTGQAAG--------PPPKYNLHFNPQHPLIRKLYTLSQSEASEEVETAGL 716

CfacTRAP1 -AQMRKIYRMTGQSAG--------PPPKYNLHFNPQHPLIRKLYTLSQSESSEDVETAGL 717

BsalTRAP1 -AQMRKIYRVTGQASG--------PAPKYNLHFNPQHELIRKMYTLSVSAANEEVETAGI 633

TbbTRAP1 -AQMRKIYRITGQMAG--------APPKYNMHFNPKHTIVRKLYTLSISPNSEEVETAGL 709

TbgTRAP1 -AQMRKIYRITGQMAG--------APPKYNMHFNPKHTIVRKLYTLSISPNSEEVETAGL 709

TcMTRAP1 -AQMRKIYRVTGQIAG--------PPPKYNFHFNPKHPIVRKLYTLSISPAAEEVETAGL 705

TcCLBTRAP1 -AQMRKIYRVTGQMAG--------PPPKYNFHFNPKHPIVRKLYTLSISPAAEEVETAGL 706

TcDMTRAP1 -AQMRKIYRVTGQMAG--------PPPKYNFHFNPKHPIVRKLYTLSISPTTEEVETAGL 707

TbbGRP94 SPRLANIIKQQAVS---SDH---SVFHTLVLEINYRHPVVQQLLARFQA--NANDQVALD 663

TbgGRP94 SPRLANIIKQQAVS---SDH---SVFHTLVLEINYRHPVVQQLLARFQA--NANDQVALD 663

TcMGRP94 TPRMVNVMNQQAIS---SLQ---TMRYSRVLELNYRHPLVRDLLTRFEA--DSNDQTAID 659

TcCLBGRP94 TPRMVNVMNQQAIS---SLH---TMRYSRVLELNHRHPLVRDLLTRFEA--DANDQTAID 659

TcDMGRP94 TPRMVSVMNQQAIS---SLH---TMRYSRVLELNHRHPLVRDLLTRFGA--DANDQTAID 659

BsalGRP94 TARMANIIRGQALG---DAKQNEAQTAKRVMEINHLHPLIEEIFKRVKA--DDKDKVAED 688

LmjGRP94 TARLASIMRGQSMS---LANQ--QMTAERVLEVNYRHPLVDEMFKRFTV--DENDEVATD 688

CfacGRP94 TARLANIMRGQSMA---LVDQ--QTAAERVLEVNYRHPLVEEMFKRFAV--DEEDEVAVD 681

HsHSPC4 SGNMERIMKAQAYQTGKDISTNYYASQKKTFEINPRHPLIRDMLRRIKE--DEDDKTVLD 712

HsHSPC1 TANMERIMKAQALR---DNSTMGYMAAKKHLEINPDHSIIETLRQKAEA--DKNDKSVKD 783

HsHSPC3 TANMERIMKAQALR---DNSTMGYMMAKKHLEINPDHPIVETLRQKAEA--DKNDKAVKD 653

BsalHsp83 SAHMEQIMRNQALR---DSSMSSYMMSKKTMEINSKHGIVRELRRRVEN--EQNDKAVKD 628

TbbHsp83-5 SAHMEQIMRNQALR---DSSMSAYMMSKKTMEINTTHAIVKELKRRVEA--DENDKAAKD 636

TbbHsp83-9 SAHMEQIMRNQALR---DSSMSAYMMSKKTMEINTTHAIVKELKRRVEA--DENDKAAKD 636

TbgHsp83-1 SAHMEQIMRNQALR---DSSMSAYMMSKKTMEINTTHAIVKELKRRVEA--DENDKAAKD 636

TbgHsp83-2 SAHMEQIMRNQALR---DSSMSAYMMSKKTMEINTTHAIVKELKRRVEA--DENDKAAKD 636

TbgHsp83-3 SAHMEQIMRNQALR---DSSMSAYMMSKKTMEINTTHAIVKELKRRVEA--DENDKAAKD 636

TbbHsp83-10 SAHMEQIMRNQALR---DSSMSAYMMSKKTMEINTTHAIVKELKRRVEA--DENDKAAKD 636

TbbHsp83-1 SAHMEQIMRNQALR---DSSMSAYMMSKKTMEINTTHAIVKELKRRVEA--DENDKAAKD 636

TbbHsp83-2 SAHMEQIMRNQALR---DSSMSAYMMSKKTMEINTTHAIVKELKRRVEA--DENDKAAKD 636

TbbHsp83-4 SAHMEQIMRNQALR---DSSMSAYMMSKKTMEINTTHAIVKELKRRVEA--DENDKAAKD 636

TbbHsp83-6 SAHMEQIMRNQALR---DSSMSAYMMSKKTMEINTTHAIVKELKRRVEA--DENDKAAKD 636

TbbHsp83-7 SAHMEQIMRNQALR---DSSMSAYMMSKKTMEINTTHAIVKELKRRVEA--DENDKAAKD 636

TbbHsp83-8 SAHMEQIMRNQALR---DSSMSAYMMSKKTMEINTTHAIVKELKRRVEA--DENDKAAKD 636

TbbHsp83-3 SAHMEQIMRNQALR---DSSMSAYMMSKKTMEINTTHAIVKELKRRVEA--DENDKAAKD 636

TcMHsp83-2 SAHMEQIMRNQALR---DSSMSAYMMSKKTMEINPAHPIVKELKRRVEA--DENDKAVKD 636

TcCLBHsp83-2 SAHMEQIMRNQALR---DSSMSAYMMSKKTMEINPAHPIVKELKRRVEA--DENDKAVKD 636

TcDMHsp83-1 SAHMEQIMRNQALR---DSSMSAYMMSKKTMEINPAHPIVKELKRRVEA--DENDKAVKD 636

TcDMHsp83-4 SAHMEQIMRNQALR---DSSMSAYMMSKKTMEINPAHPIVKELKRRVEA--DENDKAVKD 636

TcDMHsp83-5 SAHMEQIMRNQALR---DSSMSAYMMSKKTMEINPAHPIVKELKRRVEA--DENDKAVKD 636

TcDMHsp83-14 SAHMEQIMRNQALR---DSSMSAYMMSKKTMEINPAHPIVKELKRRVEA--DENDKAVKD 636

TcDMHsp83-15 SAHMEQIMRNQALR---DSSMSAYMMSKKTMEINPAHPIVKELKRRVEA--DENDKAVKD 636

TcDMHsp83-16 SAHMEQIMRNQALR---DSSMSAYMMSKKTMEINPAHPIVKELKRRVEA--DENDKAVKD 636

TcDMHsp83-9 SAHMEQIMRNQALR---DSSMSAYMMSKKTMEINPAHPIVKELKRRVEA--DENDKAVKD 636

CfacHsp83-1 SAHMEQIMRNQALR---DSSMAQYMMSKKTMELNPGHPIIKELRRRVEA--DENDKAVKD 631

CfacHsp83-2 SAHMEQIMRNQALR---DSSMAQYMMSKKTMELNPGHPIIKELRRRVEA--DENDKAVKD 631

LmjHsp83-1 SAHMEQIMRNQALR---DSSMAQYMMSKKTMELNPRHPIIKELRRRVGA--DENDKAVKD 633

LmjHsp83-2 SAHMEQIMRNQALR---DSSMAQYMMSKKTMELNPRHPIIKELRRRVGA--DENDKAVKD 633

LmjHsp83-3 SAHMEQIMRNQALR---DSSMAQYMMSKKTMELNPRHPIIKELRRRVGA--DENDKAVKD 633

LmjHsp83-5 SAHMEQIMRNQALR---DSSMAQYMMSKKTMELNPRHPIIKELRRRVGA--DENDKAVKD 633

LmjHsp83-6 SAHMEQIMRNQALR---DSSMAQYMMSKKTMELNPRHPIIKELRRRVGA--DENDKAVKD 633

LmjHsp83-7 SAHMEQIMRNQALR---DSSMAQYMMSKKTMELNPRHPIIKELRRRVGA--DENDKAVKD 633

LmjHsp83-9 SAHMEQIMRNQALR---DSSMAQYMMSKKTMELNPRHPIIKELRRRVGA--DENDKAVKD 633

LmjHsp83-10 SAHMEQIMRNQALR---DSSMAQYMMSKKTMELNPRHPIIKELRRRVGA--DENDKAVKD 633

LmjHsp83-11 SAHMEQIMRNQALR---DSSMAQYMMSKKTMELNPRHPIIKELRRRVGA--DENDKAVKD 633

LmjHsp83-12 SAHMEQIMRNQALR---DSSMAQYMMSKKTMELNPRHPIIKELRRRVGA--DENDKAVKD 633

LmjHsp83-13 SAHMEQIMRNQALR---DSSMAQYMMSKKTMELNPRHPIIKELRRRVGA--DENDKAVKD 633

LmjHsp83-14 SAHMEQIMRNQALR---DSSMAQYMMSKKTMELNPRHPIIKELRRRVGA--DENDKAVKD 633

LmjHsp83-15 SAHMEQIMRNQALR---DSSMAQYMMSKKTMELNPRHPIIKELRRRVGA--DENDKAVKD 633

LmjHsp83-16 SAHMEQIMRNQALR---DSSMAQYMMSKKTMELNPRHPIIKELRRRVGA--DENDKAVKD 633

LmjHsp83-17 SAHMEQIMRNQALR---DSSMAQYMMSKKTMELNPRHPIIKELRRRVGA--DENDKAVKD 633

LmjHsp83-4 SAHMEQIMRNQALR---DSSMAQYMMSKKTMELNPRHPIIKELRRRVGA--DENDKAVKD 633

LmjHsp83-8 SAHMEQIMRNQALR---DSSMAQYMMSKKTMELNPRHPIIKELRRRVGA--DENDKAVKD 633

. . :..* * :: : .

CTD

HsHSPC5 LVDQIYENAMIAAGL-VDDPRAMVGRLNELLVKALERH---------------------- 704

LmjTRAP1 LTEQIFDNAIIAAGL-LEDPRSIVTRLNTIMSRMVEKVPEPSADQ--------------- 760

CfacTRAP1 LAEQVFDNAVIAAGL-LEDPRSIVSRLNTIMSRMVEKVPEPSADK--------------- 761

BsalTRAP1 LAEQLFDNALIAAGL-MEDPRSIVQRLNTIMNKMVKDVAEPTADK--------------- 677

TbbTRAP1 LVEQMFDNAVIAAGL-LEDPRSIVSRLNTIMTRMVENVEEPTADK--------------- 753

TbgTRAP1 LVEQMFDNAVIAAGL-LEDPRSIVSRLNTIMTRMVENVEEPTADK--------------- 753

TcMTRAP1 LVEQLFDNAVISAGL-LEDPRSIVLRLNNIMSRMVENVPEPTADK--------------- 749

TcCLBTRAP1 LVEQLFDNAVISAGL-LEDPRSIVSRLNNIMSRMVENVPEPTADK--------------- 750

TcDMTRAP1 LVEQLFDNAVISAGL-LEDPRSIVSRLNNIMSRMVENVPEPTADK--------------- 751

TbbGRP94 IAWVLFGTASLQADSPVPDQAMYAKRVTRLVRGRMDLPLDDALLPPDDNEYDVEG--VKP 721

TbgGRP94 IAWVLFGTASLQADSPVPDQAMYAKRVTRLVRGRMDLPLDDALLPPDDNEYDVEG--VKP 721

TcMGRP94 VAWVLFGTANMQAGFPVSNQAMYAKRVNRLLRGRVGLAADDTMLPPDDDEYDISE--VKP 717

TcCLBGRP94 VAWVLFGTANLQAGFPVSNQAMYAKRVNRLLRGRVGLAADDTILPPDDDEYDISD--VKP 717

TcDMGRP94 VAWVLFGTANLQAGFPVSNQAMYAKRVNRLLRGRVGLAADDTILPPDDDEYDISD--VKP 717

BsalGRP94 VALVLFDTANLQNGFDIEDTLAFSRRMSRLLRQSVDIPADAAMLTEDVSEYEIED--NED 746

LmjGRP94 IAWVLYDTANLQAEFPVADVAAYSKRINRLLRSSVDLSADDSLLPPDDAEYTVSD--TEA 746

CfacGRP94 IAWVLYDTANLQAEFPVADVAAYSRRINRLLRSSVDMPADDTLLPPDDAEYAVSN--TET 739

HsHSPC4 LAVVLFETATLRSGYLLPDTKAYGDRIERMLRLSLNIDPDAKVEEEPEEEPEETAEDTTE 772

HsHSPC1 LVILLYETALLSSGFSLEDPQTHANRIYRMIKLGLGIDEDDPTADDTSAAVTEEMPPLEG 843

HsHSPC3 LVVLLFETALLSSGFSLEDPQTHSNRIYRMIKLGLGIDEDEVAAEEPNAAVPDEIPPLEG 713

BsalHsp83 LVYLLFDTSLLTSGFNLEDPTAYAERIHRMIKLGLSLDDEEEVEAAPAAA-PV----PAA 683

TbbHsp83-5 LIFLLFDTSLLTSGFTLDDPTAYADRIHRMIKLGLSLDDDAEEEEAQ--A-PVAAAAANS 693

TbbHsp83-9 LIFLLFDTSLLTSGFTLDDPTAYADRIHRMIKLGLSLDDDAEEEEAQ--A-PVAAAAANS 693

TbgHsp83-1 LIFLLFDTSLLTSGFTLDDPTAYADRIHRMIKLGLSLDDDAEEEEAQ--A-PVAAAAANS 693

TbgHsp83-2 LIFLLFDTSLLTSGFTLDDPTAYADRIHRMIKLGLSLDDDAEEEEAQ--A-PVAAAAANS 693

TbgHsp83-3 LIFLLFDTSLLTSGFTLDDPTAYADRIHRMIKLGLSLDDDAEEEEAQ--A-PVAAAAANS 693

TbbHsp83-10 LIFLLFDTSLLTSGFTLDDPTAYADRIHRMIKLGLSLDDDAEEEEAQ--A-PVAAAAANS 693

TbbHsp83-1 LIFLLFDTSLLTSGFTLDDPTAYADRIHRMIKLGLSLDDDAEEEEAQ--A-PVAAAAANS 693

TbbHsp83-2 LIFLLFDTSLLTSGFTLDDPTAYADRIHRMIKLGLSLDDDAEEEEAQ--A-PVAAAAANS 693

TbbHsp83-4 LIFLLFDTSLLTSGFTLDDPTAYADRIHRMIKLGLSLDDDAEEEEAQ--A-PVAAAAANS 693

TbbHsp83-6 LIFLLFDTSLLTSGFTLDDPTAYADRIHRMIKLGLSLDDDAEEEEAQ--A-PVAAAAANS 693

TbbHsp83-7 LIFLLFDTSLLTSGFTLDDPTAYADRIHRMIKLGLSLDDDAEEEEAQ--A-PVAAAAANS 693

TbbHsp83-8 LIFLLFDTSLLTSGFTLDDPTAYADRIHRMIKLGLSLDDDAEEEEAQ--A-PVAAAAANS 693

TbbHsp83-3 LIFLLFDTSLLTSGFTLDDPTAYADRIHRMIKLGLSLDDDAEEEEAQ--A-PVAAAAANS 693

TcMHsp83-2 LVYLLFDTALLTSGFTLDDPTSYAERIHRMIKLGLSLDDEDNGNEES--E-PAAAVPAES 693

TcCLBHsp83-2 LVYLLFDTALLTSGFTLDDPTSYAERIHRMIKLGLSLDDEDNGNEEA--E-PAAAVPAEP 693

TcDMHsp83-1 LVYLLFDTALLTSGFTLDDPTSYAERIHRMIKLGLSLDDEDNGNEEA--E-PAAAVPAEP 693

TcDMHsp83-4 LVYLLFDTALLTSGFTLDDPTSYAERIHRMIKLGLSLDDEDNGNEEA--E-PAAAVPAEP 693

TcDMHsp83-5 LVYLLFDTALLTSGFTLDDPTSYAERIHRMIKLGLSLDDEDNGNEEA--E-PAAAVPAEP 693

TcDMHsp83-14 LVYLLFDTALLTSGFTLDDPTSYAERIHRMIKLGLSLDDEDNGNEEA--E-PAAAVPAEP 693

TcDMHsp83-15 LVYLLFDTALLTSGFTLDDPTSYAERIHRMIKLGLSLDDEDNGNEEA--E-PAAAVPAEP 693

TcDMHsp83-16 LVYLLFDTALLTSGFTLDDPTSYAERIHRMIKLGLSLDDEDNGNEEA--E-PAAAVPAEP 693

TcDMHsp83-9 LVYLLFDTALLTSGFTLDDPTSYAERIHRMIKLGLSLDDEDNGNEEA--E-PAAAVPAEP 693

CfacHsp83-1 LVFLLFDTSLLTSGFQLDDPTGYAERINRMIKLGLSLDDEEEAAPAEAAP-AAEAAPVEA 690

CfacHsp83-2 LVFLLFDTSLLTSGFQLDDPTGYAERINRMIKLGLSLDDEEEAAPAEAAP-AAEAAPVEA 690

LmjHsp83-1 LVFLLFDTSLLTSGFQLEDPTGYAERINRMIKLGLSLDEEEEEA--AEAP-VAETAPAEV 690

LmjHsp83-2 LVFLLFDTSLLTSGFQLEDPTGYAERINRMIKLGLSLDEEEEEA--AEAP-VAETAPAEV 690

LmjHsp83-3 LVFLLFDTSLLTSGFQLEDPTGYAERINRMIKLGLSLDEEEEEA--AEAP-VAETAPAEV 690

LmjHsp83-5 LVFLLFDTSLLTSGFQLEDPTGYAERINRMIKLGLSLDEEEEEA--AEAP-VAETAPAEV 690

LmjHsp83-6 LVFLLFDTSLLTSGFQLEDPTGYAERINRMIKLGLSLDEEEEEA--AEAP-VAETAPAEV 690

LmjHsp83-7 LVFLLFDTSLLTSGFQLEDPTGYAERINRMIKLGLSLDEEEEEA--AEAP-VAETAPAEV 690

LmjHsp83-9 LVFLLFDTSLLTSGFQLEDPTGYAERINRMIKLGLSLDEEEEEA--AEAP-VAETAPAEV 690

LmjHsp83-10 LVFLLFDTSLLTSGFQLEDPTGYAERINRMIKLGLSLDEEEEEA--AEAP-VAETAPAEV 690

LmjHsp83-11 LVFLLFDTSLLTSGFQLEDPTGYAERINRMIKLGLSLDEEEEEA--AEAP-VAETAPAEV 690

LmjHsp83-12 LVFLLFDTSLLTSGFQLEDPTGYAERINRMIKLGLSLDEEEEEA--AEAP-VAETAPAEV 690

LmjHsp83-13 LVFLLFDTSLLTSGFQLEDPTGYAERINRMIKLGLSLDEEEEEA--AEAP-VAETAPAEV 690

LmjHsp83-14 LVFLLFDTSLLTSGFQLEDPTGYAERINRMIKLGLSLDEEEEEA--AEAP-VAETAPAEV 690

LmjHsp83-15 LVFLLFDTSLLTSGFQLEDPTGYAERINRMIKLGLSLDEEEEEA--AEAP-VAETAPAEV 690

LmjHsp83-16 LVFLLFDTSLLTSGFQLEDPTGYAERINRMIKLGLSLDEEEEEA--AEAP-VAETAPAEV 690

LmjHsp83-17 LVFLLFDTSLLTSGFQLEDPTGYAERINRMIKLGLSLDEEEEEA--AEAP-VAETAPAEV 690

LmjHsp83-4 LVFLLFDTSLLTSGFQLEDPTGYAERINRMIKLGLSLDEEE-EA--AEAP-VAETAPAEV 689

LmjHsp83-8 LVFLLFDTSLLTSGFQLEDPTGYAERINRMIKLGLSLDEEE-EA--AEAP-VAETAPAEV 689

: :: .: : : : *: :: :

CTD

HsHSPC5 ----------------------------------------------------- 704

LmjTRAP1 ----------------------------------------------------- 760

CfacTRAP1 ----------------------------------------------------- 761

BsalTRAP1 ----------------------------------------------------- 677

TbbTRAP1 ----------------------------------------------------- 753

TbgTRAP1 ----------------------------------------------------- 753

TcMTRAP1 ----------------------------------------------------- 749

TcCLBTRAP1 ----------------------------------------------------- 750

TcDMTRAP1 ----------------------------------------------------- 751

TbbGRP94 D-TVDSEEEVLLPVDNDEEGTKGKSAEKGQKKSSEKVEKKPGKKSTGANAGDL 773

TbgGRP94 D-TVDSEEEVLLPVDNDEEGTKGKSAEKGQKKSSEKVEKKPGKKSTGANAGDL 773

TcMGRP94 D-TTGTDEGLLLPVDKEGDESPDKEDAEP-----TAAETKPT--KTEDDAGDL 762

TcCLBGRP94 D-TTGTDEGLLLPVDNDGDESSDKEDAEP-----TAAEMKPT--KTEDDAGDL 762

TcDMGRP94 D-STGTDEGLLLPVDNDGDESSEKEDAEP-----TAAETKPT--KTEDDAGDL 762

BsalGRP94 A-EDDEAPKA--DADDDKEEL-------------------------------- 764

LmjGRP94 E-EEEEQPKV--DANADEK-------------------------AEAVDEGDL 771

CfacGRP94 A-EEEEGDAP--GGIVEANAD----------------------ADVEEDEGDL 767

HsHSPC4 DTEQDEDEEM--DVGTDEEEETAKEST--------------------AEKDEL 803

HsHSPC1 DDDTSRMEEV--D---------------------------------------- 854

HsHSPC3 DEDASRMEEV--D---------------------------------------- 724

BsalHsp83 TTGASSMESV--D---------------------------------------- 694

TbbHsp83-5 STGASGMEEV--D---------------------------------------- 704

TbbHsp83-9 STGASGMEEV--D---------------------------------------- 704

TbgHsp83-1 STGASGMEEV--D---------------------------------------- 704

TbgHsp83-2 STGASGMEEV--D---------------------------------------- 704

TbgHsp83-3 STGASGMEEV--D---------------------------------------- 704

TbbHsp83-10 STGASGMEEV--D---------------------------------------- 704

TbbHsp83-1 STGASGMEEV--D---------------------------------------- 704

TbbHsp83-2 STGASGMEEV--D---------------------------------------- 704

TbbHsp83-4 STGASGMEEV--D---------------------------------------- 704

TbbHsp83-6 STGASGMEEV--D---------------------------------------- 704

TbbHsp83-7 STGASGMEEV--D---------------------------------------- 704

TbbHsp83-8 STGASGMEEV--D---------------------------------------- 704

TbbHsp83-3 STGASGMEEV--D---------------------------------------- 704

TcMHsp83-2 VAGTSSMEQV--D---------------------------------------- 704

TcCLBHsp83-2 VAGTSSMELV--D---------------------------------------- 704

TcDMHsp83-1 VAGTSSMEQV--D---------------------------------------- 704

TcDMHsp83-4 VAGTSSMEQV--D---------------------------------------- 704

TcDMHsp83-5 VAGTSSMEQV--D---------------------------------------- 704

TcDMHsp83-14 VAGTSSMEQV--D---------------------------------------- 704

TcDMHsp83-15 VAGTSSMEQV--D---------------------------------------- 704

TcDMHsp83-16 VAGTSSMEQV--D---------------------------------------- 704

TcDMHsp83-9 VAGTSSMEQV--D---------------------------------------- 704

CfacHsp83-1 TAGTSSMEQV--D---------------------------------------- 701

CfacHsp83-2 TAGTSSMEQV--D---------------------------------------- 701

LmjHsp83-1 TAGTSSMEQV--D---------------------------------------- 701

LmjHsp83-2 TAGTSSMEQV--D---------------------------------------- 701

LmjHsp83-3 TAGTSSMEQV--D---------------------------------------- 701

LmjHsp83-5 TAGTSSMEQV--D---------------------------------------- 701

LmjHsp83-6 TAGTSSMEQV--D---------------------------------------- 701

LmjHsp83-7 TAGTSSMEQV--D---------------------------------------- 701

LmjHsp83-9 TAGTSSMEQV--D---------------------------------------- 701

LmjHsp83-10 TAGTSSMEQV--D---------------------------------------- 701

LmjHsp83-11 TAGTSSMEQV--D---------------------------------------- 701

LmjHsp83-12 TAGTSSMEQV--D---------------------------------------- 701

LmjHsp83-13 TAGTSSMEQV--D---------------------------------------- 701

LmjHsp83-14 TAGTSSMEQV--D---------------------------------------- 701

LmjHsp83-15 TAGTSSMEQV--D---------------------------------------- 701

LmjHsp83-16 TAGTSSMEQV--D---------------------------------------- 701

LmjHsp83-17 TAGTSSMEQV--D---------------------------------------- 701

LmjHsp83-4 TAGTSSMEQV--D---------------------------------------- 700

LmjHsp83-8 TAGTSSMEQV--D---------------------------------------- 700

**Figure S1:** Alignment of the Hsp90/HSPC complement from *T. brucei* in relation to human and other selected trypanosomatids.

Multiple sequence alignment of the full-length amino acid sequences was performed using the in-built ClustalW program (Larkin et al., 2007) with default parameters in the MEGA X software (Kumar et al., 2018). Degree of amino acid conservation is symbolized by the following: (*) all fully conserved residues; (:) one of the residues is fully conserved and (.) residues are weakly conserved. The C-terminus motifs are empty-boxed in magenta for the cytosolic Hsp90 and light blue for the mitochondrial TRAP-1. SP-signal peptide; ND-N-terminal domain; CL-charged linker domain; MD-middle domain and CTD-C-terminal domain.
